# Europe’s bottlenecks to the operationalisation of Essential Biodiversity Variables for Environmental Policy Support

**DOI:** 10.1038/s44185-026-00147-4

**Published:** 2026-07-09

**Authors:** Alejandra Morán-Ordóñez, Maria Blasi, Joana Santana, Pedro Beja, Tom D. Breeze, Laurence Carvalho, Ana Cristina Cardoso, Sara Fraixedas, Gabriel Gargallo, Eugenio Gervasini, Sergi Herrando, Borja Jiménez Alfaro, Jessi Junker, W. Daniel Kissling, Vujadin Kovacevic, Tom Langendoen, Adrià López-Baucells, Maria Lumbierres, Anne Lyche Solheim, Chiara Magliozzi, Sophie Mentzel, Gabriel Miret-Minard, S. Jannicke Moe, Francisco Moreira, Diego Pavón-Jordán, Henrique M. Pereira, Simon G. Potts, Judy Shamoun-Baranes, Roy H. A. van Grunsven, Dani Villero, Lluís Brotons

**Affiliations:** 1 Department of Environmental Sciences, Universität Basel, Vesalgasse 1, 4051 Basel, Switzerland; 2 Centre for Ecological Research and Forestry Applications (CREAF), Cerdanyola del Vallès, Spain; 3 Centro de Investigação em Biodiversidade e Recursos Genéticos (CIBIO), InBIO Laboratório Associado, Campus de Vairão, Universidade do Porto, Vairão, Portugal; 4 Program in Genomics, Biodiversity and Land Planning (BIOPOLIS), Campus de Vairão, Universidade do Porto, Vairão, Portugal; 5 Centro de Investigação em Biodiversidade e Recursos Genéticos (CIBIO), InBIO Laboratório Associado, Instituto Superior de Agronomia , Universidade de Lisboa, Lisbon, Portugal; 6 Centre for Agri-Environmental Research (CAER), School of Agriculture, Policy and Development, Reading University, Reading, UK; 7 Norwegian Institute for Water Research (NIVA), Sognsveien 72, 0855 Oslo, Norway; 8 European Commission, Joint Research Centre (JRC), Ispra, Italy; 9 Catalan Ornithological Institute (ICO), Natural Science Museum of Barcelona, Barcelona, Spain; 10 European Bird Census Council (EBCC), Prague, Czech Republic; 11 Instituto Mixto de Investigación en Biodiversidad, Universidad de Oviedo, Oviedo, Asturias Spain; 12 Re:wild, Austin, TX USA; 13 Senckenberg Museum for Natural History Görlitz, Görlitz, Germany; 14 German Centre for Integrative Biodiversity Research (iDiv) Halle-Jena-Leipzig, Leipzig, Germany; 15 Institute for Biodiversity and Ecosystem Dynamics (IBED), University of Amsterdam, P.O. Box 94240, 1090 GE Amsterdam, The Netherlands; 16 Directorate General for Environment, European Commission, Brussels, Belgium; 17 Wetlands International, Horapark 9, Ede, 6717 LZ The Netherlands; 18 BiBio Research Group, Natural Sciences Museum of Granollers, Granollers, Spain; 19 BETA Technological Centre, Vic, Spain; 20 Universitat de Vic – Universitat Central de Catalunya (UVic-UCC), Vic, Spain; 21 Forest Science and Technology Centre of Catalonia (CTFC), 25280 Solsona Lleida, Spain; 22 Biological Station of Mértola (EBM), CIBIO, Praça Luís de Camões, Mértola, Mértola, Portugal; 23 Birds and Renewables Research Group, Norwegian Institute for Nature Research (NINA), Trondheim, Norway; 24 Dept. of Zoology, Institute of Biology, Martin Luther University Halle-Wittenberg, Halle (Saale), Germany; 25 Dutch Butterfly Conservation, Wageningen, The Netherlands; 26 The Spanish National Research Council (CSIC), Cerdanyola del Vallès, Spain

**Keywords:** Ecology, Ecology, Environmental sciences

## Abstract

The operationalisation of Essential Biodiversity Variables (EBVs) is critical for assessing whether conservation and restoration measures effectively meet the biodiversity objectives outlined in national and international commitments such as the Kunming-Montreal Global Biodiversity Framework. EBVs provide a standardized framework of biological measurements that enable the evaluation, reporting, and management of biodiversity changes across time, space, and biological levels. At the EU scale, the Europa Biodiversity Observation Network (EuropaBON) identified 84 EBVs that are key to supporting EU environmental policies. Here, we evaluated 17 coordinated European monitoring programmes to identify bottlenecks in biodiversity data flows that could operationalise EBVs across freshwater, terrestrial, and marine realms in Europe. We evaluated these bottlenecks using a qualitative framework applicable across taxonomic, spatio-temporal, and ecosystem scales. Our analysis identified several persistent challenges to the operationalisation of EBVs at supranational scales: misalignment between monitoring data outputs and EBV definitions; insufficient taxonomic, spatial, and temporal coverage; limited open access to raw data and data products, and incomplete automation of end-to-end data workflows. Certain taxonomic groups—including freshwater zooplankton, lichens, terrestrial arthropods, and crop pests—remain underrepresented, while marine EBVs are constrained by fragmented monitoring and inconsistent protocols. To overcome these challenges, securing long-term funding, coordinated hubs, harmonized protocols, and enhanced data integration are essential. These measures will enable the delivery of EBVs in Europe that comply with FAIR (Findable, Accessible, Interoperable, Reusable) and CARE (Collective Benefit, Authority to Control, Responsibility, Ethics) principles, supporting robust biodiversity policy assessments in Europe.

## Introduction

The unprecedented rate of biodiversity loss and ecosystem degradation, and destruction^1^ has prompted a decade of robust political and institutional commitments seeking to halt and reverse these alarming trends. Different agendas and regulations have established specific conservation and restoration targets for the end of this decade. For example, the Kunming-Montreal Global Biodiversity Framework^2^ includes commitments to restore 30% of degraded ecosystems and conserve 30% of land and sea. These global targets have also been incorporated into European legislation, such as the recently approved EU Restoration Regulation (Regulation (EU) 2024/1991), which envisions restoring all degraded EU ecosystems by 2050. Among the many challenges associated with these ambitious objectives, there is a need to define a set of indicators that can effectively evaluate the success of the implemented conservation and restoration measures and to monitor progress toward goal achievement. This includes the need to collect and harmonize data to inform those indicators^3–6^ .

The Essential Biodiversity Variables (EBVs) framework^7,8^, developed by the Group on Earth Observations Biodiversity Observation Network (GEO BON) has been proposed to serve this purpose^3,4,6,9^. This framework proposes a list of 21 generic EBVs or a minimum set of standardized biological measurements to evaluate and monitor changes in biodiversity across time, space, and biological levels. These 21 EBVs inform about biodiversity change at different levels and are grouped into six major classes to capture major biodiversity dimensions: species-focused EBVs (genetic composition, species traits, species populations) and ecosystem-level EBVs (community composition, ecosystem functioning, and ecosystem structure). EBVs facilitate data integration in a spatially- and temporally explicit way and represent an intermediate abstraction level between primary observations (both from field and remote sensing data) and indicators^7,10^. However, our ability to compute spatially- and temporally-explicit EBVs depends on factors including the accessibility, nature, and quality of data available^11,12^, the existence of well-established and replicable data workflows, the establishment of data standards, technical capacity, and infrastructure to harmonize and integrate data across different sources^9,13^.

In recent decades, the European Commission has made substantial progress towards coordinating and harmonising biodiversity and ecosystems data, especially those related to the reporting of the conservation status of species and habitats linked to the Habitats and Birds Directives (Council Directive 92/43/EEC, Directive 2009/147/EC, respectively), and the Water Framework Directive (Directive 2000/60/EC). However, biodiversity monitoring efforts across Europe remain spatially and temporally fragmented and taxonomically biased across Europe and poorly standardized and integrated among member states^6,12,14^. To address these challenges the European Commission mandated the Europa Biodiversity Observation Network (EuropaBON) network to develop foundational backbone for an integrated biodiversity monitoring framework across Europe^4,15^. This network – integrated by a large community of >1600 European stakeholders from nearly 1000 organizations—including academia, government, NGOs, the private sector, and citizen science—with expertise spanning terrestrial, freshwater, and marine realms, as well as diverse geographic regions^16^– sought to co-design a cost-efficient framework, aligning policies, EBVs, realms and monitoring methods into a coherent system that allows tracking progress of biodiversity-oriented policy instruments towards environmental protection^4^. Building on the EBVs framework developed by GEOBON, the EuropaBON network defined a specific list of 84 EBVs tailored to meet European monitoring needs and inform biodiversity policy and management^4,17^. This list outlines a minimum set of standardized variables required to quantitatively measure changes in biodiversity - across multiple taxa, ecosystems and realms (terrestrial, freshwater, marine) - and the delivery of ecosystem services over time in Europe^4–6,12,17^. Each EBV was classified under one of the six general EBV classes and is characterized by specific taxonomic, spatial, and temporal dimensions. For example, within the marine realm and under the broad EBV class “species populations,” stakeholders identified the need to generate eight distinct EBVs related to “species distributions.” These include variables such as “species distributions of marine fishes” and “species distributions of marine turtles,” each with unique spatial resolutions and temporal frequencies.

The EuropaBON framework then evaluated key aspects of data collection, processing and management, as well as relevant workflows, and analytical needs for integrating and reporting biodiversity trends in key ecosystems across Europe, based on the defined EBVs^4,5,12^. This assessment led the foundation for a proposal for an EU Biodiversity Observation Coordination Centre (EBOCC). The EBOCC would coordinate and promote the generation and use of high-quality biodiversity data to strengthen the knowledge base underpinning EU policies, while also providing guidance and training as needed^4,18^. In response to this proposal, the European Commission launched a Call for Tenders, and a winning consortium was selected to pilot the EBOCC starting in 2026.

Within this framework and in this study, we sought to identify and evaluate major limitations (bottlenecks) in the capacity to operationalise EBVs at the European level from already existing biodiversity monitoring schemes in Europe. Specifically, we assessed how well existing biodiversity monitoring schemes are equipped to capture and collect the data required to produce the EBVs proposed by the EuropaBON network. In other words: Are current monitoring programs ready to deliver the necessary information for operationalising those EBVs?

We considered a bottleneck any aspect or factor that hinders or constrains the quantification or generation of a given EBV at the desired temporal frequency and spatial resolution. Past efforts aiming at describing the main factors hampering the integration and use of biodiversity monitoring data have mostly focused on specific constraints in isolation, e.g., taxonomic, geographic, and data accessibility gaps^12,19^, rarely acknowledging the multifactorial nature of successful biodiversity monitoring workflows^4,5,20^. Therefore, there was a need for an integrative analysis that adequately assessed the diversity of factors at play in biodiversity monitoring workflows and evaluated them across a wide range of monitoring flows that could be very different in practice (data pipelines, integration tools, funding, technical capacity, etc.).

**Fig. 1:**
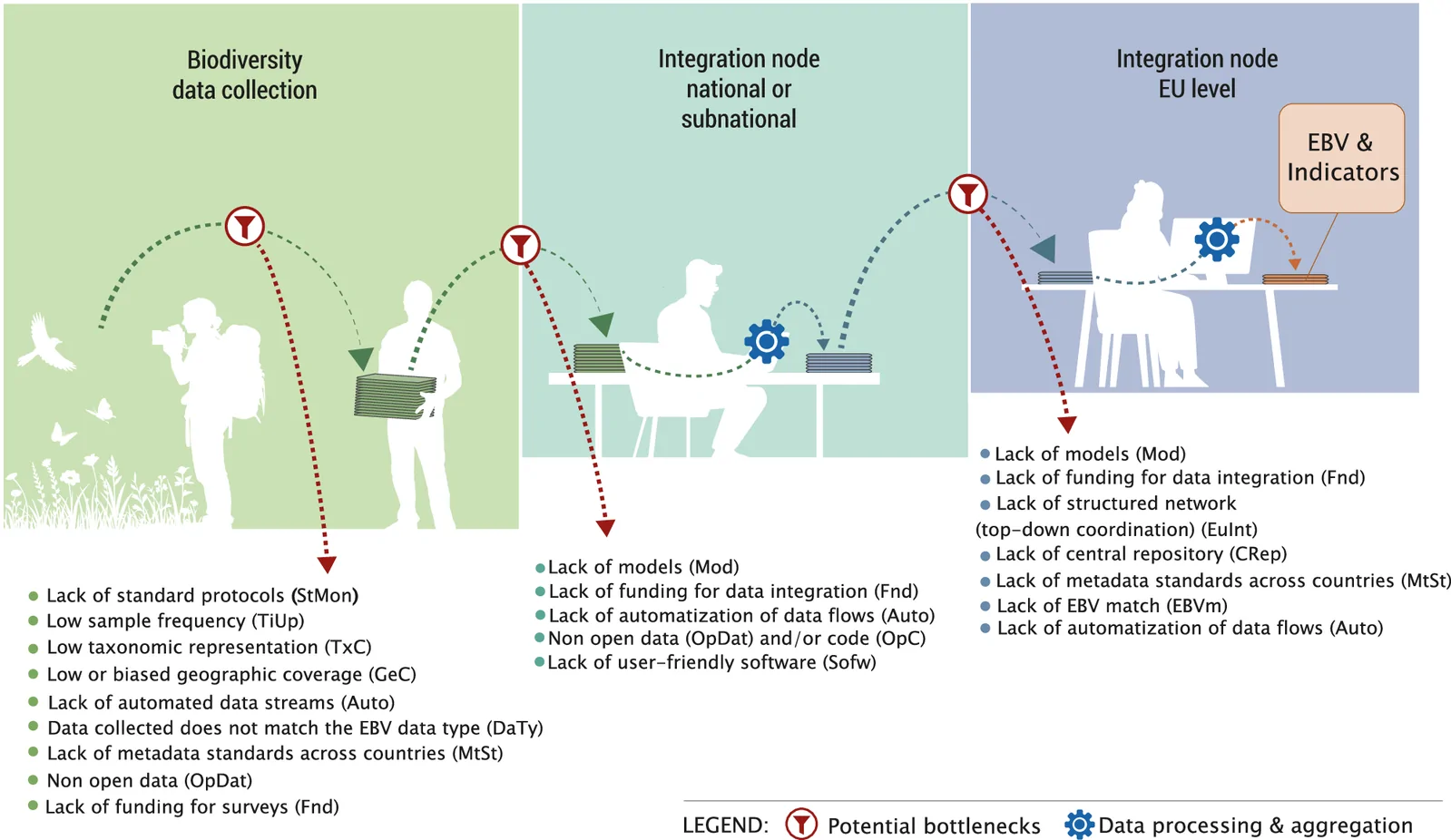
Examples of potential bottlenecks from data collection to integration at the national and European level for the operationalisation of EBVs at the European level. Bottlenecks can occur from data collection to data processing (multiple integration levels) and from storage to data sharing. See main text for a full description of bottlenecks and their corresponding acronyms.

To this end, we developed a qualitative framework to categorize and describe the bottlenecks hindering the operationalisation of EBVs using in-situ monitoring data from existing biodiversity monitoring programmes in Europe. These bottlenecks can occur at different stages from its data collection, to the data integration at the National Level and European level (Fig. 1). We evaluated the EuropaBON EBV definitions- as outlined in^4,17^- in terms of taxonomic coverage, spatial and temporal resolution, benchmarking them against current European-level biodiversity data flows (Table S1, S2, S3). The assessment was structured around 16 specific criteria, organized into three broad categories: 1) data collection and sampling; 2) modelling, interoperability and IT infrastructure; and 3) data integration. For each criterion, we determined whether it was fully met, partially met, or not met by the existing monitoring programs. Criteria that were only partially met or unmet revealed critical bottlenecks in the data flows, which could hinder the effective operationalisation of EBVs at the European scale. We compiled these findings into detailed fact sheets for each monitoring program and EBV (Table S4 to Table S45). Each fact sheet provides a comprehensive overview of the monitoring program’s activities, an assessment of whether the criteria are fulfilled, an identification of the specific bottlenecks encountered, and—where available—insights into the underlying causes of these bottlenecks. By identifying these bottlenecks, we aim to guide targeted investments in improving biodiversity data flows. Addressing these challenges will enhance the reliability and relevance of monitoring efforts, ensuring they provide accurate, up-to-date information on the status and trends of biodiversity—ultimately supporting evidence-based conservation actions.

## Results

### Coverage of Essential Biodiversity Variables across European monitoring programmes

We were able to retrieve detailed information about bottlenecks in data flows for a total of 17 monitoring programmes (Table S1) that could potentially provide 35 EBVs (Table S2): 14 freshwater EBVs (Table S4 to Table S17), 4 marine EBVs (Table S18 to Table S26), and 17 terrestrial EBVs (Table S27 to Table S45). The 17 European coordinated monitoring programmes evaluated here were (see Table S1): IWC, WISE (including WISE-2, WISE-6), NA2RE, EMMA2, EASIN, EBP, EURING, DATRAS (ICES), OSPAR, HELCOM, EBBA2, PECBMS, EPB, eBMS, EVA, LUCAS, OPERA/ENRAM/GLoBAM, and the future EU PoMS, which is not a monitoring initiative in place yet. Altogether, these monitoring programmes collect data that could contribute to the generation of EBVs within the classes “species populations” (62%) and “community composition” (21%), while the other EBV classes were scarcely represented. For example, we could not find any monitoring programme coordinated at the European scale that collects data to operationalise EBVs within the class genetic composition in any of the three realms; only five monitoring programmes collect data that could contribute to the operationalisation of EBVs within the classes species traits (i.e. EBP, EURING for freshwater and marine bird phenology related EBVs), ecosystem’s structure (i.e. EVA), or function (i.e. WISE-6 for freshwater).

### Bottlenecks in biodiversity data flows at the European level

**Fig. 2:**
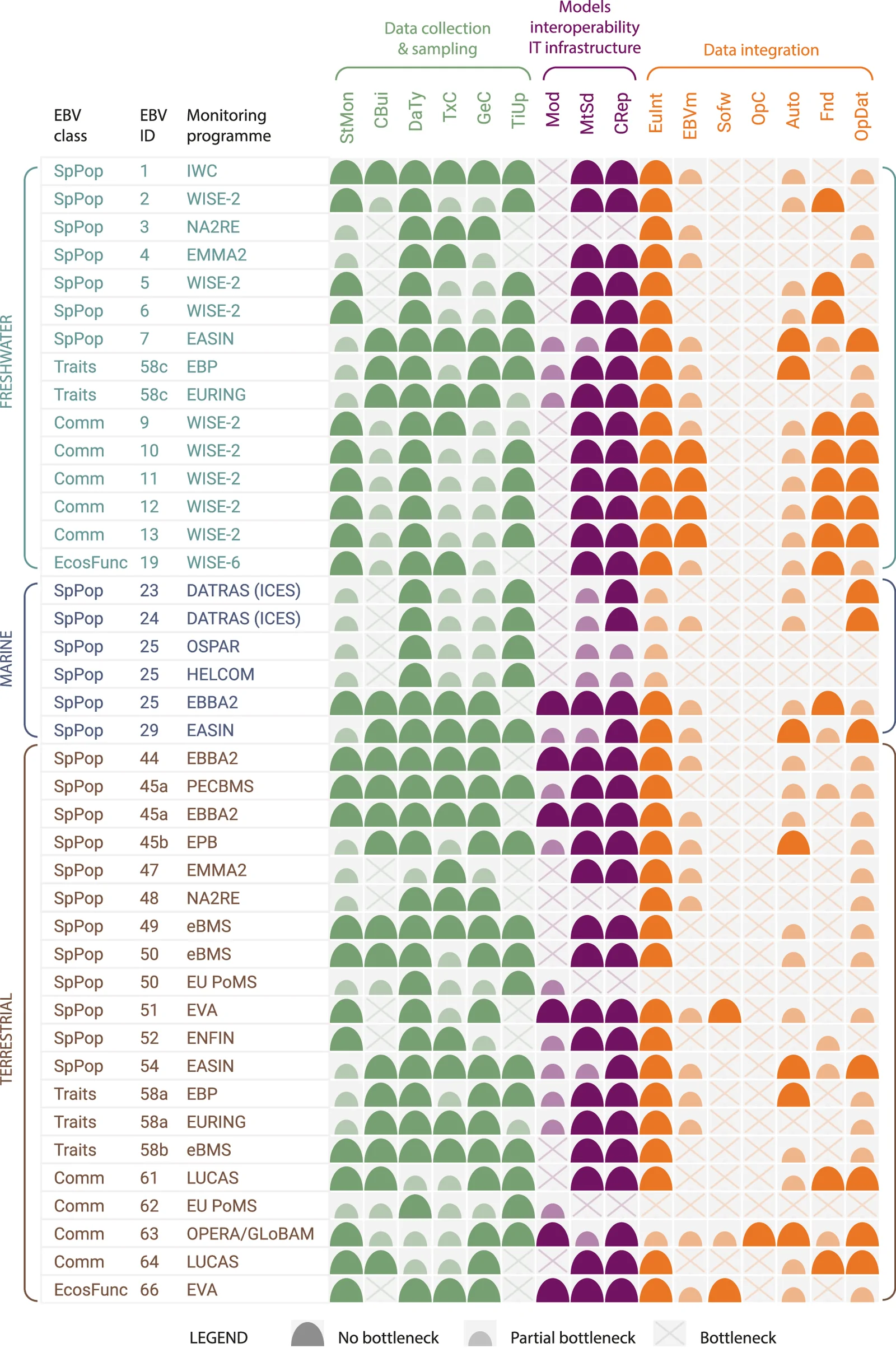
EBV Bottlenecks in European biodiversity monitoring programmes: Data Collection, Models, and Integration. Summary of bottlenecks found across the 35 EBVs evaluated from 17 monitoring programmes, identified in the table through the EBV ID (following^4,17^) and grouped by EBV class (SpPop - Species populations, Traits - Species traits, Comm - Community composition, EcosFunc - Ecosystem function, and EcoStruc - Ecosystems structure). The bottlenecks refer to the data flows of the main biodiversity monitoring programmes, whose data could potentially serve to generate each EBV (identified by its acronym “monitoring programme” column). Note that EU PoMs here represent the bottlenecks identified in the EU PoMS proposal^24,25^ from the current pollinators monitoring state, without being a monitoring programme defined yet, as it will be from 2026. See main text (Bottlenecks criteria section 1 for a full description of the bottlenecks criteria evaluated and their acronyms; here, acronyms are shown in columns and classified into three main categories: 1) data collection and sampling; 2) models, interoperability, and IT infrastructure, and 3) data integration.

All European coordinated monitoring programmes evaluated showed bottlenecks (either partial or total) in different aspects of their data flows to produce EBVs according to our framework. While these programs already integrate and process data to generate a set of biodiversity metrics, products or indicators (e.g. species population trends, habitat suitability maps), these differ from the EBV definition in 85.4% of the EBVs evaluated (partial or full bottleneck EBV match criterion; Fig. 2), either in terms of taxonomic resolution (i.e., not all taxa is covered by a given monitoring programme), spatial resolution (i.e., products already in place have generally a broader spatial resolution than described in the EBV definition) or temporal resolution (i.e., the sampling frequency does not match the EBV definition). In 47.1% of the monitoring programmes assessed, the biodiversity metrics generated by the monitoring programmes do not match the EBV definitions at all (full bottlenecks in EBV match criterion; Fig. 2), with only the WISE-2 monitoring programme not presenting bottlenecks to operationalise EBVs on Community composition (see Supplementary Information).

### Interactions between bottlenecks in data flows

We found the highest prevalence of bottlenecks also on other aspects related to data integration, in particular on the criteria open and reproducible Code (OpC) (partial and full bottlenecks in 95.1% of EBVs), User-friendly software (Sofw) (90.2%), Automatization of data streams (Auto) (80.5%), and funding availability (Fnd) (70.7%). We found that raw data was rarely openly available for the EBV generation (Open Data was a bottleneck in 63.4% EBVs), but in some cases, it could be available either upon request, after the agreement of national coordinators and/or the payment of a handling fee (partial bottleneck in 46.3% of the EBVs evaluated in this study).

The use of models to generate spatially-explicit EBVs from monitoring data is not routinely incorporated in the data flows and processing of most of the monitoring programmes assessed (MoD bottleneck in 80.5% of EBVs; Fig. 2). This is the most relevant bottleneck to the broad category Models Interoperability & IT infrastructure, since most of the monitoring programmes evaluated have already centralized repository infrastructure (CRep) (83.3% of monitoring programmes), and Metadata standards (MtSt) (64.7% of monitoring programmes) in place.

Regarding data collection and sampling, around 56% of EBVs could be generated from data collected via in-situ systematic monitoring programmes (StMon criteria). We found bottlenecks in monitoring data for some taxonomic groups, such as zooplankton in freshwater systems, lichens, terrestrial arthropods, or crop pests, for which we could not find any European-wide monitoring initiative that could provide data to generate the corresponding EBVs at the desired spatio-temporal scale (see Table S3 for EBVs not evaluated in this study). For other better-covered groups (e.g., birds, mammals, plants), we found partial bottlenecks due to the lack of complete species coverage targeted by some EBVs (TxC bottleneck in 51.2% of EBVs; Fig. 2). Geographical Coverage (GeC) was also partially incomplete in 56.1% of EBVs. The frequency of data collection matched the requirements to estimate EBVs in 63.4% of cases (Timely Updated criteria – TiUp). Capacity building (CBui) was a bottleneck in about 56.1% of the EBVs.

We found a bottleneck association among criteria (Fisher’s exact test, p < 0.05; Supplementary Data), especially between funding availability (Fnd) and the criteria within the category modelling, interoperability, and IT infrastructure.

## Discussion

The bottlenecks identified in this study—including fragmented and short-term funding, restricted open data access to raw data, limited automation of end-to-end data flows, and mismatches between current monitoring outputs and prioritized EBV definitions—significantly hinder Europe’s ability to generate the spatially explicit, temporally replicated biodiversity information required for meeting reporting obligations under mandates such as the EU Nature Restoration Regulation (Regulation (EU) 2004/1991) and the Birds and Habitats Directives (Directive 2009/147/EC), as well as other international commitments (e.g. Kunming-Montreal Global Biodiversity Framework targets)^3^. Although the 17 European-coordinated monitoring programmes reviewed do not capture all existing local or national monitoring efforts (which are a good first step of integration), they demonstrate that large-scale data integration at the European level is possible and already provide valuable inputs for operationalising EBVs; however, their usefulness is constrained by insufficient taxonomic, spatial, and temporal resolution, as well as major gaps in open data accessibility, secure long-term funding, and capacity building. Most available data from already existing monitoring data flows allows operationalisation of species populations and community composition EBVs, while data flows needed to compute genetic, functional, and structural EBVs at the European scale are rare or missing.

Data integration is the primary bottleneck category hindering the operationalisation of EBVs across all ecosystem realms and EBV classes at the European level. While numerous biodiversity monitoring networks — such as the European Butterfly Monitoring Scheme, the Pan-European Common Bird Monitoring Scheme— successfully collect and integrate data across taxa and countries, the biodiversity metrics or indicators they generate often do not align with the EBV definitions prioritized by EuropaBON stakeholders for tracking biodiversity trends (EBV match criteria; Fig. 2). This misalignment stems from multiple factors, notably the lack of long-term, sustainable funding. Most continent-wide monitoring initiatives (83%, according to the EuropaBON monitoring database) are coordinated by NGOs, research institutions, or both. Many of their major contributions — such as the European Atlas of Breeding Birds and Mammals, the EuroBird Portal, and GloBAM (now included in Aloft data portal) visualization tools — have relied on competitive or short-term funding sources, such as LIFE, Biodiversa + partnership, or private foundations. This fragmented funding landscape is a major obstacle to ensuring long-term programme continuity, expanding geographic coverage, and supporting essential infrastructure, personnel (e.g., taxonomists, modellers, IT experts), volunteer coordination, and automated data processing systems^6,21^ .

Only a few European biodiversity data flows benefit from sustained funding, particularly those tied to mandatory European reporting obligations like the Habitats Directive, WFD, and the Nature Restoration Regulation. For example, the WISE-6 data flow—which is mandatory for Member States—collects data on water quality measurements. These data could be used to operationalize the EBV on harmful freshwater algal blooms. Also linked to the WFD, but with voluntary reporting, the WISE-2 data flow focuses on Ecological Quality Ratio (EQR) values and has the potential to operationalize eight EBVs included in this study (see Table S2). Notably, WISE-2 provides time series data, enabling trend analysis at the community level^22,23^. Another promising case is the forthcoming European Pollinator Monitoring Scheme (EU PoMS)^24,25^, which became legally binding in December 2025. As part of legally mandated monitoring by Member States under the Nature Restoration Regulation (Regulation (EU) 2024/1991), EU PoMS is expected to generate data to allow operationalising a wide range of pollinator-related EBVs, including species abundance, distribution, taxonomic diversity, and aggregated abundance.

The operationalisation of spatially explicit and temporally replicated EBVs at the continental scale requires long-term monitoring programmes that span a broad range of environmental gradients. Equally important is that the data collected, but also the derived products/EBVs, comply with FAIR (Findable, Accessible, Interoperable, and Reusable) and CARE (Authority to Control, Responsibility, and Ethics) principles to ensure transparency, equity, and reusability^26,27^, but also that data governance rights are respected. However, our findings indicate persistent bottlenecks in both data access and automation. Most monitoring programmes do not provide open access to raw data or do so only upon request, largely due to data ownership by volunteer networks and the sensitivity of certain information, such as the locations of threatened species. At the same time, cost-efficient EBV operationalisation also depends on the end-to-end automatization of data streams — from collection to integration and analysis (see, for example^28^). In our assessment, more than half of the initiatives only partially achieve automation (Fig. 2). Tools such as web platforms and mobile apps have facilitated some progress by enabling data collectors and national coordinators to upload data, which can then be processed through a combination of automatic and manual quality checks before being used to generate indicators and EBVs. For instance, the second European Breeding Bird Atlas (EBBA2^29^) developed interactive mapping tools that automatically flag inconsistencies in species counts near national borders. However, automation remains limited at the national level, and comprehensive end-to-end workflows are still rare. A few promising initiatives, such as the EuroBirdPortal and the “IAS Europe” app, developed by EASIN for reporting Invasive Alien Species, or the Aloft data portal^30^ that allows tracking bird movements and migrations using weather radar data, are moving toward more integrated, automated data flows. To overcome these combined challenges of access and automation, recent studies recommend the establishment of national and subnational coordination hubs^18,31^, which could support data collection and standardization, secure sharing, and the scaling of EBV operationalisation across Europe.

Statistical modelling can help fill data gaps by producing continuous, spatially-explicit EBV predictions — even for unsampled areas — and by simulating biodiversity responses to environmental changes (e.g., post-restoration outcomes). However, in several monitoring programmes, models in place to generate indicators are not tailored to quantify the specific EBVs. In some cases, only minor adjustments are needed to adapt models to the operationalisation of EuropaBON EBVs (e.g., changes in the spatial resolution of data), while in others, although suitable models exist in the scientific literature, they are not implemented within existing monitoring data flows. One widely used model in the monitoring programmes evaluated is TRIM (Trends and Indices for Monitoring data), applied in programmes like PECBMS, IWC, and eBMS to estimate species trends. However, TRIM cannot predict species distributions, making it unsuitable for certain EBV types. Moreover, most modelling is conducted using R, an open-source statistical environment that demands advanced expertise and is often inaccessible to non-specialists. Many modelling scripts are also not openly shared (an “open code” or OpC bottleneck), which limits reproducibility and scalability. This poses a significant barrier, particularly since around 70% of integration initiatives in the EuropaBON database are led by non-research organizations, often reliant on volunteers with limited technical training. Nonetheless, efforts to simplify modelling — such as user-friendly tools like RTRIM shell scripts — are emerging and represent promising steps toward broader EBV adoption.

Our assessment highlighted opportunities to strengthen data flows for EBV classes related to genetic composition, ecosystem function, and ecosystem structure. These EBVs represent areas where advancing data availability and integration at the European level could bring substantial benefits^12^. For instance, the operationalisation of genetic composition and diversity EBVs across terrestrial, freshwater, and marine realms is an emerging frontier that will benefit from increasing efforts in systematic global data collection, harmonized genetic measurement protocols, clearer sampling guidelines, and innovative, cost-effective approaches to data processing^12,32,33^. Likewise, the increased availability of remote sensing products and specialized monitoring programmes represents valuable opportunities for the operationalisation of EBVs linked to ecosystem function and structure^4^ (e.g., marine or terrestrial primary productivity, standing and lying deadwood; see Table S3), which are currently underrepresented. All these improvements are key to tracking biodiversity status and trends from genes to ecosystems and meet the goals of the EU Biodiversity Strategy for 2030.

Our assessment also reveals a lack of coordinated European-scale monitoring programmes for several taxonomic groups, including freshwater zooplankton, lichens, terrestrial arthropods, and crop pests. This does not imply a complete absence of data for these groups, but rather that available data are typically limited to local, subnational, or national levels^19^ (but see^12^ for detailed gap analyses at the national level). In fact, there are already good examples of national monitoring networks that are collecting data that would allow operationalising some of these EBVs. For example, the Rothamsted Insect Survey (RIS) (https://insectsurvey.com/) in the UK has been running two trap networks since 1964 and provides information on aphids, larger moths, and many other migrating insects (i.e., collects data suited to generate the EBV “species abundances of terrestrial crop pests”). Lessons learnt from these national programs could serve to upscale EBVs to broader extents. Our evaluation also showed widespread partial or complete bottlenecks in generating marine EBVs, primarily due to limited European-level data integration and harmonization at broader scales, despite many small monitoring programs and initiatives already in place^5^. Marine monitoring data remain fragmented, typically organized at regional levels (e.g., OSPAR, HELCOM), with Southern and Eastern European waters particularly under-monitored. This fragmentation leads to inconsistencies in protocols and indicators, obstructing the production of standardized marine EBVs. Data flows were mainly available for four species population EBVs, while information on key groups like corals, seagrass, macroalgae, and turtles was lacking — likely due to scarce monitoring programmes or their restricted geographic ranges^12^. These findings align with previous assessments^34^, reinforcing the urgent need to apply a framework like the one proposed in this study and designing more coordinated and comprehensive marine monitoring systems across Europe.

Future efforts should prioritize establishing stable funding mechanisms, strengthening coordination hubs, expanding monitoring to underrepresented taxa and ecosystems, and accelerating data flow automation and open sharing of data and models. Ongoing EU projects and initiatives (e.g., MARCOBOLO, DIVERSEA, OBAMA-NEXT, OBSGESSION, B-Cubed, BMD, BioAgora, Biodiversa + ) are actively working on addressing these challenges, particularly on harmonizing marine monitoring, streamlining remote sensing data flows, and mobilizing data and expert knowledge.

Our assessment shows that although Europe hosts a wide range of coordinated biodiversity monitoring programmes, substantial bottlenecks—such as fragmented and short-term funding, limited data integration and automation of end-to-end data flows, restricted open access to raw data and insufficient alignment between monitoring outputs and EBV definitions—continue to hamper the systematic operationalisation of EBVs. Together, these challenges underscore the need for targeted structural investments, including those envisioned through the proposed European Biodiversity Observation Coordination Centre (EBOCC), a permanent hub to strengthen transnational data flows, harmonize standards, and provide technical and capacity-building support to Member States^4,18^.

Resolving the most critical bottleneck - financial uncertainty - requires securing stable, multi-source funding through EU–national partnerships to ensure long-term continuity of monitoring programmes to train and retain specialized personnel, including taxonomists, modellers, and data engineers^4^. Complementary actions should mandate FAIR-compliant open data sharing that also follows CARE principles, to acknowledge and respect the rights of local communities who steward the land and accelerate the adoption of automated, end-to-end data workflows capable of transforming raw observations into policy-ready insights^4,5,35^ by leveraging novel technologies, such as AI-enabled sensors, remote sensing, and eDNA approaches, among others^36–39^. There is also a need to better embed the operationalisation of EBVs into policy processes—such as the EU regulatory reporting cycles of the Birds and Habitats Directives—as current gaps in the transnational harmonization of monitoring protocols and standards lead to differences in the quantity and quality of the data underlying the trends and conclusions derived from these reports^5,6,14,28,40^. This requires establishing national biodiversity monitoring coordination centres that set standards for data collection and harmonization, and ease data flows and integration at the European scale^35,40^. By reinforcing existing programmes, increasing harmonization efforts, strategically filling current gaps, and making the most of already existing integrated biodiversity dataflows (including integration of data from non-harmonized national and local monitoring efforts), Europe can advance toward a more integrated, FAIR and CARE compliant, and sustainable biodiversity monitoring system, ultimately enabling EBVs to fulfil their intended role as the essential intermediate abstraction layers that connect heterogeneous primary biodiversity data with the high-level indicators required to track progress toward national, regional, and global conservation targets^3,8,41,42^.

## Methods

We searched available information on existing monitoring programmes coordinated at the European scale (“monitoring programmes” from here on) that could support the generation of the EBVs defined by EuropaBON^4,17^ with a specific focus on traditional in-situ biodiversity monitoring data collection (see Tables S1, S2). We benefited from the exchanges with stakeholders during workshops^4–6^ and from previous research efforts in this direction (e.g. the EUMON data set^19^) to identify key integrative monitoring efforts. We then consulted data repositories and informally interviewed/contacted coordinators and stakeholders involved in those monitoring programmes to reconstruct data flows and identify potential bottlenecks across different levels of data integration (e.g., local, subnational, national, European-wide) (see Fig. 1). For example, for the EBV “species distributions of marine fishes”, we based our analyses on the assessment report on the current state of Biodiversity Monitoring in the European Union and adjacent marine waters and its corresponding database^34^. For each of these European-coordinated monitoring programmes, information on biodiversity data collection (e.g., target taxa, geographic and temporal coverage, data type) and how these are harmonized and integrated at different levels (e.g., aggregation across species to generate indicator trends) was compiled in a dataset that is openly available at https://monitoring.europabon.org/monitoring/biodiversity_data/list/^43^. The information provided by experts and coordinators of monitoring programs allowed us to assess the operationalisation potential of each EBV in the form of 35 fact sheets in Supplementary Information (see tables S4 to S45).

We classified the bottlenecks that emerged during the analyses into 16 distinctive criteria. These criteria were agreed upon among EuropaBON partners after many discussions with stakeholders during workshops^5,6^ and also based on previous EBV reference studies addressing the operationalization of EBVs^7,9,35^. These criteria were grouped into three main categories: 1) data collection and sampling, 2) modelling, interoperability, IT infrastructure, and 3) data integration.

Bottlenecks related to data collection and sampling refer to different aspects linked to the collection of in-situ biodiversity data itself that allow or hamper ulterior data harmonization and integration at different levels (Fig. 1). The criteria evaluated in this bottleneck category were:

### Standard monitoring (StMn)

This criterion assesses whether systematic monitoring programmes rely on the same sampling protocols and principles for data collection. Uniform protocols minimize potential data biases inherent of the unstructured and opportunistic data (e.g. observation and spatial bias, reporting preferences and errors^44^), enabling subsequent data harmonization and integration. Moreover, many systematic monitoring programmes are carried out over broad geographic areas and long-time spans allowing to estimate trends of change and eventually understanding the drivers behind those changes (data from long-term monitoring programmes can be especially valuable to fit robust models and predict EBV values over space and time when compared with other biodiversity data (e.g., opportunistic records)^45^ (see the “Models” criterion below); therefore, the absence of standardised monitoring programmes is interpreted as a bottleneck for data integration and EBV operationalisation. When a monitoring programme collected both standardised and unstructured data, this was understood as a partial bottleneck.

### Capacity building (CBui)

This criterion assesses whether a given biodiversity monitoring network has successfully demonstrated the ability to strengthen, create, adapt, and maintain biodiversity monitoring capacity and/or data processing and integration over time. Activities such as training volunteers and adding new sites to the monitoring programme are key indicators. A lack of demonstrated capacity building and training within the monitoring network under evaluation is considered a full bottleneck. The existence of capacity building initiatives only on specific levels of the data flows (e.g. either data collection or data processing) or only in some of the integration levels (e.g. at national level but not at the local level) was considered a partial bottleneck.

### Data type (DaTy)

This evaluates the type of data collected by the monitoring programme (e.g., presence-only, presence-absence, abundance estimates, genetic diversity) and whether it aligns with the needs for operationalising a specific EBV. If the data collected does not facilitate the operationalisation of the EBV, it is considered a bottleneck. For instance, if the programme only gathers species occurrence data (presence/absence records), it cannot produce EBVs on species abundances, which require count data. A bottleneck in this criterion can also reflect a mismatch between the spatial resolution of the raw data and the EuropaBON EBV definition.

### Taxonomic completeness (TxC)

EBVs are defined for specific biological entities or groups of entities (e.g., species or ecosystem types). This criterion assesses whether the monitoring programme covers all taxa defined within the EBV, the taxonomic coverage is incomplete (missing species; partial bottleneck), or whether there is no data at all on a given taxonomic group (full bottleneck).

### Geographic completeness (GeC)

This criterion evaluates the European coverage of the biodiversity monitoring programme, considering the number of participating countries. The programme is deemed complete when all European countries collect data for that specific programme. If certain areas of Europe are not included in the monitoring programme, it is regarded as a partial bottleneck.

### Timely Update (TiUp)

Since EBVs are intended to track the progress of various European environmental policies, this criterion examines whether the frequency of data collection matches or exceeds the temporal resolution that was defined by stakeholders for that specific EBV. If the frequency of current data collection efforts does not match EBV specifications, this represents a bottleneck to operationalise EBVs in support of environmental policies reporting. Some EuropaBON EBVs were defined at two spatial or temporal resolutions (e.g., every 3 and 6 years): when the data available allows only matching one of these time frames, we considered there is a partial bottleneck.

The second group includes bottlenecks related to modelling, interoperability, and IT infrastructure. These are the following ones:

### Models (Mod)

Here, we assess whether a monitoring programme uses statistical models to extrapolate data from biodiversity observations and estimate EBVs or other indicators across Europe (e.g. to compute population trends), and whether these models could be used to operationalise the targeted EBVs. If the monitoring programme does not use models to develop spatially and/ or temporally-explicit datasets, this hampers the generation of EBVs at the European scale and is thus regarded as a bottleneck. If models have been used for such extrapolation only in academic settings, or using a restricted set of taxa but not generally across all species and data available for a given monitoring programme, this was regarded as a partial bottleneck. Note here that the use of models is not needed in all cases to generate EBVs: methods such as spatial aggregation or interpolation (e.g., kriging) might, in some cases, be sufficient to generate EBVs at the desired spatio-temporal scale. We also acknowledge that modelling, ecological inference and prediction may not be the objectives in all monitoring programs. However, statistical models are essential for extrapolating biodiversity metrics from individual sampling units - such as point samples, linear transects, or grid cells - to a continuous space. These models also facilitate the upscaling and downscaling of biodiversity data resolution, thereby enabling the operationalisation of EBVs at continental scale^35^. For example, community-level species distribution models have been used to produce habitat suitability maps of the “potential area of occupancy” of habitats of Conservation Community Interest by the European Habitat Directive 92/43/ECC. These have a spatial resolution of 1-km and span the whole of Europe^46–49^. They are based on a combination of a) data on vegetation records derived from systematic monitoring programmes (the European Vegetation Archive^50^); and b) environmental predictors available at the continental scale (e.g., climatic and land use predictors). Similar approaches have been used to map the distribution of different vegetation forms across Europe^51^.

### Metadata Standards (MdSt)

Adopting metadata standards or data models for data integration enhances the traceability and replicability of data flows. These standards also facilitate the automation of data integration process by setting clear, comparable definitions and reference terms across biodiversity data sets (e.g., concerning spatial and temporal coverage, resolution, measurement units, sampling strategy, and taxonomy). Within this framework, the lack of metadata standards is therefore identified as a bottleneck to operationalising EBVs at the continental scale. If metadata standards are applied inconsistently—such as when standards exist for data reporting to improve handling, integration, and availability, but common standards for data collection are lacking—this is classified as a partial bottleneck.

### Data Repositories (CRep)

Ideally, the EBVs generated from biodiversity monitoring data should be available in the long term (e.g., to allow subsequent time series analyses). This requires a permanent or semi-permanent storage infrastructure^7^. Under this criterion, we evaluated whether the raw and processed data from a specific monitoring programme are stored in a centralized data repository and/or database that adheres to standardized data management protocols. The lack of such a central repository is therefore understood as a bottleneck to operationalising EBVs for existing data flows. If a centralized repository exists but its data are inaccessible, this is classified as a partial bottleneck.

Finally, the *Data integration* group of bottlenecks includes those related to the data integration process:

### European Integration node (EuInt)

Because the focus of this analysis is the operationalisation of EBVs at the European scale, the lack of an integration node for the target taxa at the European level is considered a bottleneck. By European integration node, we refer here to an institution or entity responsible for coordinating and processing a given monitoring data flow and its associated product, indicator, or EBV (frequently, this European node integrates data from national or regional nodes, and not the raw biodiversity data directly).

### EBV match (EBVm)

This criterion evaluates the overall alignment between the EBV definition (following the EuropaBON EBV definitions with regards taxonomic focus, temporal and spatial resolution) and the monitoring programme under assessment. A complete mismatch across all EBV dimensions (taxonomic, spatial, and temporal) is classified as a full bottleneck. Conversely, a partial bottleneck occurs when there is alignment in one or more EBV dimensions, but not all.

### Software (Sofw)

This criterion evaluates whether a given monitoring programme uses a user-friendly software for modelling EBVs that is easy to access and operate by non-scientists (note here this criterion only applies if biodiversity models are routinely in place within the framework of the monitoring programme – see Mod criterion above). Complex modeling approaches requiring specialized technical or statistical expertise are therefore considered a bottleneck to EBV generation in this framework. Intermediate situations (e.g. models are implemented in non-user-friendly software but made available through an easy-to-assess online platform), are considered a partial bottleneck.

### Open and reproducible code (OpC)

This criterion is related to the use of statistical models for extrapolating EBVs across different geographic regions. Currently, there is a growing interest in using open-source software for statistical computing, such as R and Python. These tools offer users flexibility in handling diverse input data types and formats, as well as implementing a wide range of models. While proficiency in programming is required to use these tools effectively, it is increasingly common practice to share code publicly in repositories like GitHub or GitLab. This transparency not only facilitates the use of these software tools but also enhances the reproducibility of analyses. In our framework, we identified that programmes integrating complex modelling approaches without publicly available programming code create a bottleneck in the data flow necessary for operationalising EBVs.

### Automated data streams (Auto)

Automated data processing pipelines — from biodiversity collection to integration at multiple levels — are crucial for accelerating the operationalisation of EBVs and reducing errors in data handling and processing. The rapid development of new Apps and data pipelines is already facilitating automation in biodiversity monitoring. For instance, the EuroBirdPortal aggregates daily observations of bird species across Europe, providing distribution maps at a 30 ×30 km resolution. This is achieved through automatic data processing, harmonization, and error correction of bird species lists collected by both professional and amateur birdwatchers and submitted through apps to data portals such as the national nodes of ornitho, liveatlas.nl, and eBird. Other examples of automated biodiversity monitoring pipelines include those to detect bird migration from a network of weather radar data^30^, such as e those underpinning the flysafe website – a service used to obtain bird migration forecasts for aviation safety^52^.

### Funding (Fnd)

Financial security is crucial for the long-term generation and maintenance of EBVs. Funding supports coordination and integration tasks, sustains data repositories, and enables the development of tools such as Apps that facilitate data collection and integration. A lack of long-term financial security for monitoring programmes represents a bottleneck to generating EBVs. Funding secured in the mid-term, but strongly dependent on renewal of project funding, was considered a partial bottleneck.

### Open Data (OpDat)

The availability of open data is a vital requirement for operationalising EBVs. Raw data and biodiversity datasets often fail to meet the FAIR principles^26^ – being findable, accessible, interoperable, and reusable. This limitation hinders the data flows necessary for operationalising EBVs. This criterion evaluates both the availability of raw data and the conditions under which it can be accessed. If data is neither open nor accessible, it is considered a full bottleneck. If data is accessible only upon request - e.g., pending the approval from data providers – it was considered a partial bottleneck.

**Fig. 3:**
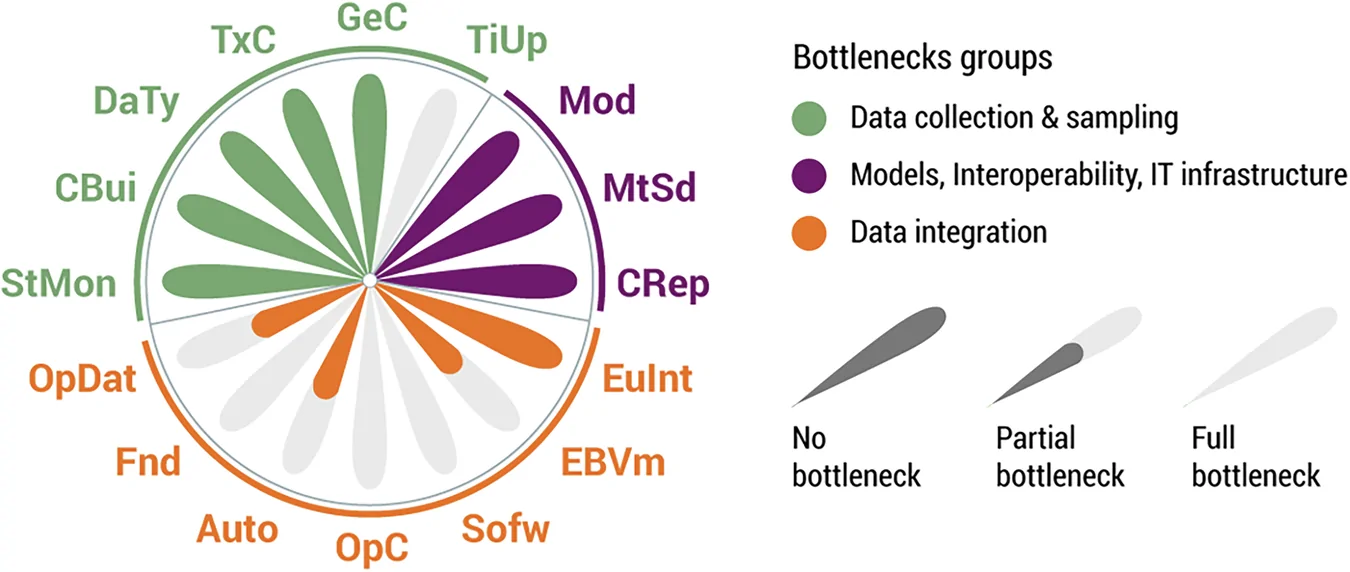
Visual representation of the bottlenecks criteria evaluated for each EBV, showcasing the example of the EBV terrestrial birds and the monitoring scheme EBBA2. Each criterion is represented as a petal in the flower. A fully coloured petal indicates a criterion is fulfilled by a monitoring programme or a particular EBV (i.e., no bottlenecks were identified for that criterion). Petals coloured in light grey indicate the presence of bottlenecks, either full or partial (full or half-coloured grey petal, respectively). Acronyms of the 16 criteria are detailed in the main text (Bottlenecks criteria section). See from Table S4 to Table S45 the detailed description of each EBV and its visual representation for each bottleneck criterion evaluated.

To ease the interpretation of EBV bottlenecks and integration of results across monitoring programmes, we visually represented the 16 bottlenecks criteria as petals in a flower plot (Fig. 3). This visual support also facilitated the discussion with coordinators and stakeholders involved in the different monitoring programmes assessed against the EuropaBON EBV list. As an example of how each flower plot was completed, we present the evaluation of the EBV “Species distributions of terrestrial birds”. We identified the Second European Breeding Bird Atlas (EBBA2)^29^ as a potential monitoring scheme to produce this EBV (Fig. 3). EBBA2 is the most recent and comprehensive European-wide integration initiative mapping the species distributions of all European terrestrial birds. The EBBA2 is coordinated at the EU level, and has mapped the distribution, abundance and breeding likelihood of 596 breeding birds (all native and non-native breeding species) across Europe at the 50 ×50 km grid resolution. Data were collected from multiple sources at the national level and then integrated at the European level. Moreover, species distribution maps at 10 km resolution (probability of occurrence) were developed for 222 species using statistical modelling. When looking at the categories integrated in Data collection and sampling, we can see that the monitoring followed standardised protocol (StMon), contributed capacity building in different countries (CBui), all EU countries are considered (GeoC), all European birds are considered (TxC), and the program produces the same type of data as required by the EBV definition (DaTy). We can only identify a bottleneck in the category “Timely updated” (TiUp), since it can be difficult to replicate the sampling intensity and effort dedicated to EBBA2 with a frequency lower than 20-30 years for all species (the required temporal resolution unit for this EBV is 3 or 6 years). Regarding Models, Interoperability and IT infrastructure group, there are no bottlenecks to report in this regard. The EBBA2 used models to map the distribution of bird species (probability of occurrence), designed metadata standards for data reporting and aggregation, and developed tools to harmonize and centralize data at the European level. Last, regarding Data integration, we can identify more bottlenecks. There is a partial bottleneck in matching the definition of the EBV (EBVm), since only 222 species were modelled at 10 km resolution and none at a finer resolution, despite that EBBA2 has already generated continuous predictions of the distributions of all birds across Europe. The 10 ×10 km grid species distribution models generated by the project were fit using R (a priori, a non-user-friendly software), and the code used to fit the models is not openly available (OpC and Sofw bottlenecks). Funding is limited and unsecured (Fnd bottleneck). While EBBA2 has made a huge effort to automate data flows (e.g., data aggregation at both the national and European levels have been automated using MapViewers), the automatization of data flows from sampling plots to European coordinators was lacking (Auto partial bottleneck). Last, all the data generated in EBBA2 is available through open access or upon request that should be approved by an EBCC committee, and data handling fees can be charged (OpDat partial bottleneck) (see Fig. 3, and Table S27 for more details on the definition of this EBV and further information on the analysis of EBBA2 for this EBV).

Finally, we synthesized the retrieved data on bottlenecks across the six broad EBV classes (genetic composition, species traits, species populations, community composition, ecosystem functioning, and ecosystem structure) and three realms (terrestrial, freshwater, and marine) to identify common challenges in EBV operationalisation. When calculating summary statistics for a given EBV, we included all monitoring programs collecting data potentially suitable for operationalising that EBV. For example, the European Butterfly Monitoring Scheme can potentially contribute to operationalising three EBVs, including 1) butterfly species populations, 2) species populations of priority invertebrates and key pollinators, and 3) species traits, regarding the phenology of the emergence of butterflies. Although this is a single program, it was accounted for three times, one for each corresponding EBV. Associations between bottlenecks among criteria were tested using Fisher’s exact test. This test was chosen over a Chi-squared test because the frequencies in some bottleneck categories (i.e. non-bottleneck, partial-bottleneck, full-bottleneck) were <5. All tests were two-sided.

A detailed assessment for each EBV, monitoring program, their scored bottleneck criteria, and the independence analysis among criteria are provided in the Supplementary Information and Supplementary Data.

## Supplementary information

Supplementary Information

Supplementary Data

## Data Availability

The data that support the findings of this study are available in the file Supplementary Data.
