# Supplementary material for: Europe’s bottlenecks to the operationalisation of Essential Biodiversity Variables for Environmental Policy Support
Source: NPJ Biodivers. 2026 Jul 9;5:37. doi: 10.1038/s44185-026-00147-4 (PMC13675184; doi:10.1038/s44185-026-00147-4)
Supplement: Supplementary file 1 [file 44185_2026_147_MOESM1_ESM.pdf]

Supplementary Information for: **"Europe's bottlenecks to the operationalisation of Essential Biodiversity Variables for Environmental Policy Support"**

Contents

Supplementary Information 1. Data evaluated in the manuscript...1

Supplementary Information 2. EBV fact sheets 7

Supplementary Information 1. Data evaluated in the manuscript

Here you can find the information on the monitoring programs evaluated (Table S1), the list of EBVs considered in the study, and their corresponding monitoring program evaluated (Table S2), and the list of EBVs not considered in the study (Table S3).

Table S1. Monitoring programs evaluated.

**Monitoring program acronym**	**Monitoring Program Description**
IWC	The International Waterbird Census collects abundance data for 554 migratory waterbird populations across the African-Eurasian census on an annual basis.
WISE-2, 6	The Water Information System for Europe for Biology Data (WISE-2) and for Water Quality (WISE-6) are ongoing European monitoring data collection schemes established by the European Environment Agency (EEA).<br><br>WISE-2 dataflow was established to obtain a harmonised flow of biology data reported as Ecological Quality Ratios (EQRs) from all surface water categories: rivers, lakes, transitional and coastal waters.<br>EQRs originated from the Water Framework Directive, but they are not reported under the WFD mandatory reporting by the Member States to the EU.<br>WISE-6 dataflow includes data on nutrients, organic matter, chlorophyll-a, hazardous substances, and general physico-chemical parameters in water, sediment, and biota. This reporting obligation is an Eionet core data flow.
NA2RE	The New Atlas of Amphibians and Reptiles of Europe (NA2RE, 2014) is the most updated Europe-wide coordinated initiative that has attempted to map the distribution of amphibians and reptiles across the continent at 50 x 50 km grid. It generated maps for 218 taxa (73 species of amphibians and 145 of reptiles) updated as of 2014 (no information about temporal dynamics).
EMMA2	The Atlas European Mammals is the first coordinated initiative that has attempted to map the distribution of mammals at 50 x 50 km resolution across Europe (including the freshwater mammals focus of this EBV). The First Atlas of European Mammals was published in 1999 (Mitchell-Jones et al. 1999), and the Second Atlas of European Mammals (EMMA2) is expected to be released in 2026.

EASIN	The European Alien Species Information Network (EASIN) is an integration initiative coordinated at the European level (European Commission Joint Research Centre) that aggregates, integrates, and harmonizes spatio-temporal data for alien species (AS) and Invasive Alien Species (IAS) across Europe.
EBP	The EuroBirdPortal (EBP) combines the data collected by the online bird recording portals operating in Europe to describe the large-scale spatiotemporal patterns of bird distributions and their changes over time.
EURING	EURING coordinates bird ringing in Europe and centralizes the data collected by the different bird ringing schemes operating on the continent to promote and encourage its use for bird management and conservation.
DATRAS (ICES)	The International Council for the Exploitation of the Sea (ICES) holds a database on pelagic and bottom fish trawl surveys collected in the North-east Atlantic and Baltic seas (DATRAS online database).
OSPAR	The OSPAR convention already collects data on breeding seabird colonies (incl. gulls and terns), breeding waterbirds (incl. waders), and wintering and passage water birds (incl. waders) in countries of the Northeast Atlantic, that gets integrated to generate a Marine Bird Abundance indicator. There is currently a joint working group on Marine Birds between OSPAR-HELCOM and the International Council for the Exploration of the Sea (ICES), to enable long-term planning and delivery of significant products across both regions.
HELCOM	HELCOM generates a Marine Breeding Birds abundance indicator integrating data for 6 marine bird species surveyed across the Baltic Sea. There is currently a joint working group on Marine Birds between OSPAR-HELCOM and the International Council for the Exploration of the Sea (ICES), to enable long-term planning and delivery of significant products across both regions.
PECBMS	The Pan-European Common Bird Monitoring Scheme (PECBMS) integrates data on species occurrences and abundance of common birds across Europe following standardized methods. It generates species trends and multi-species trends indicators at the European level and on an annual basis. It also produces indicators for the four main European bioregions.
EBBA2	The Second European Breeding Bird Atlas (EBBA2) (Keller et al. 2020) is the most recent and comprehensive European-wide integration initiative mapping the species distributions of all European terrestrial birds. The EBBA2 has mapped the distribution, abundance, and breeding likelihood of 596 breeding birds (all native and non-native breeding species) across Europe at the 50 x 50 km grid resolution.
eBMS	The European Butterfly Monitoring Scheme (eBMS) collects abundance data for > 312 butterfly species, and in addition collects data on moths, a few bumblebees, and dragonflies. The abundance data collected (counts per transect) are mostly integrated to estimate population trends at the national and European levels (six indicators: grassland, forests, N2000, climate change, widespread species and urban butterflies).
EU PoMS	The European Pollinator Monitoring scheme (EU PoMS) became legally binding on December 6th, 2025. Once implemented by the Member States, it will collect data on the status and trends of pollinator species.<br>The monitoring of pollinator populations by EU PoMS will be key for tracking the goals set in the Nature Restoration Regulation (NRR) on stopping pollinator loss and increasing their populations.
EVA	The European Vegetation Archive (EVA) is an integrative database of vegetation plots across Europe. The purpose of EVA is to establish and maintain a single data repository of vegetation-plot observations. The EVA is an initiative of the Working Group European Vegetation Survey (EVS) of the International Association for Vegetation Science (IAVS), and it is coordinated by a board of members distributed across different European institutions.

ENFIN	The European National Forest Inventory Network (ENFIN) is a pan-European initiative set up in 2003 with the goal of harmonizing data from National Forest Inventories (NFI) across Europe (a network of NFI organizations).
OPERA/ENRAM/GLoBAM	The European Network for the Radar surveillance of Animal Movement (ENRAM) and GloBAM are members of the radar aeroecology community that maintain the community-driven initiative Aloft data portal, which aims to provide access to biological data that comes from European weather radars. The Operational Programme for the Exchange of Weather Radar Information (OPERA) maintains long-term interdisciplinary collaborations with ENRAM, GloBAM, and other networks and projects. Their work resulted in a data license agreement to use the weather data for ecological research, producing the two most comprehensive datasets of aerial biological data in Europe (Desmet et al. 2025). The data can be used to map mass movements of birds and insects in the air across Europe.

Table S2. List of EBVs considered in the study and their corresponding monitoring programs evaluated. Freshwater-related EBVs can be found in tables S4 to S17, marine-related EBVs can be found in tables S18 to S26, and terrestrial-related EBVs can be found in tables S27 to S45.

Realm	Class	EBV taxa	EBVs ID	Monitoring program
Freshwater	Species populations	wetland birds	1	IWC
Freshwater	Species populations	Fishes	2	WISE-2
Freshwater	Species populations	Amphibians and reptiles	3	NA2RE
Freshwater	Species populations	Mammals	4	EMMA2
Freshwater	Species populations	Invertebrates	5	WISE-2
Freshwater	Species populations	Macrophytes	6	WISE-2
Freshwater	Species populations	Invasive alien taxa	7	EASIN
Freshwater	Species traits	Phenology of migration of wetland birds	58c	EBP
Freshwater	Species traits	Phenology of migration of wetland birds	58c	EURING
Freshwater	Community composition	Ecological Quality Ratio (EQR) of phytoplankton in lakes	9	WISE-2
Freshwater	Community composition	EQR of macrophytes	10	WISE-2
Freshwater	Community composition	EQR of phytobentos	11	WISE-2
Freshwater	Community composition	(EQR) of benthic invertebrates	12	WISE-2
Freshwater	Community composition	Fishes	13	WISE-2
Freshwater	Ecosystem function	Harmful algal blooms	19	WISE-6
Marine	Species populations	Fishes	23	DATRAS (ICES)
Marine	Species populations	Commercial fish species and long-distance migratory fishes	24	DATRAS (ICES)
Marine	Species populations	Birds	25	OSPAR
Marine	Species populations	Birds	25	HELCOM
Marine	Species populations	Birds	25	EBBA2
Marine	Species populations	Invasive alien taxa	29	EASIN

Terrestrial	Species populations	Terrestrial birds	44	EBBA2
Terrestrial	Species populations	Common birds	45a	PECBMS
Terrestrial	Species populations	Priority and rare birds	45a	EBBA2
Terrestrial	Species populations	Migratory bird species	45b	EPB
Terrestrial	Species populations	Mammals	47	EMMA2
Terrestrial	Species populations	Reptiles	48	NA2RE
Terrestrial	Species populations	Butterflies	49	eBMS
Terrestrial	Species populations	Priority invertebrates and key pollinators	50	eBMS
Terrestrial	Species populations	Priority invertebrates and key pollinators	50	EU PoMS
Terrestrial	Species populations	Plants	51	EVA
Terrestrial	Species populations	Trees	52	ENFIN
Terrestrial	Species populations	Invasive alien taxa	54	EASIN
Terrestrial	Species traits	Phenology of migration of terrestrial birds	58a	EBP
Terrestrial	Species traits	Phenology of migration of terrestrial birds	58a	EURING
Terrestrial	Species traits	Phenology of the emergence of butterflies	58b	eBMS
Terrestrial	Community composition	Soil microbes	61	LUCAS
Terrestrial	Community composition	Pollinator insects	62	EU PoMS
Terrestrial	Community composition	Migrating birds, bats and insects	63	OPERA/ENRAM/GLoBAM
Terrestrial	Community composition	Functional composition of soil biota	64	LUCAS
Terrestrial	Ecosystem structure	Terrestrial EUNIS habitats	66	EVA

Table S3. EBVs not included in the assessment.

ID	Realm	EBV class	EBV name	Definition
43b	Freshwater	Genetic compositio n	Genetic diversity of selected freshwater taxa	Genetic richness (number of alleles in a population) and genetic evenness (expected proportion of heterozygotes in a population at equilibrium) of taxa.
58c	Freshwater	Species traits	Phenology of migration of wetland birds	The annual timing of arrival and departure of European wetland migratory bird species at breeding, staging and wintering sites over time.
14	Freshwater	Community compositio n	Ecological Quality Ratio (EQR) of freshwater zooplankton	The ecological status of zooplankton in European lakes, measured as Ecological Quality Ratio (EQR).
15	Freshwater	Ecosystem structure	River Connectivity/Free river flow	The length of free-flowing rivers (without barriers) and the natural longitudinal and lateral connectivity of rivers and lakes.
16	Freshwater	Ecosystem structure	Ecosystem distribution of freshwater EUNIS Habitats	The presence/absence or probability of occurrence of freshwater EUNIS habitats in contiguous spatial units (grid cells) over time.
17	Freshwater	Ecosystem structure	Structural complexity of riparian habitats	The vegetation structure, width and length, or topographic heterogeneity of riparian habitats over time, representing the density, cover, variability and three-dimensional arrangement of vegetation and other structural features.

20	Freshwater	Ecosystem function	Freshwater primary productivity	The amount of carbon that is removed by lake habitats (and large rivers) from the atmosphere over time and stored in biomass, roots and sediments.
42c	Marine	Genetic compositio n	Genetic diversity of selected marine taxa	Genetic richness (number of alleles in a population) and genetic evenness (expected proportion of heterozygotes in a population at equilibrium) of taxa.
28	Marine	Species populations	Species distributions of benthic marine invertebrates	The presence/absence or probability of occurrence of benthic invertebrate species form the Habitats Directive in EU's benthic habitats within contiguous spatial units (grid cells) over time
30	Marine	Species traits	Phenology of migration of marine birds and mammals	The annual timing of arrival and departure of European marine migratory bird and mammal species at breeding, staging and wintering sites over time.
31	Marine	Community compositio n	Functional composition of marine phyto/zooplankton (based on traits)	The functional composition and diversity (e.g., based on morphological, physiological or behavioral traits) of marine phyto/zooplankton in EU's marine waters within contiguous spatial units (grid cells) over time
35	Marine	Ecosystem structure	Ecosystem distribution of oyster reef habitats	Presence/absence or probability of occurrence of oyster reef habitats in EU's marine waters within contiguous spatial units (grid cells) over time.
36	Marine	Ecosystem function	Degree of seabed disturbance	The estimated amount of permanent or temporal disturbance of seabed substrate or morphology caused by human activities such as construction, dredging, sand and gravel extraction, deposition of dredged material, shipping and bottom trawling.
37	Marine	Ecosystem function	Harmful marine algal blooms	Distribution, intensity, frequency and position of harmful algal blooms in European coastal waters which occur when cyanobacteria accumulate in water, with the potential to harm the health of humans, plants, and animals
38	Marine	Ecosystem function	Phenology of marine spring phytoplankton bloom	The annual timing and intensity of spring phytoplankton blooms in EU's marine waters over time.
39	Marine	Ecosystem function	Marine primary productivity	Productivity of organic compounds from atmospheric or dissolved carbon dioxide by cyanobacteria, algae and marine plants in EU's marine waters within contiguous spatial units (grid cells) over time.
42a	Terrestrial	Genetic compositio n	Genetic diversity of selected terrestrial taxa	Genetic richness (number of alleles in a population) and genetic evenness (expected proportion of heterozygotes in a population at equilibrium) of taxa.
53	Terrestrial	Species populations	Species distributions of lichens (as indicators of pollution)	The presence/absence or probability of occurrence of ecological quality indicator lichen species within contiguous spatial units (grid cells) across the EU over time.
55a	Terrestrial	Species populations	Species abundances of selected terrestrial disease vectors	The estimated count of individuals of animal vectors within contiguous spatial units (grid cells) across the EU over time.
55b	Terrestrial	Species populations	Species abundances of selected terrestrial crop pests	The estimated count of individuals of crop pests within contiguous spatial units (grid cells) across the EU over time.
56	Terrestrial	Species traits	Phenology of fructification of mushrooms and wild fruits	The annual timing of the fructification of wild mushroom species and wild fruits within contiguous spatial units (grid cells) across the EU over time.
57	Terrestrial	Species traits	Phenology of flowering and leaf senescence	The annual timing of flowering and leaf senescence of European flowering plants and deciduous trees within contiguous spatial units (grid cells) across the EU over time.
59/60	Terrestrial	Community compositio n	Community biomass of selected functional groups of terrestrial	Estimated community biomass of arthropod functional groups.

			arthropods (e.g., predator, decomposer)	
65	Terrestrial	Ecosystem structure	Vertical structure of terrestrial vegetation	The vertical structure of terrestrial vegetation over time, representing vegetation height, cover, density, structural variability and three-dimensional arrangement of vegetation biomass.
67	Terrestrial	Ecosystem structure	Connectivity of terrestrial ecosystem habitat types	The degree of connection of EUNIS habitats within a landscape, in terms of their components, spatial distribution and ecological functions.
69	Terrestrial	Ecosystem function	Terrestrial primary productivity	The amount of CO2 fixed by terrestrial plants through the photosynthetic reduction of CO2 into organic compounds minus the CO2 emitted by autotrophic respiration within contiguous spatial units (grid cells) across the EU over time.
71	Terrestrial	Ecosystem function	Ecosystem disturbance as measured by HANPP	Human Appropriation of Net Primary Production (HANPP) is the proportion of terrestrial NPP consumed directly and indirectly through human land.
73	Terrestrial	Ecosystem function	Standing and lying deadwood	Amount of non-living standing and on the ground woody biomass within contiguous spatial units (grid cells) across the EU forest and other wooded lands over time.

Supplementary Information 2. EBV fact sheets

Here you can find a factsheet for each EBV evaluated. Freshwater EBV fact sheets are from Table S4 to Table S17, Marine EBV fact sheets are from Table S18 to Table S26, and Terrestrial EBV fact sheets are from Table S27 to Table S45.
For each EBV fact sheet, there is a visual representation of the bottleneck criteria evaluated. Each criterion is represented as a petal in the flower. A fully coloured petal indicates a criterion is fulfilled by a monitoring programme or a particular EBV (i.e., no bottlenecks were identified for that criterion). Petals coloured in light grey indicate the presence of bottlenecks, either full or partial (full or half-coloured grey petal, respectively). The acronyms of the 16 criteria are detailed in the main text (see Methods section, and Figure 3).

Freshwater EBV factsheets

Table S4. Freshwater EBV factsheets: Species abundances of selected wetland birds

**EBV: Species abundances of selected wetland birds**	
ID	1
Realm	Freshwater
EBV class	Species populations
EBV name	Species abundances of wetland birds
Step in identification process	Expert workshop
Definition	The (relative) abundance of European rare, priority, and common bird species within contiguous spatial units (grid cells) across the EU over time.
Metric	- Estimated count of individuals in winter<br>- Modeled relative abundance in winter
Spatial resolution unit	1 x 1km – 10 x 10 km (and flyways for migratory)
Temporal resolution unit	Wetlands as defined by The Critical Site Network Tool CSN which is an online resource for the conservation of 312 species of waterbirds and the important sites upon which they depend in Africa and Western Eurasia.
Taxonomic/ ecosystem focus group	Wetland birds (taxonomy based on the HBW and BirdLife Taxonomic Checklist, with focus on those bird species that are officially recognized in the List of birds of the European Union, and wetland affiliation defined as the linkages of species and habitat types to MAES [wetland] ecosystems)

Main European initiatives and description of current bottlenecks

The International Waterbird Census (IWC) collects abundance data for 554 migratory waterbird populations across the African-Eurasian census on an annual basis. It explicitly estimates population trends of waterbirds within the European Birds Directive. In addition, the design of the IWC ensures that all wetland types are represented in the census, as well as wetlands with different conservation status (national, international, unprotected). It is worth mentioning that in addition to the IWC for monitoring waterbirds in their wintering areas, some countries do have other waterbird-specific monitoring schemes for breeding waterbirds and even monthly counts of waterbirds. However, these are not coordinated at international level but are nationally coordinated.

Possible bottlenecks in the generation of this EBV from existing data collected by the IWC network are:

1) Data collection. Waterbird winter censuses are based on long-term counts conducted by citizen scientists on the main European water bodies and are organized at the national and subnational level. In this regard, the type of data collected by this monitoring program matches well the data needed to generate this EBV. Surveys are conducted every year in winter (most of them in January) following standard protocols. Data gets reported at the site level so it could potentially be aggregated or modelled to generate this EBV at the desired spatial resolution. The initiative offers regular training to volunteers and national coordinators contributing to capacity building of the monitoring program.

2) Models, data interoperability and IT infrastructure. The IWC uses models (TRIM) to estimate species trends at the national level following the same methods used by the Pan-European Common Bird Monitoring Scheme (PECBMS). However, no spatially explicit abundance models are generated within the framework of this program (**Mod** bottleneck). Metadata standards are followed to allow harmonization and integration of data from different countries who get stored in a single central repository (global IWC database) managed by the Wetlands International.

3) Data integration. Species counts at the site level get aggregated at the national level and species trends are also estimated at the national scale. Therefore, there is a mismatch between the EBV description, and the products generated by this integration initiative (species trends at site and national level; **EBVm** partial bottleneck). Since this integration initiative does not explicitly model species abundances over space, there is no open code or user-friendly software to report in this regard (**Sofw** and **OpC** bottlenecks**)**, although as with the PECBMS program, it is to be expected that if this initiative were to derive spatially explicit abundance estimates, it would do so by relying on open software and code, as it currently does for estimating species population trends (using the *rtrim* package and TRIM model: TRends and Indices for Monitoring data). Funding is also a limitation: the international coordination, data management and systems are supported by Wetlands International members and the EU LIFE NGO grant; however, in its 2020 annual report, the IWC states "Unfortunately no sustainable funding has been secured for this important work, which depends on the 15,000 volunteers that will be collecting the data in the coming days." (**Fnd** bottleneck). The field monitoring (species counts) is mostly funded by national governments or organizations, but the Waterbird Fund supports counts when these primary funding sources fail. Data flows are partially automated: National coordinators can submit counts directly to the IWC online platform. All counts must be submitted in a standardized Excel format and the system performs several automatic checks. Additional manual checks are also undertaken regularly by Wetlands International staff. The IWC has no plan to develop a system to collect data directly from counters at sites because (a) IWC depends on the network of national coordinators and their teams to perform essential data quality control and coordination for the data collected in their countries and (b) there are already many different apps available to collect waterbird count data in the field. These are widely used by counters, but still the IWC relies on the coordinators to collect and review data reported on these apps to ensure they meet the standard of an IWC count (**Auto** partial bottleneck). Data is available upon request and after the payment of a data handling fee but always requires the authorization of national coordinators, who hold the governance data rights (**OpDat** partial bottleneck).

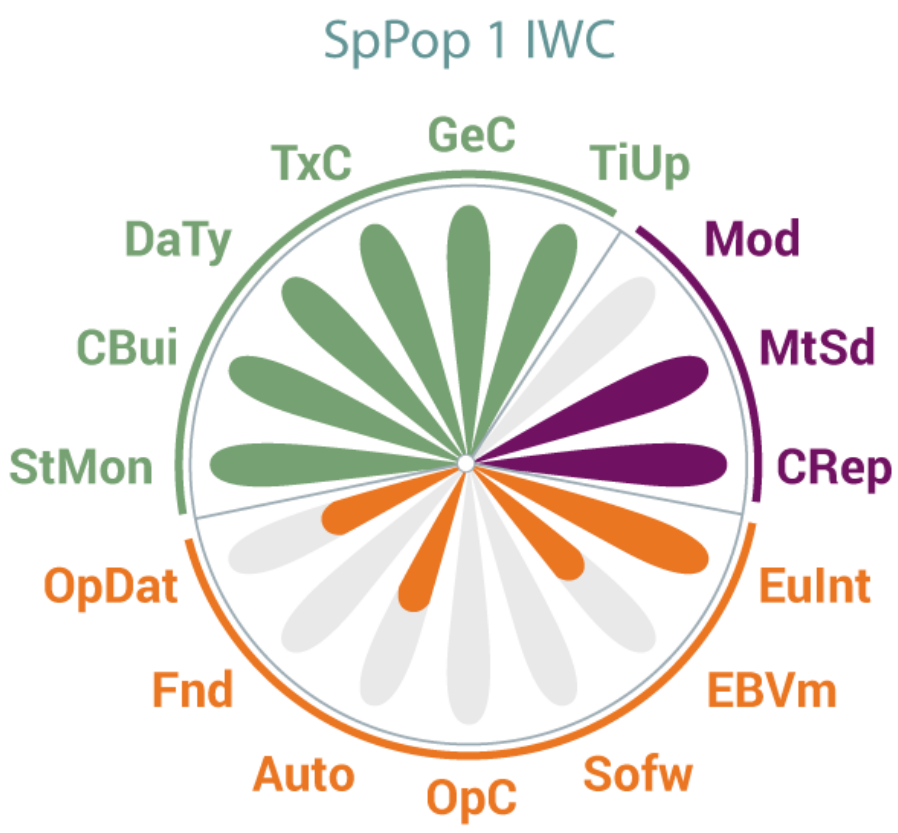

Many passerine bird species with wetland affiliation, as defined as the linkages of species and habitat types to MAES [wetland] ecosystems) are monitored by the Pan European Common Bird Monitoring Scheme (PECBMS). However, the sampling design does not specifically focus on wetlands, surveys are mostly conducted during the breeding period and it is difficult to estimate the coverage of these ecosystems by PECBMS sampling transects. A detailed description of the bottlenecks to generation of spatially-explicit abundance estimates from PECBMS data is detailed in the fact sheet *Species Abundances Terrestrial Birds: COMMON BIRDS.*

Table S5. Freshwater EBV factsheets: Species distributions of freshwater fishes

**EBV: Species distributions of freshwater fishes**	
ID	2
Realm	Freshwater
EBV class	Species populations
EBV name	Species distributions of freshwater fishes
Step in identification process	User & Policy Needs Assessment
Definition	The presence/absence or probability of occurrence of each European freshwater fish species over time.
Metric	- Binary presence/absence<br>- Probability of occurrence
Spatial resolution unit	Lakes and rivers as delineated in ECRINS (European catchments and rivers network system)
Temporal resolution unit	3 or 6 years
Taxonomic/ ecosystem focus group	Freshwater fishes listed in the European Red List of Freshwater Fishes (currently 531 native and described European species)

Main European initiatives and description of current bottlenecks

The **WISE-2** (Water Information System for Europe - Biology data), is an ongoing European monitoring data collection scheme established by the European Environment Agency (EEA) to obtain a harmonized flow of biology monitoring data from all EEA member states reported as Ecological Quality Ratios (EQRs) from all surface water categories: rivers, lakes, transitional, and coastal waters. This data flow is tightly linked to the reporting duties of Member States within the regulations of the Water Framework Directive.

Possible bottlenecks in the generation of this EBV from existing data collected by WISE-2 data relate to:

1) Data collection and sampling. Currently, only 10 European countries report FISH_EQRs to the WISE-2, so there is a gap in the reporting of fish data to the European Commission. This probably translates into a gap in taxonomic coverage given many fish species are endemic to specific river catchments (**GeC, TxC** partial bottlenecks). However, 27 countries collect data on an annual basis on fish species for the Water Framework Directive flows, so probably the geographic and taxonomic monitoring gaps are smaller than expected by WISE-2 data flows. The WISE-2 data flow, as an integration initiative, organizes yearly webinars for all member states with information and Q&A sessions on data reporting, but does not have capacity building on monitoring. If this exists, it is the capacity building of the member states (**CBui** partial bottleneck). While the reported data are EQRs, the underlying raw data collected (composition, abundance, and age structure of fish fauna at the waterbody level) could be potentially used to generate this EBV.
2) Models, interoperability, and IT infrastructure. Currently, there are no models in place to map the distribution of individual freshwater fish species across lakes and rivers within the framework of the WISE-2 flows (**Mod** bottleneck). The EEA centralizes (CDR - Central Data Repository) the EQRs reported by member states and integrates the reporting values to obtain overall EU water quality values representing the specified BQE (biological quality element) and pressure type (e.g. eutrophication). Reporting by Member States follows metadata standards (the structure and contents of the data are described in a Data Dictionary).
3) Data integration. Freshwater fish data reported to the WISE-2 data flow system (EQRs) does not match the definition of this EBV (**EBVm** bottleneck). Countries report FISH_EQRs but these are based on fish records at monitoring sites. Initially, species distributions could be retrieved from raw data/monitored data at the waterbody level and use that information to infer/model species distributions at the European scale. Data flows are not fully automated: the EEA provides empty templates for download from the data dictionary as Excel file and

XML Schema. There is a reporting tool (ReportNet) where to upload these data. An automatic quality control (QC) process is implemented on the Central Data Repository, to check the structure and content of the data file(s) uploaded by each member state. This first step of QC is followed by three more QC steps that involve (2) cross-checking against previously reported data, (3) statistical tools and other algorithms for identifying suspicious values, and (4) expert-based checking and communication with data reporters. Automation of data flows between field data collection and national coordinators yet to be explored (some tests in Norway are underway) (**Auto** partial bottleneck). Since there are not currently models in place to map the distribution of freshwater species, there is no open code or user-friendly software already being used to generate this EBV from WISE-2 data (**OpC** and **SoFw** bottlenecks). Funding must be secured by member states to comply with reporting obligations. While fish EQR ratios at the waterbody level are openly available, the raw data that would serve to generate this EBV are not (species lists collected at each sampling site; **OpDat** bottleneck).

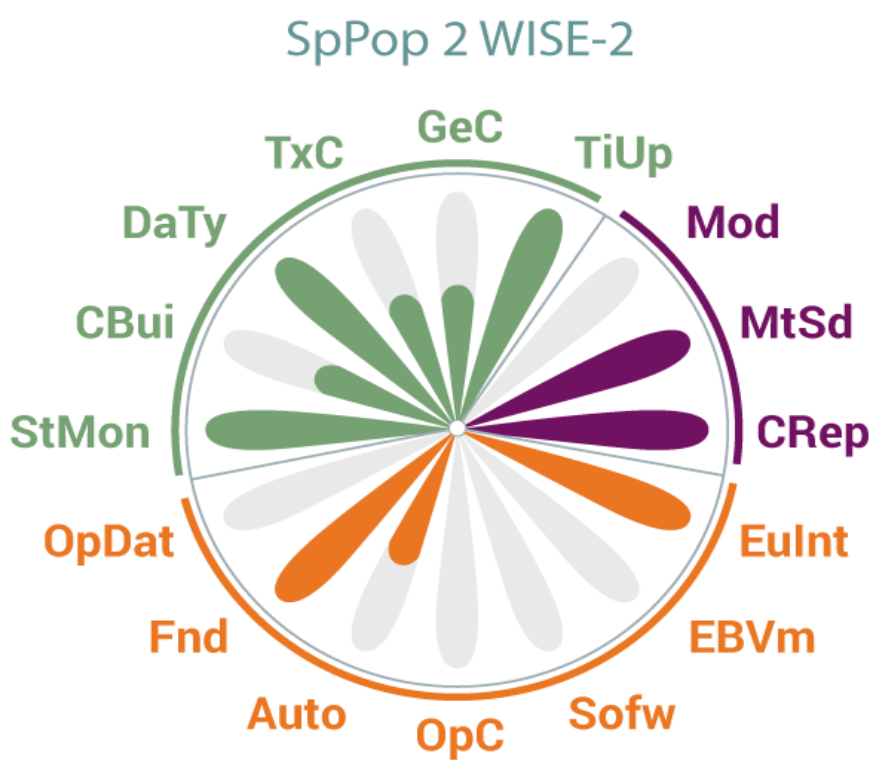

Another integration initiative at the European level retrieving information on freshwater fishes distributions is the IUCN Red List of Freshwater Species. However, most of the time IUCN maps are not linked to a monitoring program (but expert based) and represent a snapshot of the species distributions, so this initiative could not contribute to the quantification of this EBV at the desired spatio-temporal scale. Part of the IUCN evaluation is based on the European Handbook of Freshwater fishes covering 546 native and 33 introduced species.

Table S6. Freshwater EBV factsheets: Species distributions of amphibians and freshwater reptiles

**EBV: Species distributions of amphibians and freshwater reptiles**	
ID	3
Realm	Freshwater
EBV class	Species populations
EBV name	Species distributions of amphibians and freshwater reptiles
Step in identification process	User & Policy Needs Assessment
Definition	The presence/absence or probability of occurrence of each European amphibian and freshwater reptile species within contiguous spatial units (grid cells) over time.
Metric	- Binary presence/absence<br>- Probability of occurrence
Spatial resolution unit	10 × 10 km – 50 × 50 km
Temporal resolution unit	3 or 6 years
Taxonomic/ ecosystem focus group	All European amphibians and freshwater reptiles

Main European initiatives and description of current bottlenecks

The New Atlas of Amphibians and Reptiles of Europe (NA2RE, 2014) is the most updated Europe-wide coordinated initiative that has attempted to map the distribution of amphibians and reptiles across the continent at 50 x 50 km grid. It generated maps for 218 taxa (73 species of amphibians and 145 of reptiles) updated as of 2014 (no information about temporal dynamics).

Possible bottlenecks in the generation of this EBV from data flows in NA2RE relate to:

1) Data collection and sampling. Most of the data used for the Atlas compilation did not come from systematic monitoring programs (**StMon** partial bottleneck). Data sources included: (1) data published (in books or websites) or ongoing national atlases, (2) personal data kindly provided to the *Societas Europaea Herpetologica*, (3) the 1997 European Atlas, and (4) the Global Information Facility (GBIF). While data is available across most European countries (23; i.e., it does fulfil the criteria set to qualify for full Geographic Coverage in this assessment), the data coverage within each country was uneven and very scarce in many cases, with lack of funding and personnel being identified as limiting factors to set up national databases in many countries. The initiative did not have capacity building (if it existed, it was that of the national nodes) (**CBui** bottleneck). The Atlas is a snapshot of the distribution of amphibians and reptiles and therefore, does not match the temporal resolution criteria of this EBV (**TiUp** bottleneck).

2) Models, interoperability, and IT infrastructure. Maps in NA2RE reflect an aggregation of the raw data at 50 x 50 km and there is no modelling involved in predicting species distributions across areas not covered by data (**Mod**). Data from different sources (collected using different standards) were centralized and harmonized for the atlas (**MtSd** bottleneck); the authors of the atlas recognize the challenge of finding better ways to gather species occurrence data across Europe and centralized it (**CRep** bottleneck) (Sillero et al. 2014).

3) Data integration. The maps generated by this integration initiative do not perfectly match the EBV definition (especially the criteria of temporal resolution; **EBVm** partial bottleneck). Data streams were not automated (**Auto** bottleneck). Funding supported the publication of the Atlas, but it is not available in the long-term to generate this EBV with the temporal frequency needed (**Fnd** bottleneck). Since there are no models in place within the framework of this initiative, there is no user-friendly software or open code to report in this regard (**Sofw** and **OpC** bottlenecks). The NA2RE maps are freely available for download, but the raw data are not (**OpDat** partial bottleneck).

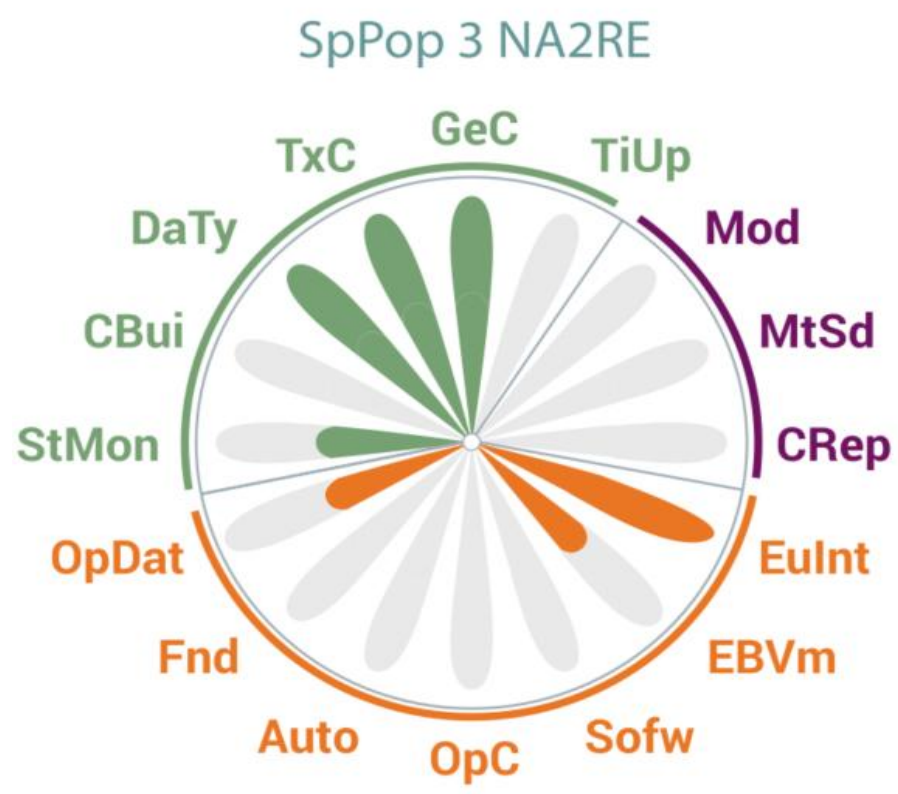

Other integration initiatives at the European level retrieving information on reptiles distributions are the IUCN European Red Lists of Amphibians and Reptiles. However, most of the time IUCN maps are not linked to monitoring programs (but expert based) and represent a snapshot of the species distributions, so this initiative could not contribute to the quantification of this EBV at the desired spatio-temporal scale.

References:

Sillero, N., Campos, J., Bonardi, A., Corti, C., Creemers, R., Crochet, P. A., ... and Vences, M. (2014). Updated distribution and biogeography of amphibians and reptiles of Europe. *Amphibia-reptilia*, 35(1), 1-31.

Table S7. Freshwater EBV factsheets: Species distributions of freshwater mammals

**EBV: Species distributions of freshwater mammals**	
ID	4
Realm	Freshwater
EBV class	Species populations
EBV name	Species distributions of freshwater mammals
Step in identification process	User & Policy Needs Assessment
Definition	The presence/absence or probability of occurrence of each European freshwater mammal species within contiguous spatial units (grid cells) over time.
Metric	- Binary presence/absence<br>- Probability of occurrence
Spatial resolution unit	10 × 10 km – 50 × 50 km
Temporal resolution unit	3 or 6 years
Taxonomic/ ecosystem focus group	Freshwater mammal species listed in the Annex II and Annex IV of the Habitats Directive (*Lutra lutra*, *Galemys pyrenaicus*, *Mustela lutreola*)

Main European initiatives and description of current bottlenecks

The Atlas European Mammals is the first coordinated initiative that has attempted to map the distribution of mammals at 50 x 50 km resolution across Europe (including the freshwater mammals focus of this EBV). The First Atlas of European Mammals was published in 1999 (Mitchell-Jones et al. 1999), but the Second Atlas of European Mammals (EMMA2) is expected to be released in 2026. Possible bottlenecks in the generation of this EBV from existing data collected by EMMA2 data relate to:

1) Data collection and sampling. Most of the data used for the EMMA2 compilation will not come exclusively from systematic monitoring programs (**StMon** partial bottleneck). Data sources include national and regional databases, literature records and data portals. The integration initiative does not have capacity building (if it exists, it depends on national initiatives). As a result of the former points, large gaps of information are expected, particularly in some areas (**GeC** partial bottleneck; 21 countries). The spatial resolution of EMMA2 maps matches the maximum resolution allowed in the definition of this EBV; however, the temporal resolution (frequency) of atlas updates is too low (> 10 years) (**TiUp** bottleneck), therefore the products generated by this integration initiative only partially match the EBV definition (see **EBVm** partial bottleneck in *Data Integration*).

2) Models, interoperability, and IT infrastructure. EMMA2 maps will reflect the confirmed presence of the species aggregated at 50 x 50 km and there is no modelling involved in predicting species distributions across areas not covered by data (**Mod**). Metadata standards are defined (with the intention of following Darwin Core) and data get integrated in a central repository by the European Mammal Foundation.

3) Data integration. The products generated by EMMA2 only partially match the definition of this EBV (there is a mismatch in terms of temporal resolution but also, maps are only available at 50 x 50 km and not at 10 x 10 km; **EBVm** partial bottleneck). As there are no models in place within the framework of this initiative, there is no user-friendly software or open code to report in this regard (**Sofw** and **OpC** bottlenecks). While metadata standards are defined, data flows are not automated (**Auto** bottleneck): records are to be collected into a CSV file or Access database (or similar) and submitted via email to coordinators who harmonize and integrate the data from the different sources. Funding has been secured for the publication of the Atlas but there is no funding to support the long-term continuation of this initiative (**Fnd** bottleneck). The EMMA2 original records will be held in a system that is

accessible to researchers, with appropriate safeguards for sensitive records (rare species, vulnerable habitats or other reasons), though the great majority of records will be freely accessible at a higher resolution than in the Atlas, for example in national or regional atlases (**OpDat** partial bottleneck).

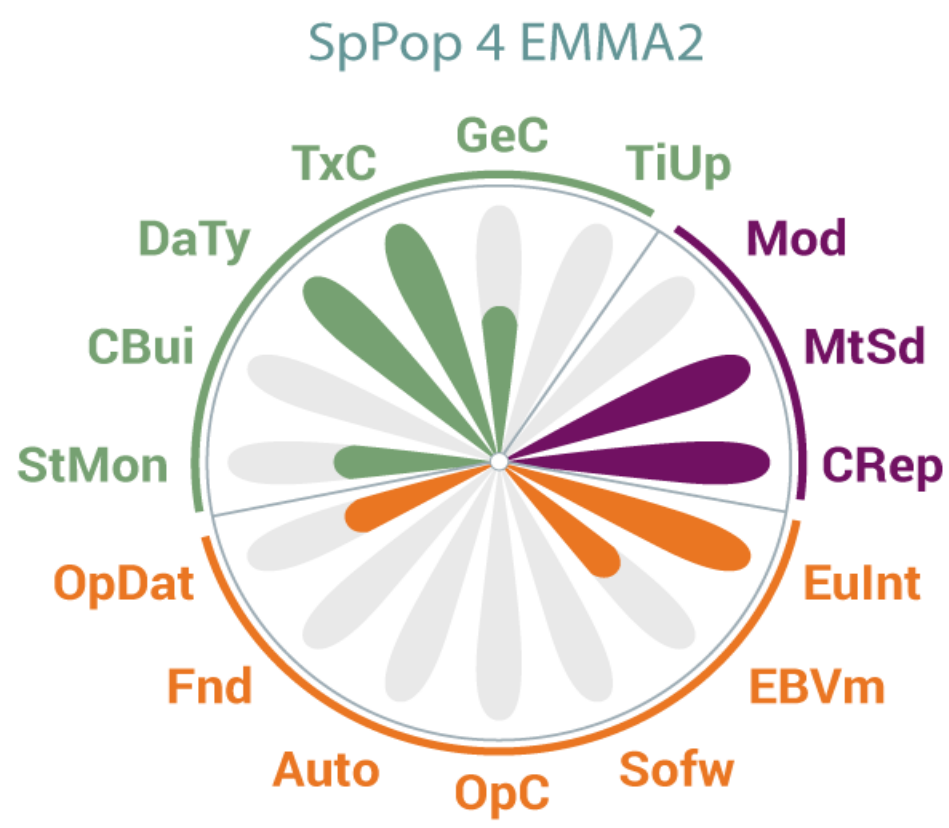

It should be noted here that one of the target species of this EBV (*Galemys pyrenaicus*) is endemic to the Iberian Peninsula and therefore, there is no coordinated initiative at the European level for the monitoring of the species. However, the species is the subject of several regional monitoring programs.

Also, noteworthy here is the development of new integration initiatives such as the European Observatory of Wildlife that could contribute data to the generation of this EBV. The EOW (a project by ENETWILD) aims to enhance collaboration among the stakeholders that monitor, conserve, and manage wildlife in Europe (with a special focus on mammals). It seeks to develop a framework where data will be comparable, interoperable, and openly accessed at the European level, providing guidelines for monitoring mammals in Europe (e.g., protocols to estimate wildlife density), training (e.g., on new tools for data processing and analysis) and compiling and harmonize existing databases on mammals. Data gathered through this portal following standardized monitoring protocols could eventually be used to map the distribution of mammal species across Europe.

References:

Mitchell-Jones et al. (Eds) 1999 The Atlas of European Mammals. *Academic press*, p 484

Table S8. Freshwater EBV factsheets: Species distributions of freshwater invertebrates

**EBV: Species distributions of freshwater invertebrates**	
ID	5
Realm	Freshwater
EBV class	Species populations
EBV name	Species distributions of freshwater invertebrates
Step in identification process	Internal review process
Definition	The presence/absence or probability of occurrence of invertebrate species within lakes and rivers catchments over time.
Metric	- Binary presence/absence<br>- Probability of occurrence
Spatial resolution unit	Lakes and rivers as delineated in ECRINS (European catchments and rivers network system)
Temporal resolution unit	3 or 6 years
Taxonomic/ ecosystem focus group	Freshwater invertebrate species listed in the Habitats Directive Annex II:<br>Dragonflies: *Coenagrion hylas, C. mercuriale, Cordulegaster trinacriae, Gomphus graslinii, Leucorrhina pectoralis, Lindenia tetraphylla, Macromia splendens, Ophiogomphus cecilia, Oxygastra curtisii*<br>Bivalves: *Margaritifera margaritifera* and *Unio crassus*<br>Freshwater invertebrate species listed in the Habitats Directive Annex IV:<br>Dragonflies: *Aeshna viridis, Cordulegaster trinacriae, Gomphus graslinii, Leucorrhina albifrons, L. caudalis, L. pectoralis, Lindenia tetraphylla, Macromia splendens, Ophiogomphus cecilia, Oxygastra curtisii, Stylurus flavipes* and *Sympecma braueri*<br>Bivalves: *Lithophaga lithophaga, Pinna nobilis, Margaritifera auricularia* and *Unio crassus*<br>Pollution-sensitive benthic invertebrates that are monitored for the Water Framework Directive: Mayflies (Ephemeroptera), Stoneflies (Plecoptera), Caddisflies (Trichoptera)

Main European initiatives and description of current bottlenecks

Dragonflies

The only integration initiative at the European level retrieving information on dragonflies distributions are the IUCN European Red Lists of Dragonflies (2010). However, the IUCN maps shown in that report (overall species richness and richness of endemic species) are not linked to a monitoring program (but based on expert consultation and literature review) and represent a snapshot of the species distributions, so this initiative could not contribute to the quantification of this EBV at the desired spatio-temporal scale.

There is no European-wide integration initiative monitoring bivalves that we could report here.

Pollution-sensitive benthic invertebrates

The **WISE-2** (Water Information System for Europe - Biology data), is an ongoing European monitoring data collection scheme established by the European Environment Agency (EEA) to obtain a harmonized flow of biology monitoring data from all EEA member states reported as Ecological Quality Ratios (EQRs) from all surface water categories: rivers, lakes, transitional and coastal waters. This data flow is tightly linked to the reporting duties of Member States within the regulations of the Water Framework Directive.

Possible bottlenecks in the generation of distribution models of pollution-sensitive benthic invertebrates from existing data collected by WISE-2 data relate to:

1) Data collection and sampling. Currently, only 25 European countries report benthic invertebrates in rivers to the WISE-2 data flows thus there is a geographic coverage gap that probably translates into a taxonomic coverage gap (**GeC, TxC** partial bottlenecks). However, 28 countries collect data on an annual basis on freshwater invertebrates on rivers and lakes for the Water Framework Directive flows, so probably the geographic and taxonomic monitoring gaps are smaller than expected by WISE-2 data flows. The WISE-2 data flow, as an integration initiative, organizes yearly webinars for all member states with information and Q&A sessions on data reporting, but does not have capacity building on monitoring. If this exists, it is the capacity building of the member states (**CBui** bottleneck). While the reported data are EQRs, the underlying raw data collected (composition, abundance of benthic invertebrates at the waterbody level) could be potentially used to generate this EBV.

2) Models, interoperability, and IT infrastructure. Currently, there are no models in place to map the distribution of benthic invertebrates across lakes and rivers within the frameworks of the WISE-2 flows (**Mod** bottleneck). The EEA centralizes (CDR - Central Data Repository) the EQRs reported by member states and integrates the reporting values to obtain overall EU water quality values representing the specified BQE (biological quality element) and pressure type (e.g. eutrophication). Reporting by Member States follows metadata standards (the structure and contents of the data are described in a Data Dictionary).

3) Data integration. Benthic invertebrate data reported to the WISE-2 data flow system (EQRs) does not match the definition of this EBV (spatially-explicit species distributions; **EBVm** bottleneck). Countries report EQRs, but these are based on benthic invertebrates' records at monitoring sites. Initially, species distributions could be retrieved from raw data/monitored data at the waterbody level and use that information to infer/model species distributions at the European scale. Since there are not currently models in place to map the distribution of freshwater species, there is no open code or user-friendly software already being used to generate this EBV from WISE-2 data (**OpC** and **SoFw** bottlenecks). Data flows are not fully automated: the EEA provides empty templates for download from the data dictionary as Excel file and XML Schema. There is a reporting tool (ReportNet) where Member States can upload this data. An automatic quality control (QC) process is implemented on the Central Data Repository, to check the structure and content of the data file(s) uploaded by each member state. This first step of QC is followed by three more QC steps that involve: (2) cross-checking against previously reported data, (3) statistical tools and other algorithms for identifying suspicious values, and (4) expert-based checking and communication with data reporters. Automation of data flows between field data collection and national coordinators yet to be explored (some tests in Norway are underway) (**Auto** partial bottleneck). Funding has to be secured by member states to comply with reporting obligations. While benthic invertebrates EQR ratios at the waterbody level are openly available, raw data are not (species lists; **OpDat** bottleneck).

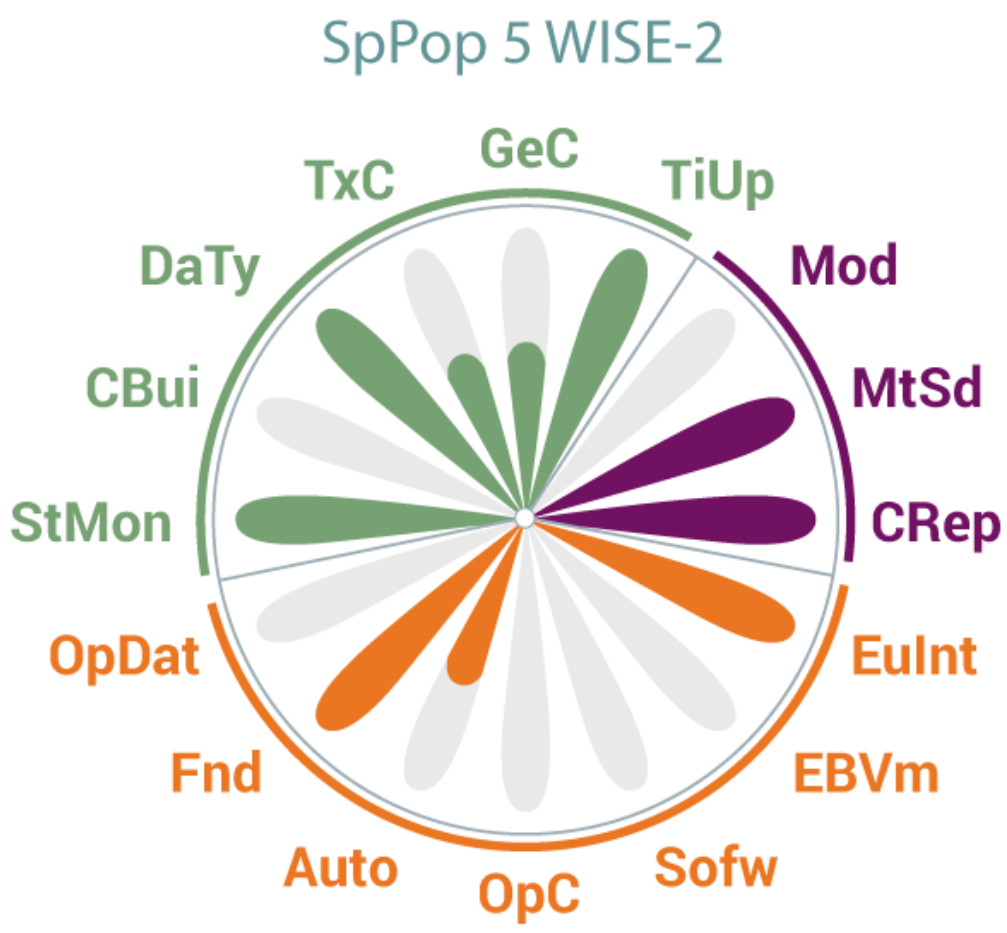

Table S9. Freshwater EBV factsheets: Species distributions of freshwater macrophytes

**EBV: Species distributions of freshwater macrophytes**	
ID	6
Realm	Freshwater
EBV class	Species populations
EBV name	Species distributions of freshwater macrophytes
Step in identification process	Internal review process
Definition	The presence/absence or probability of occurrence of European freshwater macrophyte species within lakes over time.
Metric	- Binary presence/absence<br>- Probability of occurrence
Spatial resolution unit	Lakes as delineated in ECRINS (European catchments and rivers network system)
Temporal resolution unit	3 or 6 years
Taxonomic/ ecosystem focus group	European macrophytes

Main European initiatives and description of current bottlenecks

The **WISE-2** (Water Information System for Europe - Biology data), is an ongoing European monitoring data collection scheme established by the European Environment Agency (EEA) to obtain a harmonized flow of biology monitoring data from all EEA member states reported as Ecological Quality Ratios (EQRs) from all surface water categories: rivers, lakes, transitional and coastal waters. This data flow is tightly linked to the reporting duties of Member States within the regulations of the Water Framework Directive.

Possible bottlenecks in the generation of this EBV from existing data collected by WISE-2 data relate to:

1) Data collection and sampling. Currently, only 15 European countries report macrophytes in lakes to the WISE-2 data flows thus there is a geographic coverage gap that probably translates into a taxonomic coverage gap (**GeC**, **TxC** partial bottlenecks). However, 19 countries collect data on an annual basis on freshwater macrophytes in lakes for the Water Framework Directive flows, so probably the geographic and taxonomic monitoring gaps are smaller than expected by WISE-2 data flows. The WISE-2 data flow, as an integration initiative, organises yearly webinars for all member states with information and Q&A sessions on data reporting, but does not have capacity building on monitoring. If this exists, it is the capacity building of the member states (**CBui** bottleneck). While the reported data are EQRs, the underlying raw data collected (composition - presence/absence and abundance of aquatic flora at the waterbody level) could be potentially used to generate this EBV.

2) Models, interoperability, and IT infrastructure. Currently, there are no models in place to map the distribution of macrophytes across lakes within the frameworks of the WISE-2 flows (**Mod** bottleneck). The EEA centralizes (CDR - Central Data Repository) the EQRs reported by member states and integrates the reporting values to obtain overall EU water quality values representing the specified BQE (biological quality element) and pressure type (e.g., eutrophication). Reporting by Member States follows metadata standards (the structure and contents of the data are described in a Data Dictionary).

3) Data integration. Macrophytes data reported to the WISE-2 data flow system (EQRs) does not match the definition of this EBV (spatially-explicit species distributions; **EBVm** bottleneck). Countries report Macrophytes EQRs in lakes but these are based on macrophyte records at monitoring sites (composition - presence/absence and abundance of aquatic flora). Initially, species distributions could be retrieved from raw data/monitored data at the waterbody level and use that information to infer/model species distributions at the European scale. Since

there are not currently models in place to map the distribution of freshwater species, there is no open code or user-friendly software already being used to generate this EBV from WISE-2 data (**OpC** and **SoFw** bottlenecks). Data flows are not fully automated: the EEA provides empty templates for download from the data dictionary as Excel file and XML Schema. There is a reporting tool (ReportNet) where Member States can upload these data.
An automatic quality control (QC) process is implemented on the Central Data Repository, to check the structure and content of the data file(s) uploaded by each member state. This first step of QC is followed by three more QC steps that involve: (2) cross-checking against previously reported data, (3) statistical tools and other algorithms for identifying suspicious values, and (4) expert-based checking and communication with data reporters. Automation of data flows between field data collection and national coordinators yet to be explored (some tests in Norway are underway) (**Auto** partial bottleneck). Funding has to be secured by member states to comply with reporting obligations. While Macrophytes EQR ratios at the waterbody level are openly available, raw data are not, hampering its access for the development of distribution models for this taxonomic group (**OpDat** bottleneck)

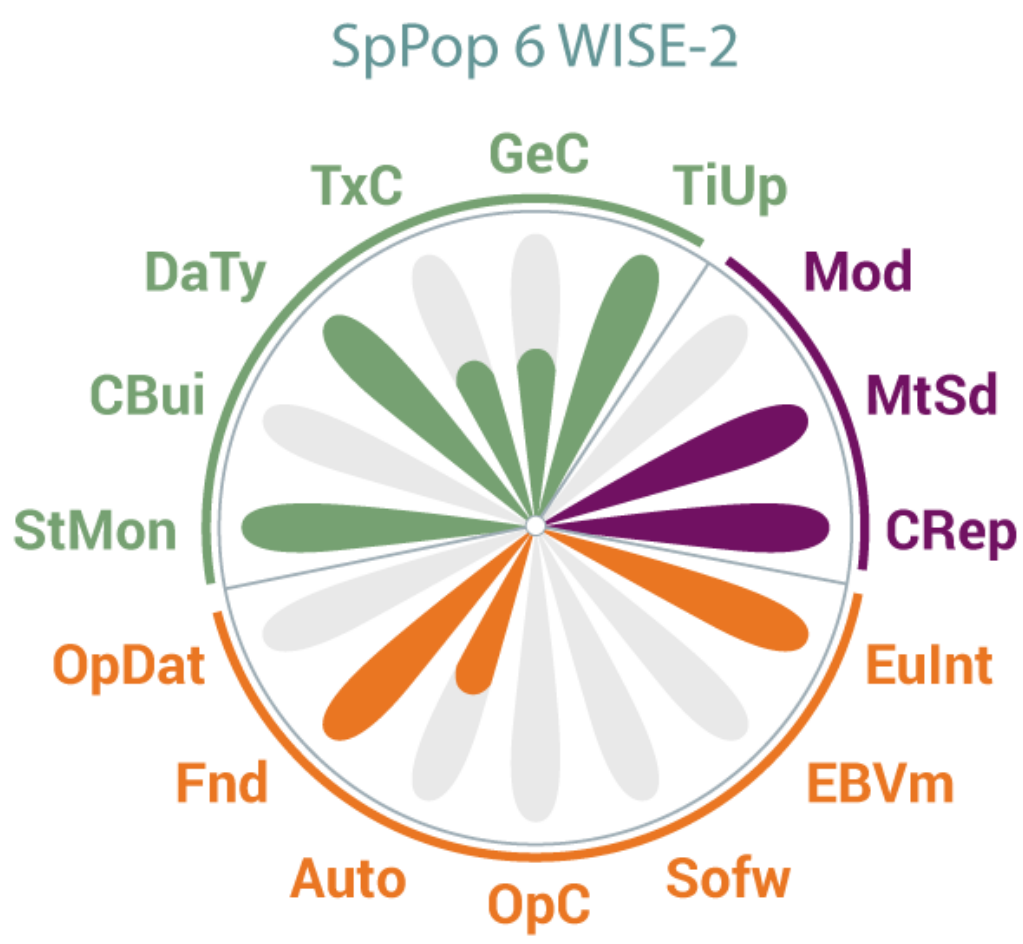

Table S10. Freshwater EBV factsheets: Species distributions of invasive alien freshwater taxa of European concern

**EBV: Species distributions of invasive alien freshwater taxa of European concern**	
ID	7
Realm	Freshwater
EBV class	Species populations
EBV name	Species distributions of invasive alien freshwater taxa of European concern
Step in identification process	User & Policy Needs Assessment
Definition	The presence/absence or probability of occurrence of invasive freshwater species within lakes and river catchments over time.
Metric	- Binary presence/absence<br>- Probability of occurrence
Spatial resolution unit	Lakes and river catchments as delineated in ECRINS (European catchments and rivers network system)
Temporal resolution unit	3 or 6 years
Taxonomic/ ecosystem focus group	Freshwater species specified in the Commission Implementing Regulation 2016/ 1141, 2017/1263, 2019/1262, 2022/1203, 2025/1422. Consolidated list at:https://eur-lex.europa.eu/legal-content/EN/TXT/PDF/?uri=CELEX:02016R1141-20220802&from=EN

Main European initiatives and description of current bottlenecks

The European Alien Species Information Network (EASIN) is an integration initiative coordinated at the European level (European Commission Joint Research Centre) that aggregates, integrates, and harmonizes spatio-temporal data for alien species (AS) and Invasive Alien Species (IAS) across Europe. The species catalogue and geodatabase are regularly updated through literature reviews, data partners inputs, and the official reports by Member States competent authorities. The EASIN Catalogue (29.07.2025) includes 778 freshwater AS, of which 46 are IAS of Union Concern (EU Regulation 1143/2014). The Catalogue's protocol provides information on the data collected for each species. EASIN facilitates the exploration of data and information from existing monitoring networks and programs available from a variety of distributed information sources by providing tools and interoperable web services, compliant with internationally recognized standards.

Possible bottlenecks in the generation of this EBV from existing data collected by EASIN data relate to:

1) Data collection and sampling. EASIN carries out the systematic collection of data on IAS in Europe, does not come exclusively from monitoring programs but from a variety of sources, including literature review, occasional observations and data portals (**StyMon** partial bottleneck); EASIN has demonstrated capacity building through different activities including: i) *ad hoc* assessments, such as the Baseline Distribution of IAS of Union concern; ii) support to the establishment of surveillance systems in compliance with EU AS policy; iii) citizen science programs, (e.g. the development of the smartphone app 'IAS in Europe' freely available and already adopted in the context of several projects and for official monitoring; iv) training to teachers and students (e.g., the MOOC course "Have you seen an alien?"), among others. EASIN collaborates with the Member States competent authorities and national experts to update and validate the datasets relevant in the EU policy context, to fulfil the EU mandate of setting up national monitoring networks for IAS; however, the extent to which this has been implemented is not fully known. For example, in the freshwater realm an evaluation of 2010 showed that the large majority of Member States did not have targeted monitoring for IAS to account for the effects of IAS in the ecological status values reported to the Water Framework Directive (Vandekerkhove and Cardoso 2010; Vandekerkhove et al. 2013; Boon et al. 2020; Magliozzi et al. 2020). Some bottlenecks

regarding data collection may relate to 1) lack of communication between national coordinators & EU funded projects working on IAS in a country (e.g. LIFE projects); 2) at national level people in charge of monitoring AS belong to different admin bodies among which there is little communication and coordination and 3) complex administrative structure in some countries (federal, regions, etc.) and how the environmental competences are distributed among them, making it difficult to harmonise and coordinate data collection tasks and to facilitate data flows and integration. Currently, the EASIN Geodatabase (04.08.2025) contains occurrence records for 14,347 species, across 64different countries (EU & beyond) (including data for the 114 species in the consolidated list of IAS of Union concern - European Union (EU) Regulation 1143/2014). Because the data is georeferenced, it can be used to build species distribution models. The EASIN Geodatabase is updated on average 3 times/ year, but some information sources are updated less regularly, depending on effort for the update and the periodicity of their own updates. Recent EASIN database backend developments data updating will facilitate more regular updates.

2) Models, interoperability, and IT infrastructure. EASIN is not a modelling framework, therefore there is no access to modelling workflows (**Mod** partially bottleneck). A case study using habitat suitability models was carried out for the freshwater invasive species *Elodea nuttalli* using EASIN data across the EU (Steen et al. 2019). There is, however, regular in-house research work on IAS of Union concern to evaluate the range of expansion at both European and national levels, to understand whether niche of the species is at equilibrium in its invaded range or to predict the probability of occurrence of the species in Europe considering bioclimatic variables. EASIN makes data available under the CC-BY licence enabling reuse with attribution to the original sources through web tools for data analysis, open sharing and reporting. The openness of this data is key to redirect surveillance and trigger early warning systems. However, while standards have been defined to harmonize data across source databases, these do not necessarily apply to the underlying data (**MtSd** partial bottleneck).

3) Data integration. Data collected by EASIN are at different resolutions and aggregated at e.g., country, River Basin Districts, 10 x 10 km grid cells, partially match the EBV, as it does not provide the occurrence aggregated at the river catchment level (**EBVm** partial bottleneck). The EASIN Geodatabase follows a protocol to regularly retrieve data from the data partners; data are subsequently transformed and converted through the following steps: validation, cleansing and standardization, geocoding, mapping, application of quality rules and finally loaded on the Geodatabase (Datawarehouse). The EASIN Geodatabase also integrates data from the "IAS Europe'' smartphone App, which promotes the report of sightings of IAS of Union Concern by citizens, and data from the NOTSYS platform. The latter is the official tool for EU Member States to notify the Commission and inform the other Member States as required by Reg. 1143/2014 on IAS. In particular, the tool has been designed to facilitate a timely comprehensive notification of new detections of IAS of Union concern, related eradication measures, and their effectiveness. Funding is a bottleneck to the generation of this EBV: while the European Commission has set up EASIN as the official information system supporting Member States in the implementation of the Regulation (EU) No 1143/2014 , it only co-participate in the expenses of monitoring or eradication actions by national competent authorities, funding from the European Commission for monitoring of IAS is available via existing financial instruments such as LIFE, H2020, Cohesion or Regional Development funds. Funding is warrant in the mid - long - term to maintain the EASIN data infrastructure and the development of new data collection tools (e.g., a web base platform for reporting citizen science data) (**Fnd** partial bottleneck).

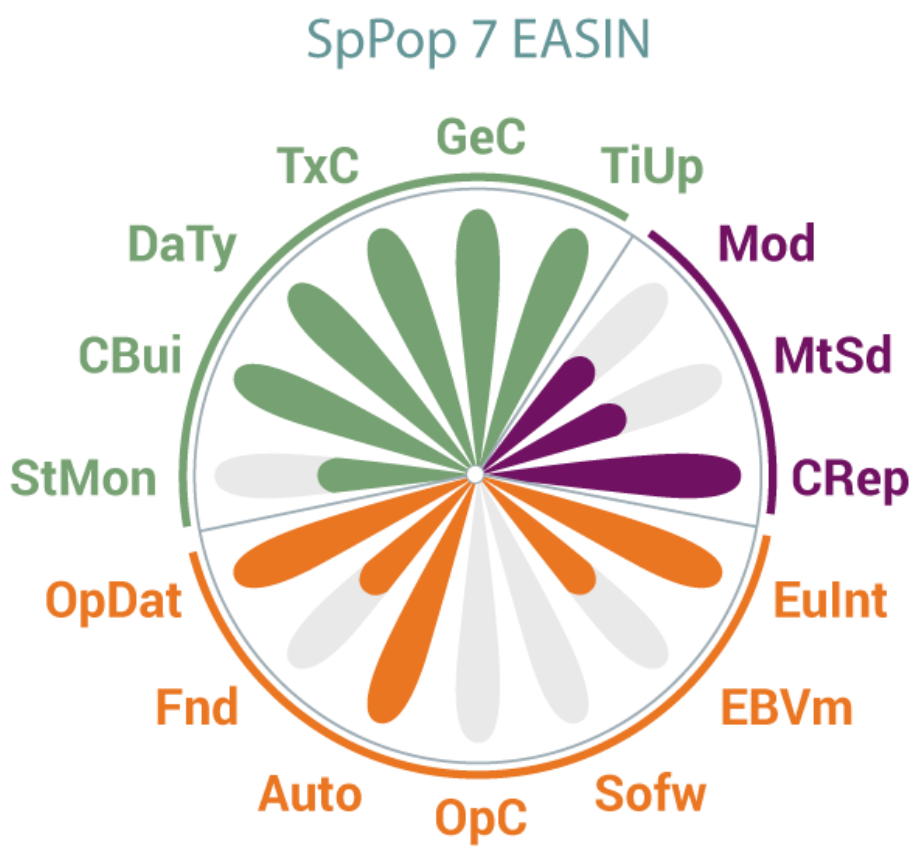

References:

Boon, P. J., Clarke, S. A., and Copp, G. H. (2020). Alien species and the EU Water Framework Directive: A comparative assessment of European approaches. *Biological Invasions*, *22*(4), 1497-1512. URL.

Magliozzi C, Tsiamis K, Vigiak O, Deriu I, Gervasini E, Cardoso AC, 2020. Assessing invasive alien species in European catchments: Distribution and impacts. Science of the Total Environment 732: 138677. https://doi.org/10.1016/j.scitotenv.2020.138677

Steen, B, Cardoso, A.C., Tsiamis, K., Nieto, K.,Engel J. and Gervasini, E. (2019) Modelling hot spot areas for the invasive alien plant Elodea nuttallii in the EU. *Management of Biological Invasions* 10(1): 151–170. URL .

Vandekerkhove, J. and Cardoso, A.C. (2010) Alien species and the Water Framework Directive. Questionnaire results. European Commission - Joint Research Centre. URL.

Vandekerkhove, J., Cardoso, A. C., and Boon, P. J. (2013). Is there a need for a more explicit accounting of invasive alien species under the Water Framework Directive?. *Management of Biological Invasions*, *4*(1), 25. URL.

Table S11. Freshwater EBV factsheets: Phenology of migration of wetland birds

**EBV: Phenology of migration of wetland birds**	
ID	58c
Realm	Freshwater
EBV class	Species traits
EBV name	Phenology of migration of terrestrial birds
Step in identification process	Expert workshop
Definition	The annual timing of arrival and departure of European wetland migratory bird species at breeding, staging, and wintering sites over time.
Metric	Migration phenology metrics such as:<br>- Day of arrival<br>- Day of departure<br>- Length of stay
Spatial resolution unit	10 × 10 km
Temporal resolution unit	1 week
Taxonomic/ ecosystem focus group	Migratory bird species defined as full migrants in the European Red List

Main European initiatives and description of current bottlenecks

There are two European-wide coordinated initiatives that collect data that could potentially be used to generate this EBV: The EuroBirdPortal (EBP) and the EURING.

The **EuroBirdPortal (EBP)** combines the data collected by the online bird recording portals operating in Europe to describe the large-scale spatiotemporal patterns of bird distributions and their changes over time. Online bird portals obtain year-round data from the intensive and widespread activities of birdwatchers. However, data is, essentially, collected using simple standardized protocols (complete lists) or no protocol at all (casual observations). As the EBP collects data from the entire year, the project has the potential to obtain at least some of the metrics required for this EBV (e.g. arrival and departure timing) for several migratory bird species.

Possible bottlenecks in the generation of this EBV from existing data collected by the EBP initiative relate to:

1) Data collection & sampling. The EBP has the potential to collect data on all bird species occurring in Europe but, currently, only the data for 137 species are stored in the EBP central data repository (**TxC** partial bottleneck). Data is collected across all European countries, but the degree of coverage is poorer in the South and, particularly, in the East and South-East. Data (species observations & counts) are gathered from online bird recording portals (e.g., ornitho) and include, essentially, both casual observations and data collected following simple systematic protocols (species lists). Only a small part of the data is collected following standardized monitoring protocols (**StMon** partial bottleneck). Training and capacity building for the network is provided during regular annual meetings or direct support to specific partners. Data is updated daily with information up to the previous day (species lists).

2) Models, data interoperability and IT infrastructure. Despite the EBP/online bird portals data have been used to obtain some of the kind of metrics required to generate this EBV in some specific contexts (e.g. to predict the start of spring migration in the context of delimiting hunting seasons or study changes in migratory phenology; see, for example, Newton et al. 2016), the modelling of phenological traits has not been generalized nor automated within the framework of this initiative (**Mod** partial bottleneck). The EBP follows metadata standards to ensure data harmonization among its different data sources. The EBP data is harmonized, managed, and stored in a central repository curated by the European Bird Census Council.

3) Data Integration. The maps featured in the EBP viewer (www.eurobordportal.org) represent aggregated observations at the weekly basis and at 30 x 30 km resolution (**EBVm** partial bottleneck); however, in the EBP central data repository the casual data are aggregated at 10 x 10 km and date and the lists are in raw format (i.e. not aggregated) with date/timing and location given as precise location or at 10 x 10 km; therefore, the data available at the EBP repository would allow the generation of this EBV at the desired spatio-temporal resolution. Although this integration initiative can produce metrics of the kind required for this EBV, there is no open code nor user-friendly software yet to report in this regard as models are not routinely used to generate phenology products from EPB data yet (**Sofw** and **OpC** bottlenecks). Data streams are automated: the local online portals collect most of their data through mobile apps in near-real time or shortly after it has been recorded in the field; data collected by the data portals is then automatically transferred to the EBP daily. The EBP main developments have been possible thanks to the support of the LIFE programme, but funding stability in the mid- long- term is not warranted (**Fnd** bottleneck). EBP data is available upon request and subject to agreement by National coordinators who hold the ownership of data (**OpDat** partial bottleneck), but just one centralized data request should be done as data is already centralized in the EBP databank (authorizations by national owners are coordinated by EBP).

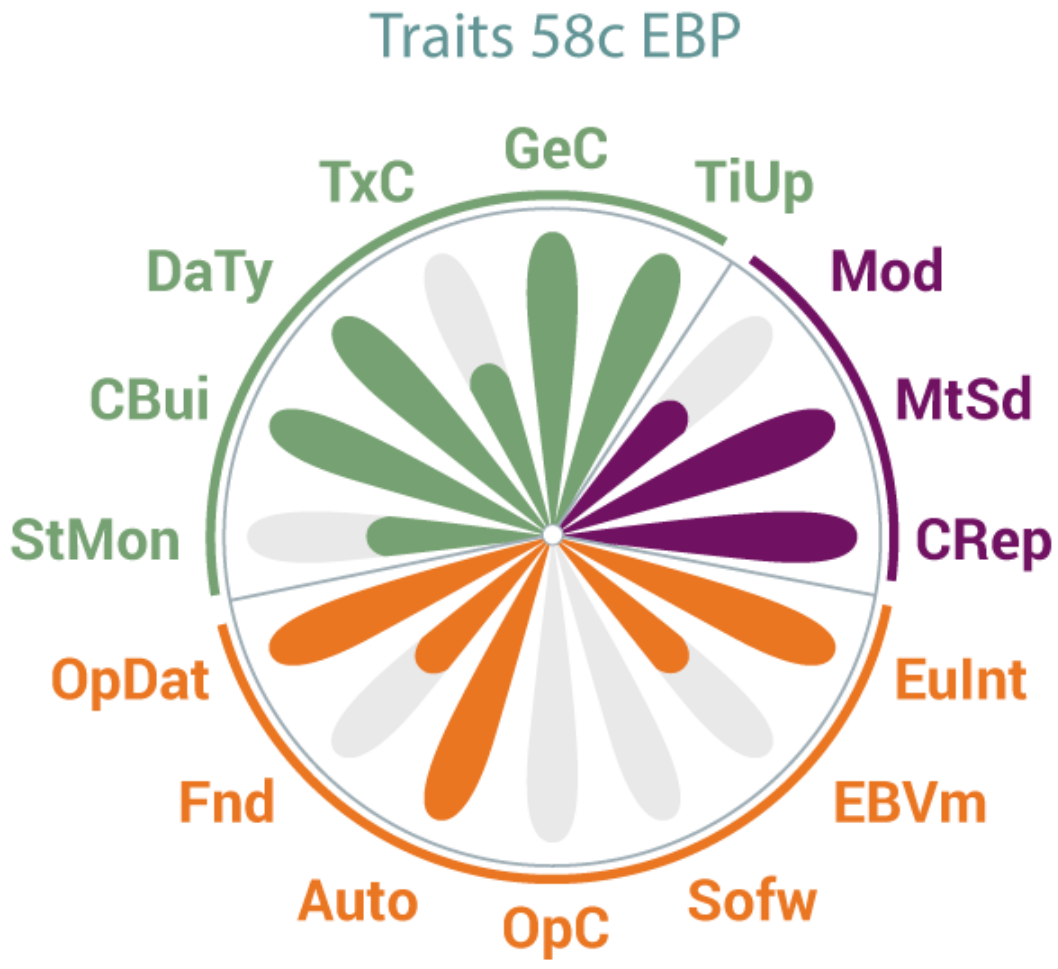

EURING coordinates bird ringing in Europe and centralizes the data collected by the different bird ringing schemes operating on the continent to promote and encourage its use for bird management and conservation. Ringing data are collected year-round following standardized protocols (e.g., EUROCES Constant Effort Sites) or no protocol at all. The data consist of bird ringings (when a ring was first added to a bird), recaptures (retraps of ringed birds by ringers) and recoveries/resightings (ringed bird reported by the public (e.g., dead birds, rings/marks read at a distance) which form the bulk of the data currently stored in the EURING databank (EDB). The recently launched Eurasian African Migration Atlas (Spina et al. 2022) highlights the value of the EURING data to understand bird movements in time and space while the new Migration Mapping Tool is a good example of the applied value of combining the connectivity information of EURING with the observational data from the EuroBirdPortal in single tool (Gargallo et al. 2022).

The data collected by EURING and the bird ringing schemes in general have the potential to obtain the metrics required for this EBV and, more relevantly, are probably the best suited to estimate length of stay (e.g. though mark-recapture analysis).

Possible bottlenecks in the generation of this EBV from existing data collected by EURING relate to:

1) Data collection & sampling. EURING collects data of most bird species occurring in Europe and, though the amount of data (particularly recaptures/recoveries) varies greatly among species, figures are quite high for several of them. Data is collected across all European countries, but the degree of coverage is poorer in the South and, particularly, in the East and South-East. Only part of the data is collected following standardized monitoring protocols (**StMon** partial

bottleneck). Training and capacity building for the network is provided during regular annual meetings or direct support to specific partners. Data in the EURINGdataback (EDB) are mostly updated once every year (**TiUp** partial bottleneck), although each record retains its temporal resolution (the date of data collection).

2) Models, data interoperability and IT infrastructure. Ringing data has been widely used to study phenology and length of stay and to obtain some of the metrics homologous to those required by this EBV for some specific uses, for example. To estimate the start of spring migration to delimit hunting seasons (e.g. the Migration seasons of hunted species research module of the Eurasian African Migration Atlas)(see also Ambrosini et al. 2014); however, so far, this has not been generalized nor automated within the framework of the initiative (**Mod** partial bottleneck). The EURING data flow takes place using the EURING Exchange Code standard, a pioneer of this kind among biodiversity data hubs in Europe, to ensure data harmonization and optimize its value. Data is centralized in the EURING databank.

3) Data Integration. EURING has already generated products that partially match the definition of this EBV (the Migration seasons of hunted species research module of the Eurasian African Migration Atlas, with predictions of prenuptial migration of different species at 10-day intervals - **EBVm** partial bottleneck). The data in the EDB have the required spatial and temporal resolution needed for the generation of this EBV. However, as the data is mostly updated once every year and therefore, this EBV could only be generated, at most, on a yearly basis. Though this integration initiative can produce metrics of the kind required for this EBV, there is no open code nor user-friendly software yet to report in this regard, as this modelling is not routinely integrated in this initiative (**Sofw** and **OpC** bottlenecks). Data streams are not automated yet: ringing centres across Europe send their ringing observations (data exports) once a year to the central repository on a manual basis (**Auto** bottleneck). Funding stability in the mid- long- term is not warranted (**Fnd** bottleneck). EDB data is available upon request and subject to agreement by National bird ringing schemes who hold the ownership of data (**OpDat** partial bottleneck), but just one centralized data request should be done as data is already centralized in the EDB (authorizations by national owners are coordinated by EBP).

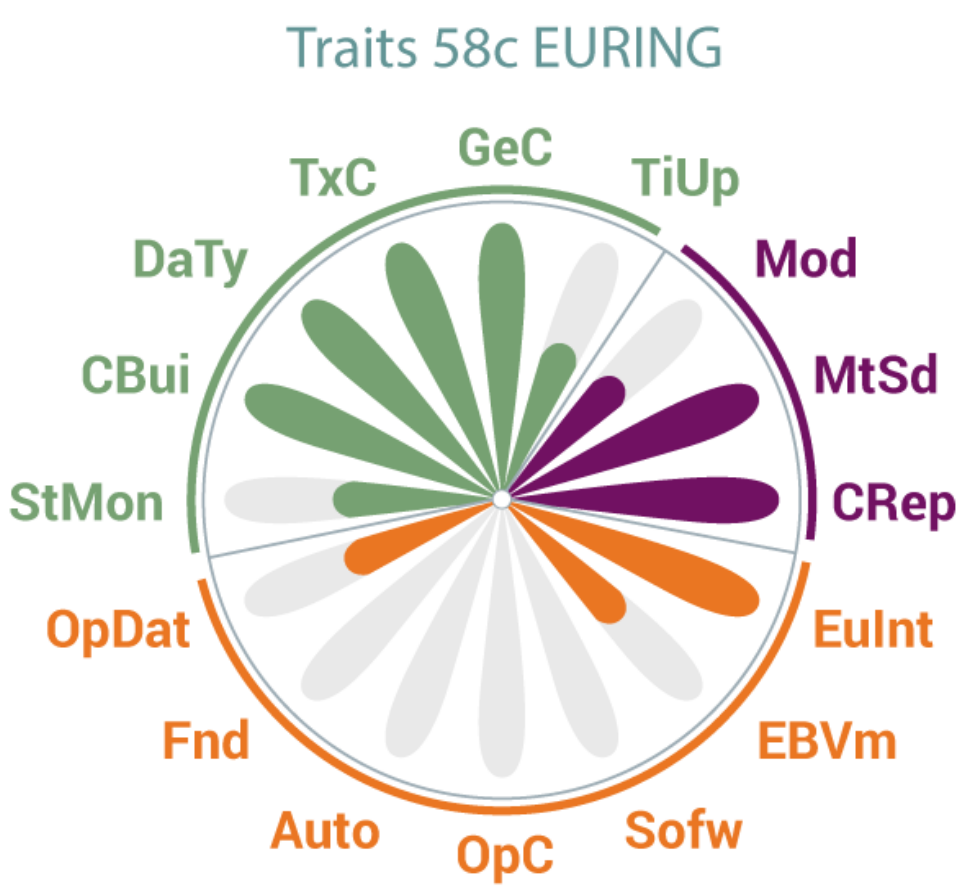

References:

Ambrosini, R., Borgoni, R., Rubolini, ... & Saino, N. (2014). Modelling the progression of bird migration with conditional autoregressive models applied to ringing data. *PloS one*, 9(7), e102440.

Gargallo, G., Davies, J.G., Faverjon, C., Kampichler C., Baillie, S.R., Cameron, A., Robinson, R.A. and Sierdsema, H. (2022) Development of a prototype early warning system for avian influenza in the EU based on risk-mapping. EFSA supporting publication 2022: EN-7762, 46pp.

Newson, S. E., Moran, N. J., Musgrove, A. J., Pearce-Higgins, J. W., Gillings, S., Atkinson, P. W., ... & Baillie, S. R. (2016). Long-term change in spring and autumn migration phenology of common migrant

breeding birds in Britain: results from large-scale citizen science bird recording schemes. *Ibis (Lond. 1859)*, *158*, 481-495.

Spina, F., Baillie, S.R., Bairlein, F, Fiedler, W. and Thorup, K. (Eds) 2022. *The Eurasian African Bird Migration Atlas*. https://migrationatlas.org. EURING/CMS.

Table S12. Freshwater EBV factsheets: Ecological Quality Ratio (EQR) of phytoplankton in lakes

**EBV: Ecological Quality Ratio (EQR) of phytoplankton in lakes**	
ID	9
Realm	Freshwater
EBV class	Community composition
EBV name	Ecological Quality Ratio (EQR) of phytoplankton in lakes
Step in identification process	User & Policy Needs Assessment
Definition	Community composition and total biomass of phytoplankton in lakes (Ecological Quality Ratio) based on total abundance (biovolume), taxonomic composition index across all species based on biovolume per indicator species, and bloom intensity, e.g., maximum biomass of cyanobacteria or percentage of cyanobacteria of the total biomass for all taxa.
Metric	The Ecological Quality Ratio (EQR) of phytoplankton in European lakes, expressed as a numerical value between zero (bad) and one (high), quantifying the ecological status of phytoplankton community composition and its deviation from a reference condition. The metric describes the deviation from natural phytoplankton communities.
Spatial resolution unit	Lakes as delineated in ECRINS (European catchments and rivers network system)
Temporal resolution unit	1 year, weekly-monthly during growing season
Taxonomic/ ecosystem focus group	Phytoplankton indicator taxa and reference taxa as described in the Water Framework Directive Intercalibration Technical Reports (Part 2, Lakes)

Main European initiatives and description of current bottlenecks

The **WISE-2** (Water Information System for Europe - Biology data), is an ongoing European monitoring data collection scheme established by the European Environment Agency (EEA) to obtain a harmonized flow of biology monitoring data from all EEA member states reported as Ecological Quality Ratios (EQRs) from all surface water categories: rivers, lakes, transitional and coastal waters. This data flow is tightly linked to the reporting duties of Member States within the regulations of the Water Framework Directive.

Possible bottlenecks in the generation of this EBV from existing data collected by WISE-2 data relate to:

1) Data collection and sampling. Currently, only 13 European countries report phytoplankton in lakes to the WISE-2 data flows so there is a geographic coverage gap; however, phytoplankton species are quite generalist and widespread so probably most if not all taxa within this group are covered by the monitoring of those 13 countries (**Gec** partial bottleneck). However, 26 countries monitor phytoplankton in lakes on an annual basis for the Water Framework Directive flows, so probably the geographic gaps are smaller than expected by WISE-2 data flows. The WISE-2 data flow, as an integration initiative, organizes yearly webinars for all member states with information and Q&A sessions on data reporting, but does not have capacity building on monitoring. If this exists, it is the capacity building of the member states (**CBui** bottleneck); this EBV requires highly specialized taxonomic expertise, and capacity building is indeed done by the countries but not all countries have the same capacity. Data collected for this taxonomic group is composition, abundance and biomass. Monitoring is carried out every year in some water bodies, but not in all, but EQRs are reported to the WISE system on an annual basis in all countries for water bodies monitored two or more years earlier than the year of reporting (there is not weekly -monthly reporting during the growing season; **TiUp** partial bottleneck).

2) Models, interoperability, and IT infrastructure. The use of models does not apply in EQR EBVs (**Mod** bottleneck): EQRs are estimated as a ratio between the value of the observed biological parameter for a given type of surface water body and the expected value under reference conditions. The ratio shall be expressed as a numerical value between 0 and 1, with high ecological status represented by values close to one (EQR > 0.8) and bad ecological status by values close to zero (EQR< 0.21). This ecological status value is the one that gets reported to the EEA. The EEA requires the countries to report their data to the CDR - Central Data Repository. The EQRs reported by member states are aggregated to various levels by EEA (ETC) to obtain overall EU values or by regions or groups of water bodies within the same status class or classes, e.g., WBs with EQR values corresponding to high and good status (EQR > 0.6), moderate status (EQR between 0.4 and 0.59) or poor+bad status (EQR > 0.21). Reporting by Member States follows metadata standards (the structure and contents of the data are described in a Data Dictionary).

3) Data integration. The phytoplankton EQRs reported under WISE-2 data flows partially match the definition of the EBV (although not in terms of temporal resolution as weekly-monthly data are not available; **EBVm** partial bottleneck). As there are not currently models in place to predict phytoplankton EQRs across European lakes, there is no open code or user-friendly software already being used to generate this EBV from WISE-2 data (**OpC** and **SoFw** bottlenecks). Data flows are not fully automated: the EEA provides empty templates for download from the data dictionary as Excel file and XML Schema. There is a reporting tool (ReportNet) where member states can upload these data. An automatic quality control (QR) process is implemented on the Central Data Repository to check the structure and content of the data file(s) uploaded by each member state. This first step of QC is followed by three more QC steps that involve: (2) cross-checking against previously reported data, (3) statistical tools and other algorithms for identifying suspicious values, and (4) expert-based checking and communication with data reporters. Automation of data flows between field data collection and national coordinators yet to be explored (some tests in Norway are underway) (**Auto** partial bottleneck). Funding has to be secured by member states to comply with reporting obligations. Phytoplankton EQR ratios at the waterbody level are openly available.

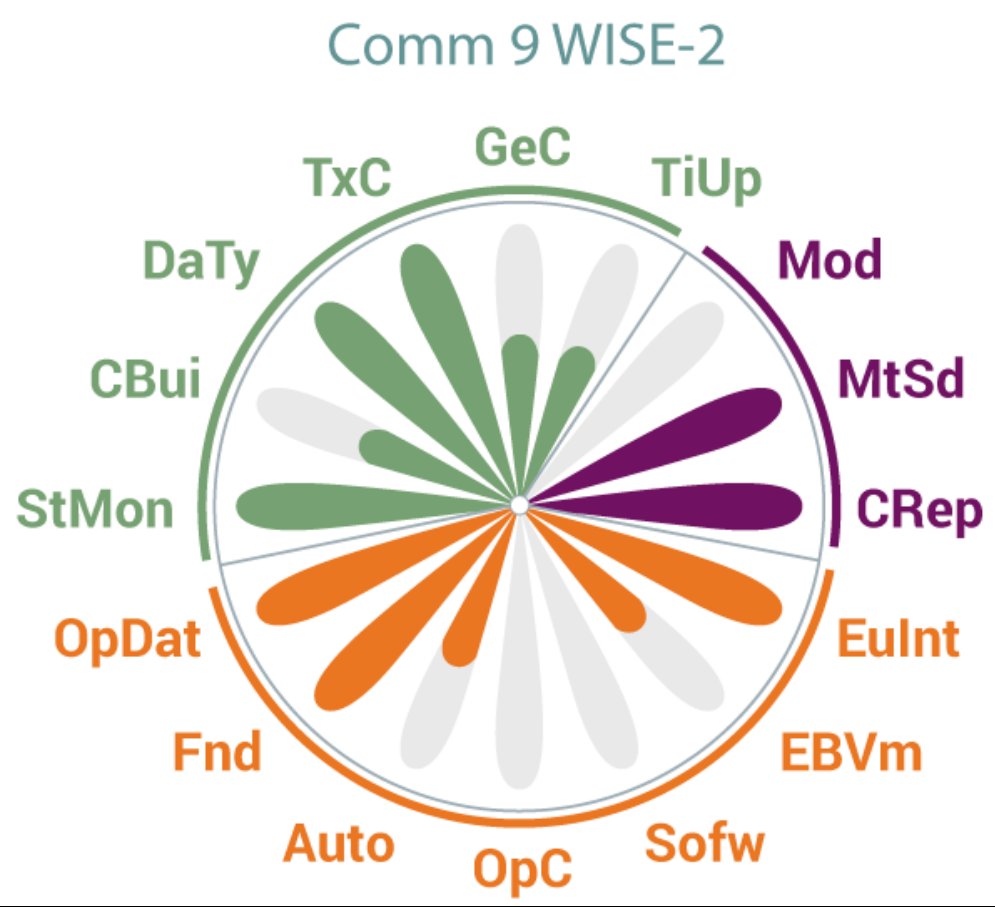

Table S13.Freshwater EBV factsheets: Ecological Quality Ratio (EQR) of freshwater macrophytes

**Ecological Quality Ratio (EQR) of freshwater macrophytes**	
ID	10
Realm	Freshwater
EBV class	Community composition
EBV name	Ecological Quality Ratio (EQR) of freshwater macrophytes
Step in identification process	User & Policy Needs Assessment
Definition	Community composition of macrophytes (Ecological Quality Ratio) based on presence-absence data.
Metric	The Ecological Quality Ratio (EQR) of macrophytes in European lakes, expressed as a numerical value between zero (bad) and one (very good), quantifying the ecological status of macrophyte community composition and its deviation from a reference condition.
Spatial resolution unit	Lakes as delineated in ECRINS (European catchments and rivers network system)
Temporal resolution unit	6 years
Taxonomic/ ecosystem focus group	All macrophytes species with indicator values as defined in the Water Framework Directive Intercalibration Technical Reports (Part 2, Lakes)

Main European initiatives and description of current bottlenecks

The **WISE-2** (Water Information System for Europe - Biology data), is an ongoing European monitoring data collection scheme established by the European Environment Agency (EEA) to obtain a harmonized flow of biology monitoring data from all EEA member states reported as Ecological Quality Ratios (EQRs) from all surface water categories: rivers, lakes, transitional and coastal waters. This data flow is tightly linked to the reporting duties of Member States within the regulations of the Water Framework Directive.

Possible bottlenecks in the generation of this EBV from existing data collected by WISE-2 data relate to:

1) Data collection and sampling. Currently, only 15 European countries report Macrophytes in lakes to the WISE-2 data flows so there is a geographic coverage gap that probably translates into a taxonomic coverage gap (some countries only report EQRs for this taxonomic group intermittently) (**Gec**, **TxC** partial bottlenecks). However, 19 countries monitor macrophytes in lakes on an annual basis for the Water Framework Directive flows, so probably the geographic gaps are smaller than expected by WISE-2 data flows. The WISE-2 data flow, as an integration initiative, organizes yearly webinars for all member states with information and Q&A sessions on data reporting, but does not have capacity building on monitoring. If this exists, it is the capacity building of the member states (**CBui** bottleneck). Data collected for this taxonomic group is mostly presence-absence, with some measures of relative abundance. Monitoring is carried out every year in some water bodies, but not in all, but EQRs are reported to the WISE system on an annual basis in all countries for water bodies monitored two or more years earlier than the year of reporting.

2) Models, interoperability, and IT infrastructure. The use of models does not apply in EQR EBVs (**Mod** bottleneck): EQRs are estimated as a ratio between the value of the observed biological parameter for a given type of surface water body and the expected value under reference conditions. The ratio shall be expressed as a numerical value between 0 and 1, with high ecological status represented by values close to one (EQR > 0.8) and bad ecological status by values close to zero (EQR< 0.21). This ecological status value is the one that gets reported to the EEA. The EEA requires the countries to report their data to the CDR - Central Data Repository. The EQRs reported by member states are aggregated to various levels by EEA

(ETC) to obtain overall EU values or by regions or groups of water bodies within the same status class or classes, e.g., WBs with EQR values corresponding to high and good status (EQR > 0.6), moderate status (EQR between 0.4 and 0.59) or poor+bad status (EQR > 0.21). Reporting by Member States follows metadata standards (the structure and contents of the data are described in a Data Dictionary).

3) Data integration. The Macrophyte EQRs reported under WISE-2 data flows perfectly match the definition of the EBV. As there are not currently models in place to predict Macrophyte EQRs across European lakes, there is no open code or user-friendly software already being used to generate this EBV from WISE-2 data (**OpC** and **SoFw** bottlenecks). Data flows are not fully automated: the EEA provides empty templates for download from the data dictionary as Excel file and XML Schema. There is a reporting tool (ReportNet) where Member States can upload these data. An automatic quality control (QC) process is implemented on the Central Data Repository, to check the structure and content of the data file(s) uploaded by each member state. This first step of QC is followed by three more QC steps that involve: (2) cross-checking against previously reported data, (3) statistical tools and other algorithms for identifying suspicious values, and (4) expert-based checking and communication with data reporters. Automation of data flows between field data collection and national coordinators yet to be explored (some tests in Norway are underway) (**Auto** partial bottleneck). Funding must be secured by member states to comply with reporting obligations. While Macrophytes EQR ratios at the waterbody level are openly available, raw data at the site level (composition and abundance values) are not.

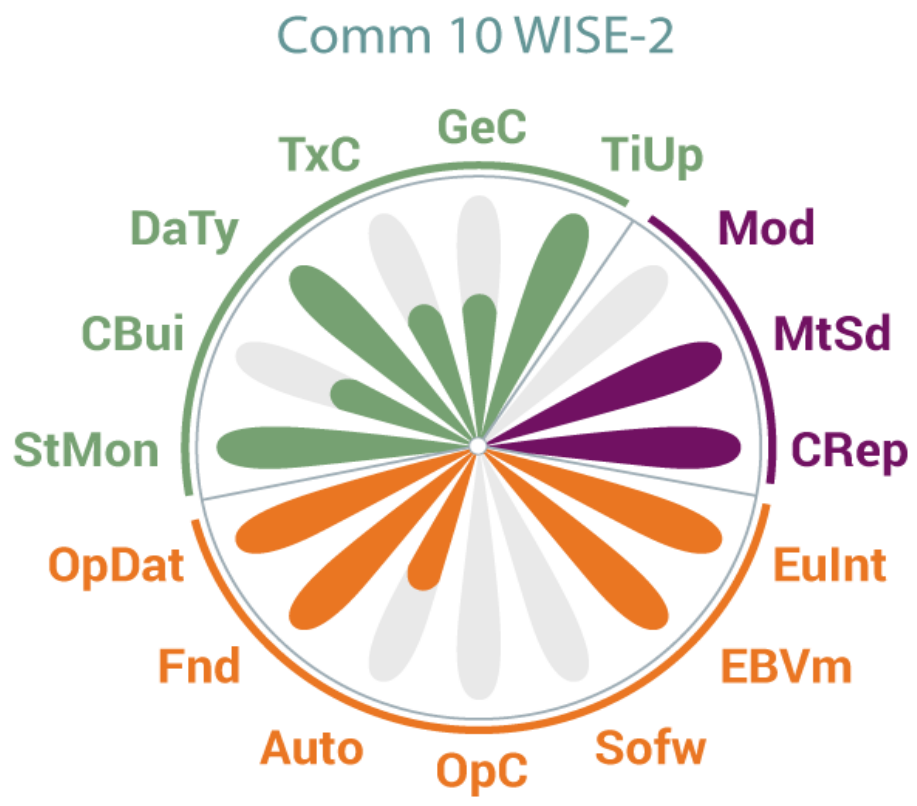

Table S14. Freshwater EBV factsheets: Ecological Quality Ratio (EQR) of freshwater phytobenthos

**EBV: Ecological Quality Ratio (EQR) of freshwater phytobenthos**	
ID	11
Realm	Freshwater
EBV class	Community composition
EBV name	Ecological Quality Ratio (EQR) of freshwater phytobenthos
Step in identification process	User & Policy Needs Assessment
Definition	The ecological status of phytobenthos in European rivers, measured as Ecological Quality Ratio (EQR).
Metric	The Ecological Quality Ratio (EQR) of phytobenthos in European rivers, expressed as a numerical value between zero (bad) and one (very good), quantifying the ecological status of phytobenthos community composition and its deviation from a reference condition.
Spatial resolution unit	River catchments as delineated in ECRINS (European catchments and Rivers network system)
Temporal resolution unit	1-3 years
Taxonomic/ ecosystem focus group	Phytobenthic species with indicator values as defined in the Water Framework Directive Intercalibration Technical Reports (Part 1, Rivers)

Main European initiatives and description of current bottlenecks

The **WISE-2** (Water Information System for Europe - Biology data), is an ongoing European monitoring data collection scheme established by the European Environment Agency (EEA) to obtain a harmonized flow of biology monitoring data from all EEA member states reported as Ecological Quality Ratios (EQRs) from all surface water categories: rivers, lakes, transitional and coastal waters. This data flow is tightly linked to the reporting duties of Member States within the regulations of the Water Framework Directive.

Possible bottlenecks in the generation of this EBV from existing data collected by WISE-2 data relate to:

1) Data collection and sampling. Currently, only 22 European countries report phytobenthos in rivers to the WISE-2 data flows so there is a geographic coverage gap that probably translates into a taxonomic coverage gap (some countries only report EQRs for this taxonomic group intermittently) (**GeC**, **TxC partial** bottlenecks). The WISE-2 data flow, as an integration initiative, organises yearly webinars for all member states with information and Q&A sessions on data reporting, but does not have capacity building on monitoring. If this exists, it is the capacity building of the member states (**CBui** bottleneck); Data collected for this taxonomic group is mostly presence- absence with some measures of relative abundance. Monitoring is carried out every year in some water bodies, but not in all, but EQRs are reported to the WISE system on an annual basis in all countries for water bodies monitored two or more years earlier than the year of reporting.

2) Models, interoperability, and IT infrastructure. The use of models does not apply in EQR EBVs (**Mod** bottleneck): EQRs are estimated as a ratio between the value of the observed biological parameter for a given type of surface water body and the expected value under reference conditions. The ratio shall be expressed as a numerical value between 0 and 1, with high ecological status represented by values close to one (EQR > 0.8) and bad ecological status by values close to zero (EQR< 0.21). This ecological status value is the one that gets reported to the EEA. The EEA requires the countries to report their data to the CDR - Central Data Repository. The EQRs reported by member states are aggregated to various levels by EEA (ETC) to obtain overall EU values or by regions or groups of water bodies within the same status class or classes, e.g., WBs with EQR values corresponding to high and good status (EQR

> 0.6), moderate status (EQR between 0.4 and 0.59) or poor+bad status (EQR > 0.21). Reporting by Member States follows metadata standards (the structure and contents of the data are described in a Data Dictionary).

3) Data integration. The phytobenthos EQRs reported under WISE-2 data flows perfectly match the definition of the EBV. As there are not currently models in place to predict phytobentos EQRs across European rivers,there is no open code or user-friendly software already being used to generate this EBV from WISE-2 data (**OpC** and **SoFw** bottlenecks). Data flows are not fully automated: the EEA provides empty templates for download from the data dictionary as Excel file and XML Schema. There is a reporting tool (ReportNet) where to upload these data. An automatic quality control (QC) process is implemented on the Central Data Repository, to check the structure and content of the data file(s) uploaded by each member state. This first step of QC is followed by three more QC steps that involve: (2) cross-checking against previously reported data, (3) statistical tools and other algorithms for identifying suspicious values, and (4) expert-based checking and communication with data reporters. Automation of data flows between field data collection and national coordinators yet to be explored (some tests in Norway are underway) (**Auto** partial bottleneck). Funding has to be secured by member states to comply with reporting obligations. Phytobentos EQR ratios at the waterbody level are openly available; however, raw data (composition and abundance values are not.

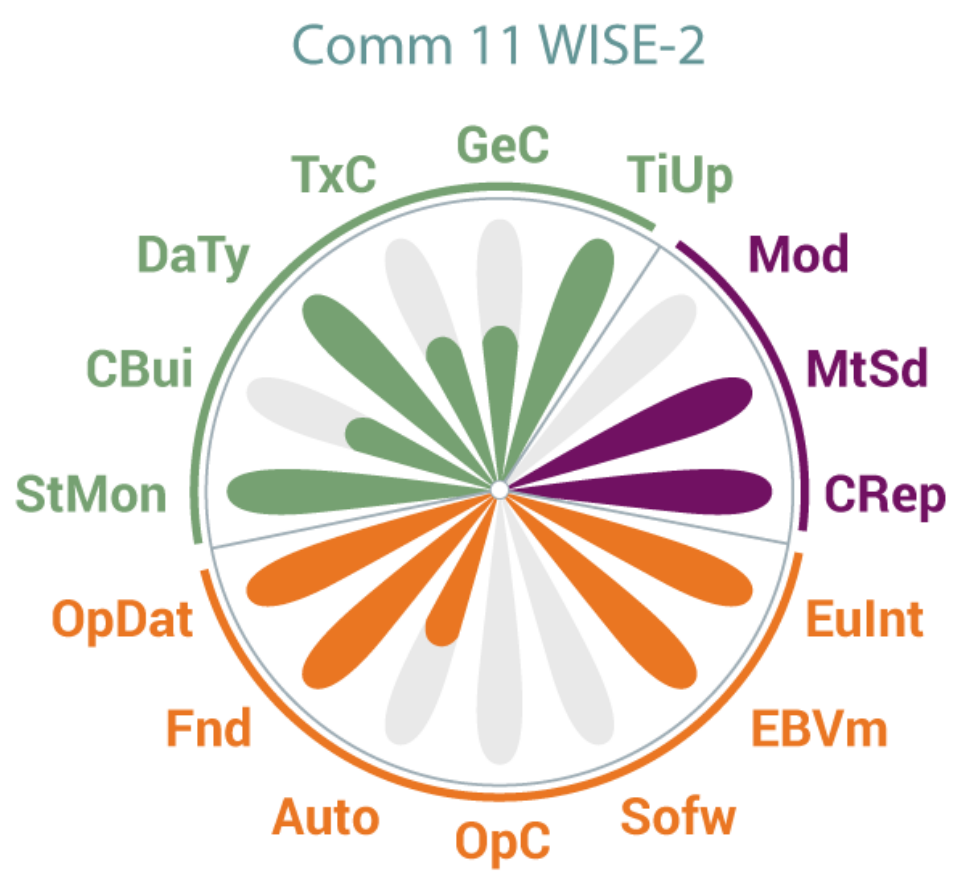

Table S15. Freshwater EBV factsheets: Ecological Quality Ratio (EQR) of benthic freshwater invertebrates

**EBV: Ecological Quality Ratio (EQR) of benthic freshwater invertebrates**	
ID	12
Realm	Freshwater
EBV class	Community composition
EBV name	Ecological Quality Ratio (EQR) of benthic freshwater invertebrates
Step in identification process	User & Policy Needs Assessment
Definition	The ecological status of benthic invertebrates in European rivers, measured as Ecological Quality Ratio (EQR).
Metric	The Ecological Quality Ratio (EQR) of benthic invertebrates in European rivers, expressed as a numerical value between zero (bad) and one (very good), quantifying the ecological status of benthic invertebrates' community composition and its deviation from a reference condition.
Spatial resolution unit	River catchments as delineated in ECRINS (European catchments and rivers network system)
Temporal resolution unit	2-3 years
Taxonomic/ ecosystem focus group	Benthic invertebrate species with indicator values as defined in the Water Framework Directive Intercalibration Technical Reports (Part 1, Rivers)

Main European initiatives and description of current bottlenecks

The **WISE-2** (Water Information System for Europe - Biology data), is an ongoing European monitoring data collection scheme established by the European Environment Agency (EEA) to obtain a harmonized flow of biology monitoring data from all EEA member states reported as Ecological Quality Ratios (EQRs) from all surface water categories: rivers, lakes, transitional and coastal waters. This data flow is tightly linked to the reporting duties of Member States within the regulations of the Water Framework Directive.

Possible bottlenecks in the generation of this EBV from existing data collected by WISE-2 data relate to:

1) Data collection and sampling. Currently, only 25 European countries report benthic invertebrates in rivers to the WISE-2 data flows, so there is a geographic coverage gap that probably translates into a taxonomic coverage gap (some countries only report EQRs for this taxonomic group intermittently) (**Gec**, **TxC** partial bottlenecks). However, 28 countries monitor benthic invertebrates in rivers on an annual basis for the Water Framework Directive flows, so probably the geographic gaps are smaller than expected by WISE-2 data flows. The WISE-2 data flow, as an integration initiative, organizes yearly webinars for all member states with information and Q&A sessions on data reporting, but does not have capacity building on monitoring. If this exists, it is the capacity building of the member states (**CBui** partial bottleneck); Data collected for this taxonomic group is composition and abundance. Monitoring is carried out every year in some water bodies, but not in all, but EQRs are reported to the WISE system on an annual basis in all countries for water bodies monitored two or more years earlier than the year of reporting.

2) Models, interoperability, and IT infrastructure. Currently, the use of models does not apply in EQR EBVs (**Mod** bottleneck): EQRs are estimated as a ratio between the value of the observed biological parameter for a given type of surface water body and the expected value under reference conditions. The ratio shall be expressed as a numerical value between 0 and 1, with high ecological status represented by values close to one (EQR > 0.8) and bad ecological status by values close to zero (EQR< 0.21). This ecological status value is the one that gets reported to the EEA. The EEA requires the countries to report their data to the CDR - Central Data Repository. The EQRs reported by member states are aggregated to various levels by

EEA (ETC) to obtain overall EU values or by regions or groups of water bodies within the same status class or classes, e.g., WBs with EQR values corresponding to high and good status (EQR > 0.6), moderate status (EQR between 0.4 and 0.59) or poor+bad status (EQR > 0.21). However, for these taxa, there are some modelling initiatives underway to extrapolate EQR values from monitoring sites to non-monitored water bodies, using as reference the EQR values reported to WISE-2 data flows and considering predictors such as land use and abiotic variables (e.g., nutrient concentrations). Reporting by Member States follows metadata standards (the structure and contents of the data are described in a Data Dictionary).

3) Data integration. The benthic invertebrate EQRs reported under WISE-2 data flows perfectly match the definition of the EBV. As there are not currently models in place to predict freshwater invertebrates EQR values across European rivers, there is no open code or user-friendly software already being used to generate this EBV from WISE-2 data (**OpC** and **SoFw** bottlenecks). Data flows are not fully automated: the EEA provides empty templates for download from the data dictionary as Excel file and XML Schema. There is a reporting tool (ReportNet) where Member States can upload these data. An automatic quality control (QC) process is implemented on the Central Data
Repository, to check the structure and content of the data file(s) uploaded by each member state. This first step of QC is followed by three more QC steps that involve: (2) cross-checking against previously reported data, (3) statistical tools and other algorithms for identifying suspicious values, and (4) expert-based checking and communication with data reporters. Automation of data flows between field data collection and national coordinators yet to be explored (some tests in Norway are underway) (**Auto** partial bottleneck). Funding must be secured by member states to comply with reporting obligations. Benthic invertebrate EQR ratios at the waterbody level are openly available, but raw data are not (composition and abundance values).

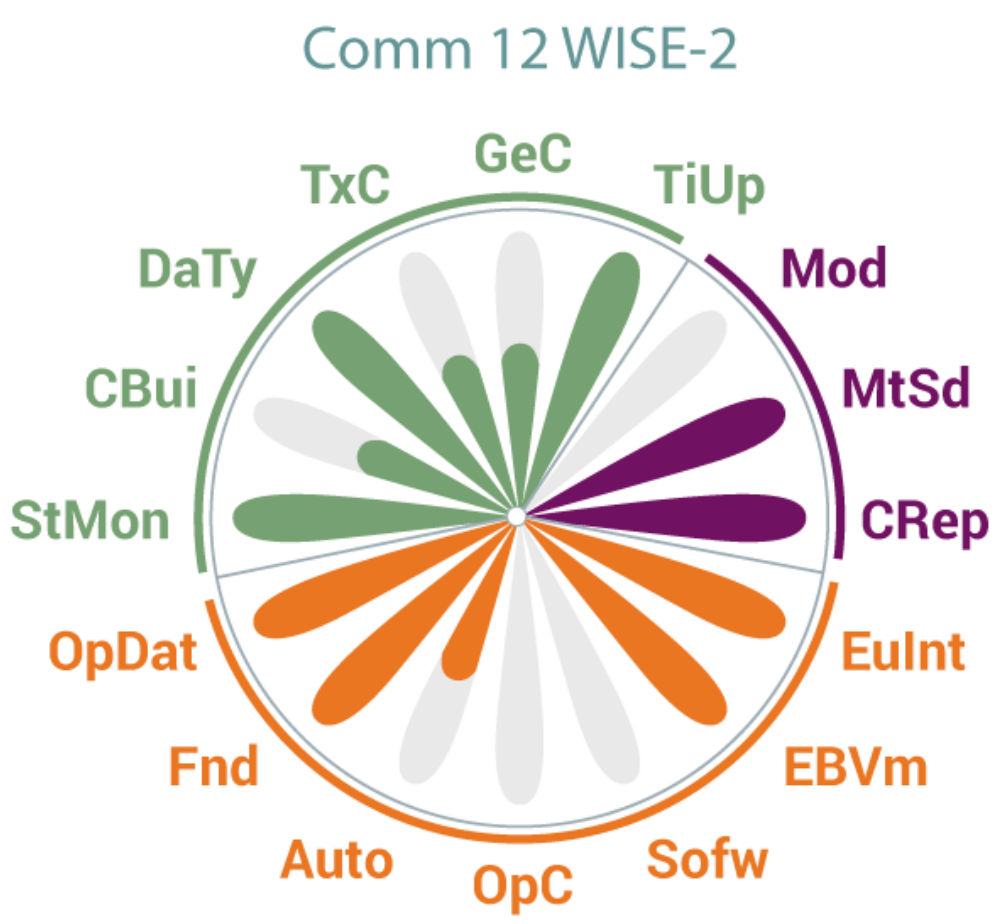

Table S16.Freshwater EBV factsheets: Ecological Quality Ratio (EQR) of freshwater fish

**EBV: Ecological Quality Ratio (EQR) of freshwater fish**	
ID	13
Realm	Freshwater
EBV class	Community composition
EBV name	Ecological Quality Ratio (EQR) of freshwater fish
Step in identification process	User & Policy Needs Assessment
Definition	The ecological status of fish in European freshwater systems (lakes and rivers), measured as Ecological Quality Ratio (EQR).
Metric	The Ecological Quality Ratio (EQR) of fish in European lakes and rivers, expressed as a numerical value between zero (bad) and one (very good), quantifying the ecological status of fish community composition and its deviation from a reference condition.
Spatial resolution unit	Lakes and rivers catchments as delineated in ECRINS (European catchments and rivers network system)
Temporal resolution unit	3 – 6 years
Taxonomic/ ecosystem focus group	Freshwater fish species with indicator values defined in the Water Framework Directive Intercalibration Technical Reports (Part 1, Rivers; Part2, lakes)

Main European initiatives and description of current bottlenecks

The **WISE-2** (Water Information System for Europe), is an ongoing European monitoring scheme established by the European Environment Agency (EEA) to obtain a harmonized flow of biology data from all EEA member states reported as Ecological Quality Ratios (EQRs) from all surface water categories: rivers, lakes, transitional and coastal waters. This data flow is tightly linked to the reporting duties of Member States within the regulations of the Water Framework Directive.

Possible bottlenecks in the generation of this EBV from existing data collected by WISE-2 data relate to:

1) Data collection and sampling. Currently, only 10 European countries report FISH_EQRs to the WISE-2 so there is a gap in the reporting of fish data to the European Commission, that probably translates into a gap in taxonomic coverage given many fish species are endemic of specific river catchments (**GeC, TxC** partial bottlenecks). However, 27 countries collect data on an annual basis on fish species for the Water Framework Directive flows, so probably the geographic and taxonomic monitoring gaps are smaller than expected by WISE-2 data flows and Fish EQRs could be reported by a larger number of countries. The WISE-2 data flow, as an integration initiative, organises yearly webinars for all member states with information and Q&A sessions on data reporting, but does not have capacity building on monitoring. If this exists, it is the capacity building of the member states (**CBui** partial bottleneck). Data collected for this taxonomic group is composition, abundance and age structure. Monitoring is carried out every year in some water bodies, but not in all, but EQRs are reported to the WISE system on an annual basis in all countries for water bodies monitored two or more years earlier than the year of reporting.

2) Models, interoperability, and IT infrastructure. The use of models does not apply in EQR EBVs (**Mod** bottleneck): EQRs are estimated as a ratio between the value of the observed biological parameter for a given type of surface water body and the expected value under reference conditions. The ratio shall be expressed as a numerical value between 0 and 1, with high ecological status represented by values close to one (EQR > 0.8) and bad ecological status by values close to zero (EQR < 0.21). This ecological status value is the one that gets reported to the EEA. The EEA requires the countries to report their data to the CDR - Central Data Repository. The EQRs reported by member states are aggregated to various levels by EEA (ETC) to obtain overall EU values or by regions or groups of water bodies within the same

status class or classes, e.g., WBs with EQR values corresponding to high and good status (EQR > 0.6), moderate status (EQR between 0.4 and 0.59) or poor+bad status (EQR > 0.21). Reporting by Member States follows metadata standards (the structure and contents of the data are described in a Data Dictionary).

3) Data integration. The fish EQRs reported under WISE-2 data flows perfectly match the definition of the EBV. As there are not currently models in place to predict fish EQR values across European lakes and rivers, there is no open code or user-friendly software already being used to generate this EBV from WISE-2 data (**OpC** and **SoFw** bottlenecks). Data flows are not fully automated: the EEA provides empty templates for download from the data dictionary as Excel file and XML Schema. There is a reporting tool (ReportNet) where to upload these data. An automatic quality control (QC) process is implemented on the Central Data Repository, to check the structure and content of the data file(s) uploaded by each member state. This first step of QC is followed by three more QC steps that involve: (2) cross-checking against previously reported data, (3) statistical tools and other algorithms for identifying suspicious values, and (4) expert-based checking and communication with data reporters. Automation of data flows between field data collection and national coordinators yet to be explored (some tests in Norway are underway) (**Auto** partial bottleneck). Funding must be secured by member states to comply with reporting obligations. FISH_EQR ratios at the waterbody level are openly available, but raw data are not (composition, abundance and age structure measures).

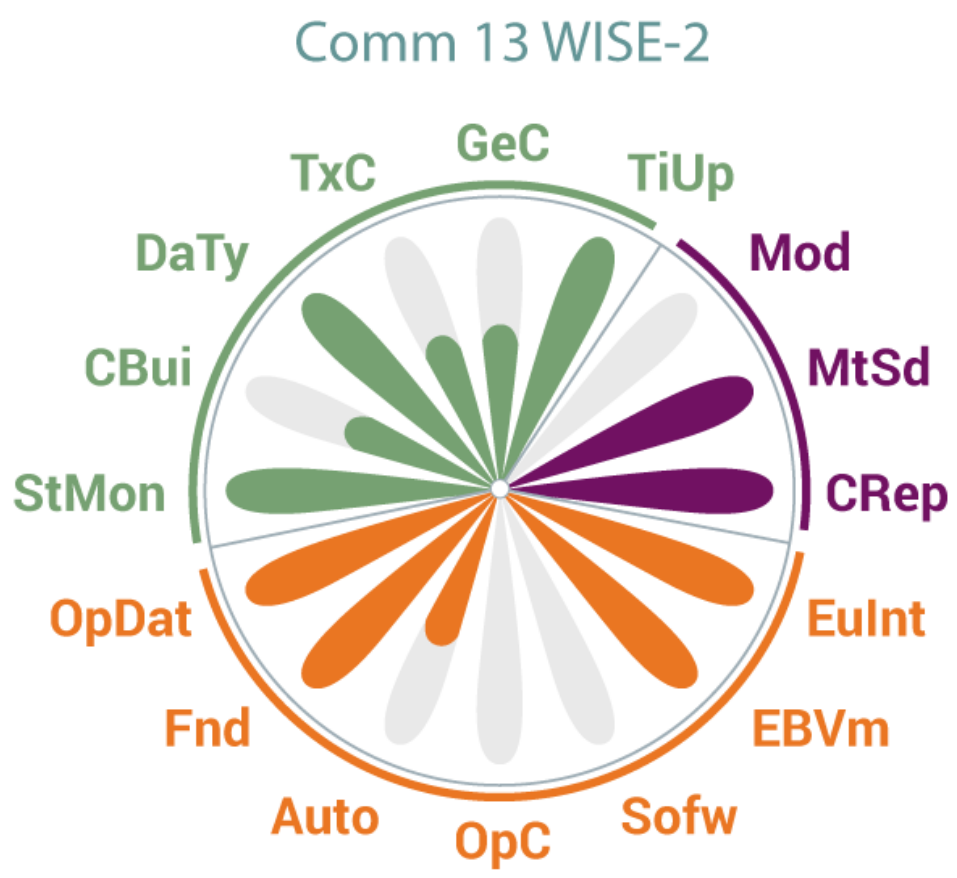

Table S17. Freshwater EBV factsheets: Harmful freshwater algal blooms

**EBV: Harmful freshwater algal blooms**	
ID	19
Realm	Freshwater
EBV class	Ecosystem function
EBV name	Harmful freshwater algal blooms
Step in identification process	User & Policy Needs Assessment
Definition	Distribution, intensity, frequency and position of harmful algal blooms in European lakes which occur when cyanobacteria accumulate in water, with the potential to harm the health of humans, plants, and animals.
Metric	- Observed location and intensity of algal blooms as derived from satellite imagery (e.g. Copernicus Sentinel-3) or regulatory monitoring<br>- Modeled cyanobacterial density based on hydrodynamic models and satellite imagery
Spatial resolution unit	Lakes as delineated in ECRINS (European catchments and Rrivers network system)
Temporal resolution unit	Real-time, weekly-monthly during the growing season
Taxonomic/ ecosystem focus group	ECRINS lakes

Main European initiatives and description of current bottlenecks

Regulatory monitoring of algal blooms (Cyanobacteria) is made as part of the Water Framework Directive Lake phytoplankton monitoring to assess phytoplankton status class. Cyanobacteria biovolume (in mg L-1 or mm3 L-1) or the % of total biovolume in lakes are reported to the Water Information System for Europe - Water Quality (WISE-6) - an ongoing European monitoring scheme established by the European Environment Agency (EEA) to obtain a harmonized flow of water quality data in groundwater, rivers, lakes, transitional, coastal and marine waters. Includes data on nutrients, organic matter, chlorophyll-a, hazardous substances, and general physico-chemical parameters in water, sediment, and biota. Observed presence and intensity of algal blooms derived from satellite imagery (e.g. Copernicus Sentinel-3) is also possible, but public services are still under-development (this is an area “under development” for GEO AQuaWatch and the Copernicus Global Land Service (CGLS) for lake water quality Lake Water Quality | Copernicus Global Land Service.

Possible bottlenecks in the generation of this EBV from regulatory monitoring collected by WISE-6 data relate to:

1) Data collection and sampling. Currently, while 26 countries monitor phytoplankton in lakes to report ecological status to the Water Framework Directly, only 5 European countries report cyanobacteria to the WISE-6 data flows, so there is a geographic coverage gap in WISE-6 data flows for these taxa (**GeC** partial bottlenecks). The WISE-6 data flow, as an integration initiative, organizes yearly webinars for all member states with information and Q&A sessions on data reporting, but does not have capacity building on monitoring. If this exists, it is the capacity building of the member states (**CBui** partial bottleneck). Data collected for this taxonomic group is Cyanobacteria biovolume (in mg L-1 or mm3 L-1) that could potentially be used for modelling purposes. Monitoring is carried out every year in some water bodies, but not in all. Cyanobacteria biovolume is reported to the WISE system on an annual basis. Regulatory monitoring for the Water Framework Directive is currently available monthly for the summer months (July to September) for one or two years every six years. However, the sample frequency is not enough to generate this EBV in real time (**TiUp** bottleneck). Near real-time or weekly monitoring may become available in future years through satellite EO (CGLS) and citizen science.

2) Models, interoperability, and IT infrastructure. Modelled cyanobacteria density in European lakes based on lake type, climate, and water quality (total phosphorus) data is possible using published statistical models (Richardson et al., 2018), but it is still not available through WISE-6 data flows (**Mod** bottleneck). Reporting by Member States follows metadata standards (the structure and contents of the data are described in a Data Dictionary) and is stored in the WISE data repository.

3) Data integration. The data collected and reported via WISE-6 does not perfectly match the EBV description, especially in terms of temporal resolution (**EBVm** partial bottleneck). Since models are not currently in place to predict the cyanobacterial density at WISE-6, there is no open code or user-friendly software to report here (**OpC** and **SoFw** bottlenecks). Data flows are not fully automated: the EEA provides empty templates for download from the data dictionary as Excel file and XML Schema. There is a reporting tool (ReportNet) where Member States can upload these data. An automatic quality control (QC) process is implemented on the Central Data Repository, to check the structure and content of the data file(s) uploaded by each member state. This first step of QC is followed by three more QC steps that involve: (2) cross-checking against previously reported data, (3) statistical tools and other algorithms for identifying suspicious values, and (4) expert-based checking and communication with data reporters. Automation of data flows between field data collection and national coordinators is yet to be explored (some tests in Norway are underway); moreover, citizen science data are available for this taxa in 5 countries in Europe (Belgium, Ireland, Netherlands, Norway, and UK) using the Bloomin' Algae app. Data provide high spatial resolution (10-20 m) and potentially high frequency (daily data), but data are qualitative (presence/absence of blooms judged by experts on photographic evidence) and do not provide quantitative information on biovolume or % abundance. The data are viewable, and raw data (location, date) can be made available in real-time through an API from iRecord (**Auto** partial bottleneck). Funding must be secured by member states to comply with reporting obligations. Cyanobacteria biovolume is available upon request for all the countries reporting for WISE-6 (**OpDat** partial bottleneck). Cyanobacteria is not reported as a separate metric to the WFD but is part of the metrics used to assess lake phytoplankton status class.

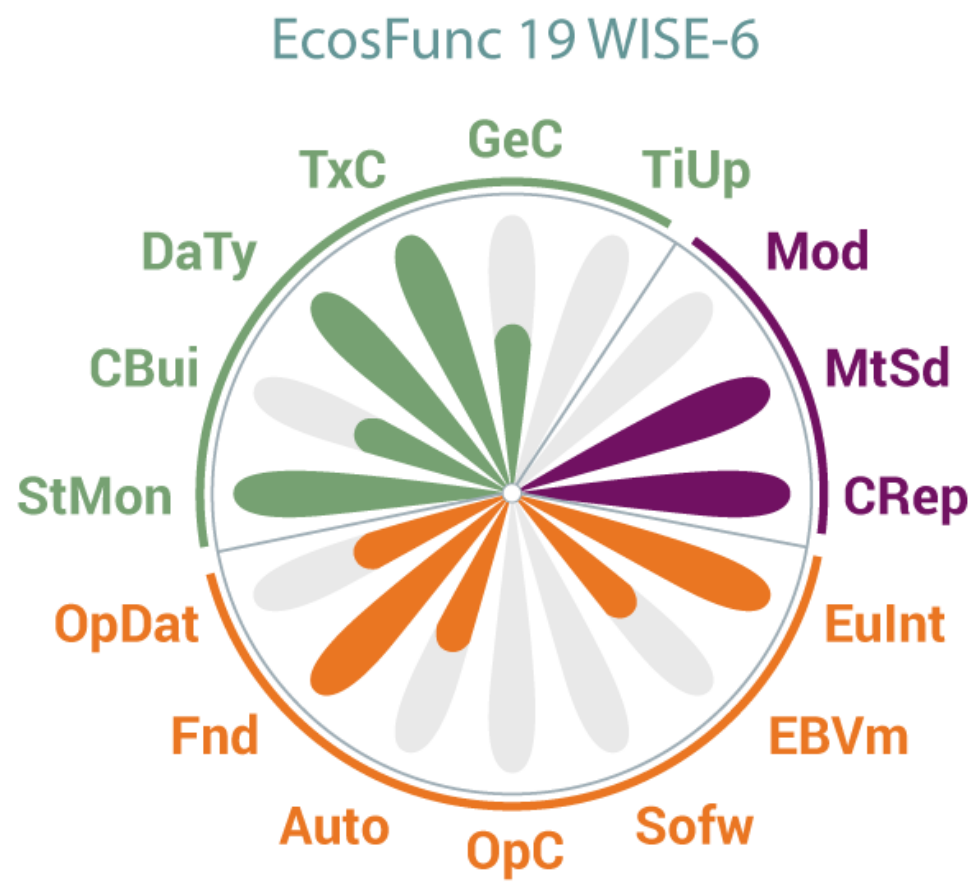

References:

Richardson, J., Miller, C., Maberly, S. C., Taylor, P., Globevnik, L., Hunter, P., ... and Carvalho, L. (2018). Effects of multiple stressors on cyanobacteria abundance vary with lake type. *Global Change Biology,* 24(11), 5044-5055.

Marine EBV factsheets

Table S18. Marine EBV factsheets: Species Distributions of Marine Fishes

**EBV: Species Distributions of Marine Fishes**	
ID	23
Realm	Marine
EBV class	Species Populations
EBV name	Species distributions of marine fishes
Step in identification process	User & Policy Needs Assessment
Definition	The presence/absence or probability of occurrence of European Marine fish species in EU's marine waters within contiguous spatial units (grid cells) over time.
Metric	- Binary presence/absence<br>- Probability of occurrence
Spatial resolution unit	50 × 50 km – 200 × 200 km
Temporal resolution unit	3 or 6 years
Taxonomic/ ecosystem focus group	Marine fish species indicated in the Review and Analyses of Member States 2018 reports Descriptor 1: Species biological diversity

Main European initiatives and description of current bottlenecks

There is not a single monitoring network integrating data of marine fish across the whole of Europe. However, there are different regional integration initiatives collecting data on the presence and abundance of marine fish that could potentially provide data to generate this EBV. Among them, we highlight here the fish data gathered by the International Council for the Exploitation of the Sea (ICES), corresponding to surveys undertaken through trawls (the main source of fish monitoring data in Europe - Jessop et al. 2022). While these are mostly directed to commercial species (see fact sheet of EBV 24 - *Species abundances of marine commercial fish species and long-distance migratory fishes, Table S19*), data from other species are also recorded, and individual species are recorded via a unique FishID code.

Possible bottlenecks in the generation of this EBV from ICES data relate to:

1) Data collection and sampling. The International Council for the Exploitation of the Sea (ICES) holds a database on pelagic and bottom fish trawl surveys collected in the North-east Atlantic and Baltic seas (DATRAS online database). Survey data comes from standardized survey protocols but these differ across regions (e.g. between the Baltic and the Western and Southern Areas) (**StMon** partial bottleneck). Data reported to DATRAS includes the coordinates of the shooting and hauling locations, a species ID and information on the age disaggregated abundance of fish species that could potentially be used to generate this EBV. The survey data currently covers the Baltic Sea, Skagerrak, Kattegat, North Sea, English Channel, Celtic Sea, Irish Sea, Bay of Biscay and the eastern Atlantic from the Shetlands to Gibraltar; therefore, the geographic coverage of data is not complete as it does not include data from the Mediterranean sea (**GeC** partial bottleneck); this translates in the lack of representation of Mediterranean species in the database (**TxC** partial bottleneck). There are more than 45 years of continuous time series data in DATRAS, and survey data are continuously updated by national institutions, so the data availability matches the temporal resolution sought by this EBV.

2) Models, interoperability, and IT infrastructure. ICES does not use models to generate spatially explicit distribution maps of fish species. Data are used to estimate different species abundance indices (**Mod** bottleneck). The DATRAS dataset has defined a vocabulary and a set of standards for data reporting to ease data handling, integration and availability but there

are not common metadata standards followed across all surveys (**MtSd** partial bottleneck). Data is centralized and stored in the ICES data portal.

3) Data integration. There is not a single integration initiative that collects and processes data of marine fishes' abundance and distributions across Europe (only regional and national ones, being the Mediterranean Sea clearly underrepresented) (**EuInt** partial bottleneck). The products generated from DATRAS (i.e., indices of fish stock abundance estimates of commercial species) do not match this EBV (**EBVm** bottleneck). Since there are not currently models in place to map the distribution of marine fish species, there is no open code or user-friendly software to report here (**OpC** and **SoFw** bottlenecks). Data streams are not fully automated: worksheets in .csv format are submitted to the DATRAS via the ICES platform but there is little information about the level of automatization of the data flows of the underlying datasets (**Auto** partial bottleneck). If funding for adequate survey effort delivering the data exists, it is that of the countries reporting data to DATRAS and ICES (**Fnd** bottleneck). DATRAS data products (such as Catch per area of fishing effort estimates - CPUE- or indices) and raw data, can be freely downloaded according to the ICES Data policy.

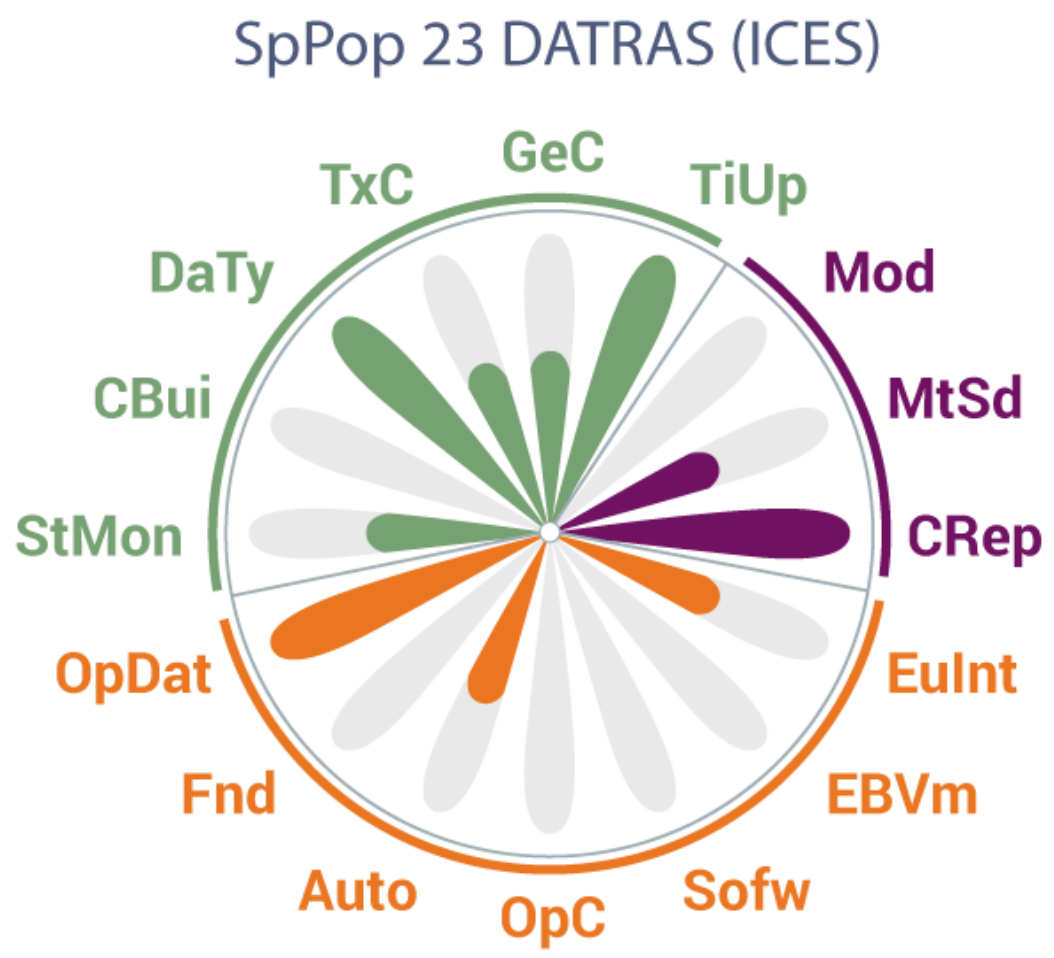

See Jessop et al. (2022) for a summary of other national and regional fish monitoring efforts available across Europe.

References:

Jessop, A., Chow, C., Dornelas, M., Pereira, P., Sousa-Pinto, I., Hernández Chan, S., Junker, J., Soares, J., Ratnarajah, L., Fernández, M., Mendo, T. (2022) MarBioME. Overview and assessment of the current state of Biodiversity Monitoring in the European Union and adjacent marine waters. European Commission. Directorate General for Research and Innovation.

Table S19. Marine EBV factsheets: Species abundances of marine commercial fish species and long-distance migratory fishes

**EBV: Species abundances of marine commercial fish species and long-distance migratory fishes**	
ID	24
Realm	Marine
EBV class	Species populations
EBV name	Species abundances of marine commercial fish species and long-distance migratory fishes
Step in identification process	User & Policy Needs Assessment
Definition	The estimated count of individuals of commercially relevant marine fish species and long-distance migratory fishes in EU's marine waters within contiguous spatial units (grid cells) over time.
Metric	- Estimated count of individuals<br>- Modeled relative abundance
Spatial resolution unit	50 × 50 km – 200 × 200 km
Temporal resolution unit	1 year
Taxonomic/ ecosystem focus group	Commercial marine fish species listed in the Common Fisheries Policy

Main European initiatives and description of current bottlenecks

There is not a single monitoring network integrating data of marine commercial fishes across the whole of Europe. However, there are different regional integration initiatives collecting data on the relative abundance of marine fishes that could potentially provide data to generate this EBV. Among them, we highlight here the fish data gathered by the International Council for the Exploitation of the Sea (ICES), corresponding to surveys undertaken through trawls (main source of fish monitoring data in Europe - Jessop et al. 2022). These are mostly directed to commercial species and as such it will prove a good source of data to generate this EBV.

Possible bottlenecks in the generation of this EBV from ICES data relate to:

1) Data collection and sampling. The International Council for the Exploitation of the Sea (ICES) holds a database on pelagic and bottom fish trawl surveys collected in the North-east Atlantic and Baltic seas (DATRAS online database). Survey data comes from standardized survey protocols, but these differ across regions (e.g. between the Baltic and the Western and Southern Areas) (**StMon** partial bottleneck). Data reported to DATRAS includes the age disaggregated abundance of captured fish species that could potentially be used to generate this EBV. The survey data currently covers the Baltic Sea, Skagerrak, Kattegat, North Sea, English Channel, Celtic Sea, Irish Sea, Bay of Biscay and the eastern Atlantic from the Shetlands to Gibraltar; therefore, the geographic coverage of data is not complete as it does not include data from the Mediterranean sea (**GeC** partial bottleneck); this translates in the lack of representation of Mediterranean species in the database (**TxC** partial bottleneck). There are more than 45 years of continuous time series data in DATRAS, and survey data are continuously updated by national institutions (more than once a year in some cases), so the data availability matches the temporal resolution sought by this EBV.

2) Models, interoperability, and IT infrastructure. The ICES does not use models to estimate global and age disaggregated abundance estimates (indices of Catch per Unit Effort) (**Mod** bottleneck). The DATRAS dataset has defined a vocabulary and a set of standards for data reporting to ease data handling, integration, and availability but there are not common metadata standards followed across all surveys (**MtSd** partial bottleneck). Data is centralized and stored in the ICES data portal.

3) Data integration. There is not a single integration initiative that collects and processes data of marine commercial fishes abundance and distributions across Europe (only regional and national ones, being the Mediterranean sea clearly underrepresented) (**EuInt** partial bottleneck). The products generated from DATRAS (i.e. indices of fish stock abundance and estimates of commercial species) do partially match the EBV (DATRAS does not model relative abundance of commercial fish stocks but we could not assess whether this covers all long distance migratory species; **EBVm** partial bottleneck). Since there are not currently models in place to map the distribution of marine fish species, there is no open code or user-friendly software to report here (**OpC** and **SoFw** bottlenecks). Data streams are not fully automated: worksheets in .csv format are submitted to the DATRAS via the ICES platform but there is little information about the level of automatization of the data flows of the underlying datasets (**Auto** partial bottleneck). If funding for adequate survey effort delivering the data exists, it is that of the countries reporting data to DATRAS and ICES (**Fnd** bottleneck). DATRAS data products (such as Catch per area of fishing effort estimates - CPUE- or indices) and raw data, can be freely downloaded according to the ICES Data policy.

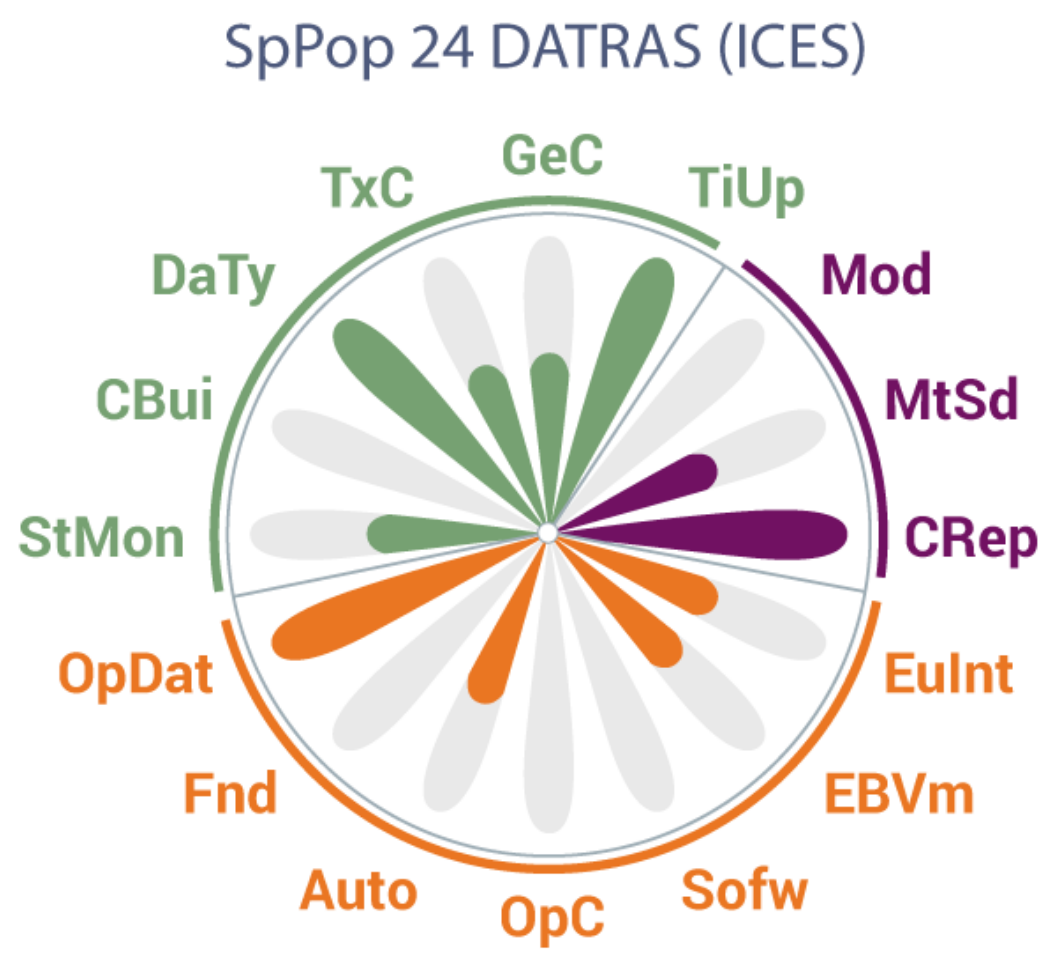

See Jessop et al. (2022) for a summary of other national and regional fish monitoring efforts available across Europe.

References:

Jessop, A., Chow, C., Dornelas, M., Pereira, P., Sousa-Pinto, I., Hernández Chan, S., Junker, J., Soares, J., Ratnarajah, L., Fernández, M., Mendo, T. (2022) MarBioME. Overview and assessment of the current state of Biodiversity Monitoring in the European Union and adjacent marine waters. European Commission. Directorate General for Research and Innovation.

Table S20. Marine EBV factsheets: Species distributions of marine birds

**EBV: Species distributions of marine birds**	
ID	25
Realm	Marine
EBV class	Species populations
EBV name	Species distributions of marine birds
Step in identification process	User & Policy Needs Assessment
Definition	The presence/absence or probability of occurrence of European marine bird species at their breeding sites over time.
Metric	- Binary presence/absence<br>- Probability of occurrence
Spatial resolution unit	10 x 10 km - 50 × 50 km
Temporal resolution unit	3 or 6 years
Taxonomic/ ecosystem focus group	Marine bird species indicated in the Review and analysis of Member States' 2018 reports Descriptor 1: Species biological diversity

Main European initiatives and description of current bottlenecks

There is not a single monitoring network integrating data of marine birds across the whole of Europe. However, there are different regional integration initiatives collecting data on marine bird species presence and abundance across Europe that could potentially provide data to generate this EBV. Among them, we highlight here the OSPAR and HELCOM conventions and the EBBA2. Seatrack community (https://seatrack.net/) is another initiative looking at the North Atlantic that uses tracking data from colonies all over the North Atlantic and generate monthly density maps (https://www.int-res.com/abstracts/meps/v676/meps13854).

The OSPAR convention already collects data on breeding seabird colonies (incl. gulls and terns), breeding waterbirds (incl. waders) and wintering and passage water birds (incl. waders) in countries of the Northeast Atlantic, that gets integrated to generate a Marine Bird Abundance indicator. Similarly, HELCOM generates a Marine Breeding Birds abundance indicator integrating data for 6 marine bird species surveyed across the Baltic Sea. There is currently a joint working group on Marine Birds between OSPAR-HELCOM and the International Council for the Exploration of the Sea (ICES), to enable long-term planning and delivery of significant products across both regions. The European Seabirds At Sea (ESAS) ensembles offshore monitoring data on seabirds (e.g., from ship-based surveys) across the North Sea using a standardized data collection method and complement coastal surveys of breeding and wintering marine birds within the framework of the OSPAR and HELCOM conventions. For example, ESAS data gets integrated to coastal data to generate two indicators "Abundance of waterbirds in the breeding season" and "Abundance of waterbirds in the wintering season".

There is also a newly developed initiative to integrate biodiversity (incl. marine birds) and environmental monitoring at the Mediterranean level but it is still in its preliminary stages. However, this initiative envisages the generation of indicators following those used by the OSPAR commission.

Possible bottlenecks in the generation of this EBV from existing data collected by these networks (OSPAR, HELCOM) relate to:

1) Data collection and sampling. In both OSPAR and HELCOM Marine Birds programs, systematic surveys are currently carried out and funded by national monitoring schemes. Regional/European monitoring standards have not been developed yet, making it difficult data integration at these scales (**StMon** partial bottleneck). HELCOM reports difficulties to integrate data from coastal surveys across countries because of the use of different sampling methods. Data collected are species counts (breeding pairs) but, because the geographic location of colonies sampled is known, this information could be used to generate species distribution maps. OSPAR collects data for up to 100 species, while HELCOM only for 6, so the taxonomic coverage is not complete (**TxC** bottleneck). Geographic coverage is also

incomplete, as on the one hand, these two programs focus on North-East Atlantic and Baltic countries, leaving the Mediterranean Sea completely uncovered; on the other hand, the OSPAR Marine Bird program reports data gaps for some countries which have limited seabird colony monitoring, or which are not contributing any data to the regional indicator (**GeC** bottleneck). Capacity building (if exist) is that of national monitoring schemes (**CBui** bottleneck).

2) <u>Models, interoperability and IT infrastructure</u>. These initiatives do not use models to generate spatially explicit estimates of species distributions (**Mod** bottleneck). The Marine Bird Abundance indicator integrates species trends (as estimated from the software TRIM). Each contributing country has its own data Quality Assurance and Quality Control Protocols, with European standards to be developed (**MtSd** partial bottleneck). Similarly, each contributing country has its own storage mechanism. Ultimately, data is stored in the OSPAR and HELCOM Marine Bird databases hosted by ICES Data Centre via the ICES Biodiversity Data Portal (this data however, is currently not available at ICES) (**CRep** partial bottleneck).

3) <u>Data integration</u>. There is not a single integration initiative that collects and processes data of marine birds' abundance and distributions across Europe (only regional ones, with the Mediterranean Sea being clearly underrepresented) (**EUInt** partial bottleneck). Products from OSPAR and HELCOM (indicators of abundance trends) do not match the specifications of this EBV (**EBVm** bottleneck). As there are not currently models in place to map the distribution of marine bird species, there is no open code or user-friendly software to report here (**OpC** and **SoFw** bottlenecks). Data streams are not automated with, for example, OSPAR data being submitted to the coordination node via written procedure into a standard set of Excel worksheets (**Auto** bottleneck). Currently, funding for adequate survey effort delivering the data basis needed for OSPAR and HELCOM indicator work (as well as other requirements by conservation directives) is still lacking in several countries (**Fnd** bottleneck). Raw data is currently not available (**OpDat** bottleneck).

The **Second European Breeding Bird Atlas (EBBA2)** (Keller et al. 2020) mapped the distribution of 63 marine and coastal species at 50 x 50 km resolution for the 2013-2017 period (distribution of nesting places on land), but it also presents bottlenecks when it comes to generating this EBV including insufficient temporal and spatial resolution (see a detailed description of bottlenecks regarding the generation of EBV *Species distributions of all birds* in Table S27).

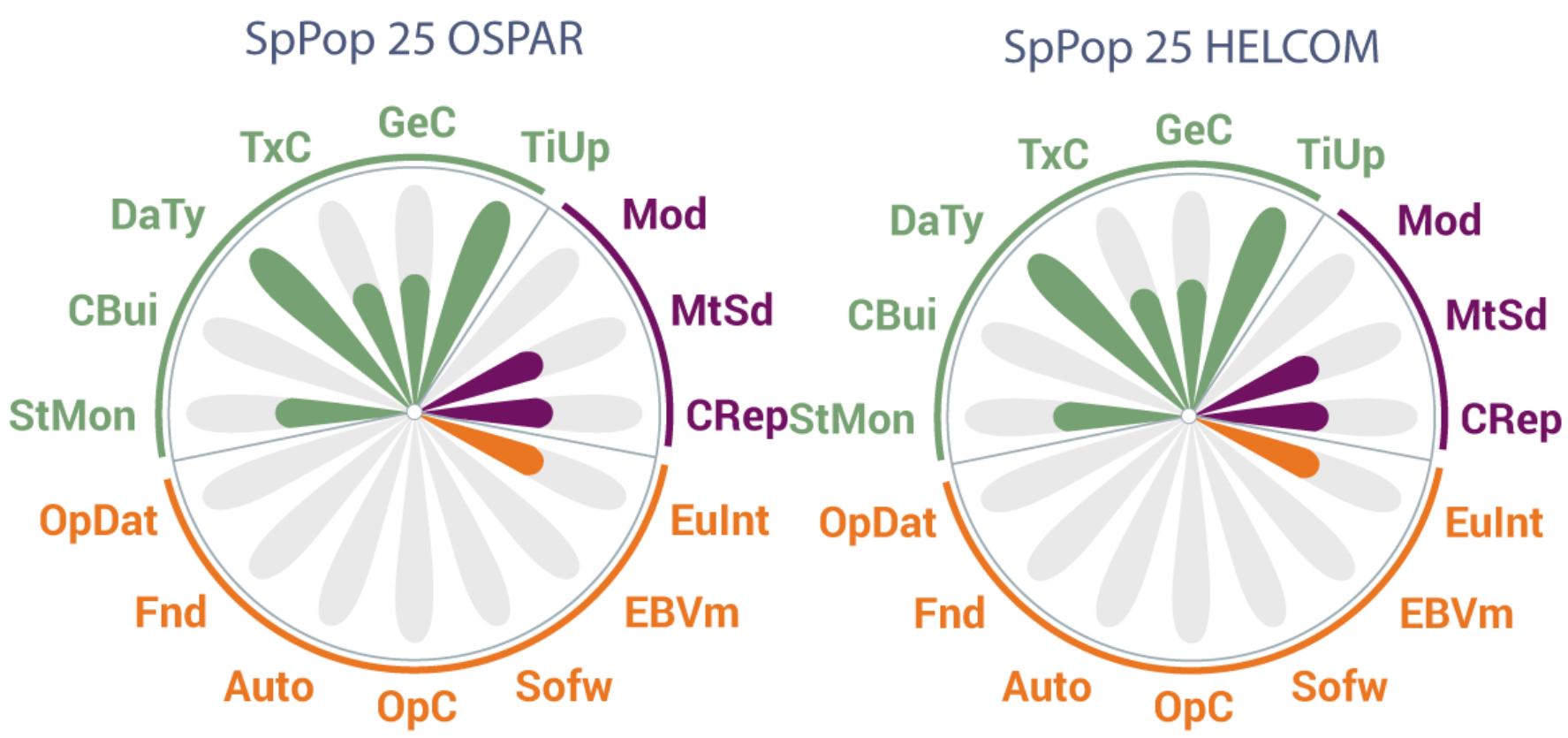

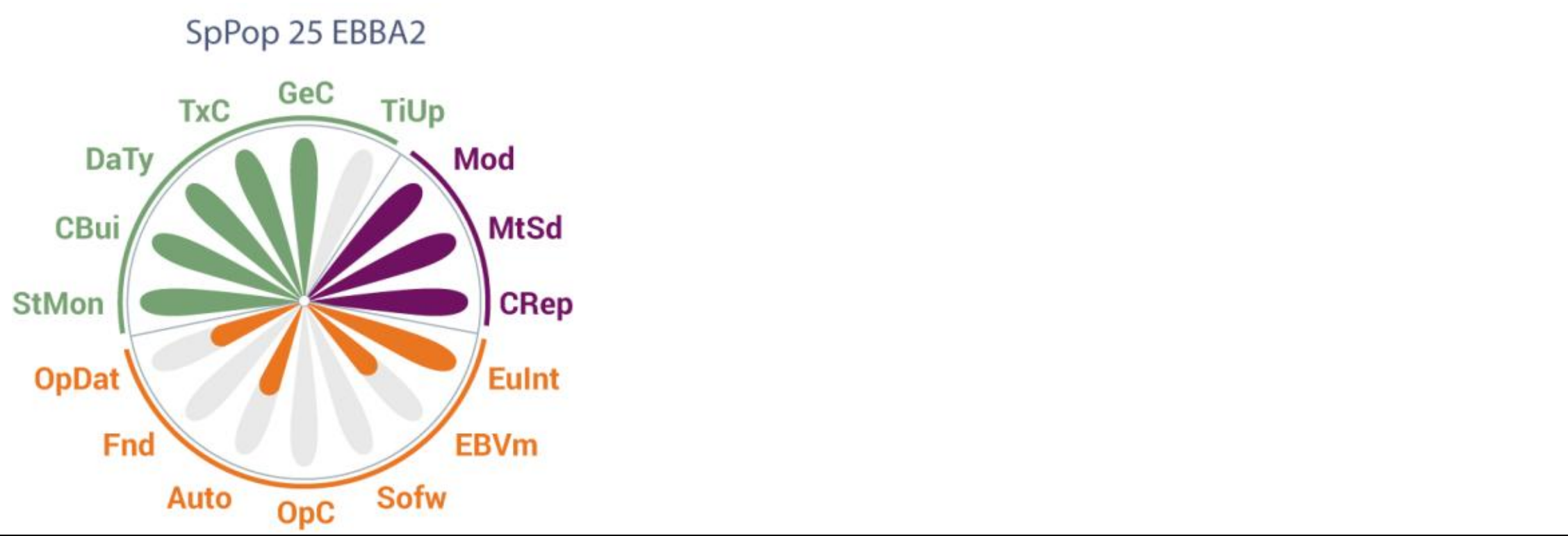
SpPop 25 EBBA2
GeC
TiUp
Mod
MtSd
CRep
EuInt
EBVm
Sofw
OpC
Auto
Fnd
OpDat
StMon
CBui
DaTy
TxC

Table S21. Marine EBV factsheets: Species distributions of marine mammals

**EBV: Species distributions of marine mammals**	
ID	26
Realm	Marine
EBV class	Species populations
EBV name	Species distributions of marine mammals
Step in identification process	User & Policy Needs Assessment
Definition	The presence/absence or probability of occurrence of European marine mammal species within contiguous spatial units (grid cells) in EU's marine waters over time.
Metric	- Binary presence/absence<br>- Probability of occurrence
Spatial resolution unit	10 x 10 km - 50 × 50 km
Temporal resolution unit	3 or 6 years
Taxonomic/ ecosystem focus group	Marine mammal species indicated in the Review and analysis of Member States' 2018 reports Descriptor 1: Species biological diversity

Main European initiatives and description of current bottlenecks

There is not a single monitoring network integrating data of marine mammals across the whole of Europe. For cetaceans, there are currently not even internationally coordinated monitoring schemes at the relevant spatial scales needed for the assessment of these wide-ranging species. The available integration initiatives collecting data on marine mammal species presence and abundance data are national or regional (e.g. OSPAR - North-East Atlantic or HELCOM - Baltic Sea) and generally focus on a single or a reduced set of species, making it difficult to assess the overall gaps and bottlenecks regarding the monitoring of this taxonomic group across European seas (but see Jessop et al. 2022 for a review of monitoring gaps of marine mammals across all scales).

For example, the abundance and distribution of cetaceans is one of the agreed common OSPAR indicators (M4), integrating data from different large-scale international surveys such as SCANS (Small Cetaceans in European Atlantic waters and the North Sea) and CODA (Cetacean Offshore Distribution and Abundance in the European Atlantic) plus other unknown number of smaller-scale surveys. It estimates species trends and generates maps of predicted abundances across the North-East Atlantic (modelled density surfaces). Therefore, data used for the generation of M4 indicators could potentially serve the generation of this EBV, but only for a limited number of species. Moreover, OSPAR recognizes there is a general lack of data to fulfil the six-yearly reporting requirements under the Habitats Directive and the Marine Strategy Framework Directive for most cetacean species/area units (also the temporal resolution sought for this EBV), since the frequency of these large-scale surveys is > 10 years.

Another OSPAR indicator on marine mammals is the indicator on *Seal abundance and distributio*n (M3). This indicator integrates estimates of seal numbers from monitoring programmes that count seals on land when they are moulting or breeding and are run on a regular basis. The frequency and timing of seal surveys varies among OSPAR Contracting Parties and take place during one or more seal key life stages, such as moulting or breeding.
The monitoring is conducted in the Great North Sea and the UK part of the Celtic Sea, so its geographic coverage is very restricted. A similar seal monitoring is carried out across the Baltic Sea under the umbrella of HELCOM convention; data collected gets integrated to estimate population trends and abundance of seals and seals' distribution maps across the Baltic.

In the Mediterranean region we highlight the 'Fixed Line Transect Mediterranean monitoring Network' that coordinates a continuous monitoring of mega and macro marine fauna (cetaceans, sea turtles, seabirds, and other macro marine fauna) since 2007. However, data from this network has not been integrated yet, for example, to generate reference indicators across the Mediterranean Sea. These

data have been used to investigate species presence and distribution for conservation purposes, habitat use, long-term trends, correlation with environmental features and influence of the main threats of maritime traffic and marine litter. All these studies have for the moment been restricted to scientific publications.

References:

Jessop, A., Chow, C., Dornelas, M., Pereira, P., Sousa-Pinto, I., Hernández Chan, S., Junker, J., Soares, J., Ratnarajah, L., Fernández, M., Mendo, T. (2022) MarBioME. Overview and assessment of the current state of Biodiversity Monitoring in the European Union and adjacent marine waters. European Commission. Directorate General for Research and Innovation.

Table S22. Marine EBV factsheets: Distributions of marine turtle species nesting grounds

**EBV: Distributions of marine turtle species nesting grounds**	
ID	27
Realm	Marine
EBV class	Species populations
EBV name	Distributions of marine turtle species nesting grounds
Step in identification process	User & Policy Needs Assessment
Definition	The presence/absence or probability of occurrence of marine turtle species nesting ground in the EU's coastline over time.
Metric	- Binary presence/absence<br>- Probability of occurrence
Spatial resolution unit	10 x 10 km - 50 × 50 km
Temporal resolution unit	3 or 6 years
Taxonomic/ ecosystem focus group	Turtle species indicated in the Habitats Directive and the Review and analysis of Member States' 2018 reports Descriptor 1: Species biological diversity: *Caretta caretta* (Loggerhead turtle), *Chelonia mydas* (Green turtle), *Dermochelys coriacea* (Leatherback turtle), *Eretmochelys imbricata* (Hawksbill turtle), *Lepidochelys kempii* (Kemp's Ridley turtle)

Main European initiatives and description of current bottlenecks

There is not a single initiative integrating data on marine turtle nesting sites across Europe. There are however a few regional short- term regional or national initiatives monitoring turtle nesting sites such as those framed within the EuroTurtles or the MedTurtle LIFE projects; moreover, guidelines have been developed by the UNEP to standardize to the maximum extent monitoring protocols for nesting areas of *Careta careta* and *Chelonia mydas* across the Mediterranean, but to what extent this methodology has been applied it is unknown.

In the MarBioMe review, Jessop et al. (2022) found sea turtles to be among the marine taxonomic groups less monitored across Europe with most sea turtle monitoring programmes being conducted by the Republic of Ireland (i.e. large Geographic Coverage gaps) and/or focused on recording offshore observations rather than nesting grounds (e.g., the 'Fixed Line Transect Mediterranean monitoring Network').

The lack of information on exactly what these monitoring programs are, what is the temporal resolution of data collection or to what extent data gets shared and integrated among them, complicates the evaluation of the current bottlenecks on data flows for the generation of this EBV at a European level.

References:

Jessop, A., Chow, C., Dornelas, M., Pereira, P., Sousa-Pinto, I., Hernández Chan, S., Junker, J., Soares, J., Ratnarajah, L., Fernández, M., Mendo, T. (2022) MarBioME. Overview and assessment of the current state of Biodiversity Monitoring in the European Union and adjacent marine waters. European Commission. Directorate General for Research and Innovation.

Table S23. Marine EBV factsheets: Species distributions of invasive alien marine taxa of European concern

**EBV: Species distributions of invasive alien marine taxa of European concern**	
ID	29
Realm	Marine
EBV class	Species Populations
EBV name	Species distributions of invasive alien marine taxa of European concern
Step in identification process	User & Policy Needs Assessment
Definition	The presence/absence or probability of occurrence of invasive freshwater species (as specified in the Consolidated List of Invasive Alien Species of Union Concern) in the EU's marine waters within contiguous spatial units (grid cells) over time.
Metric	- Binary presence/absence<br>- Probability of occurrence
Spatial resolution unit	1 x 1 km - 10 x 10 km
Temporal resolution unit	3 or 6 years
Taxonomic/ ecosystem focus group	Marine species as specified in the Commission Implementing Regulation 2016/ 1141, 2017/1263, 2019/1262, 2022/1203, 2025/1422 Consolidated list at: https://eur-lex.europa.eu/legal-content/EN/TXT/PDF/?uri=CELEX:02016R1141-20220802&from=EN

Main European initiatives and description of current bottlenecks

The European Alien Species Information Network (EASIN) is an integration initiative coordinated at the European level (European Commission Joint Research Centre) that aggregates, integrates, and harmonizes spatio-temporal data for alien species (AS) and Invasive Alien Species (IAS) across Europe. The species catalogue and geodatabase are regularly updated through literature reviews, data partners inputs, and the official reports by Member States competent authorities. The EASIN Catalogue (29.07.2025) includes 1,441 marine alien species, of which 5 are IAS of Union concern (IAS UC) (EU Regulation 1143/2014). The Catalogue's protocol provides information on the data collected for each species. EASIN facilitates the exploration of data and information from existing monitoring networks and programs available from a variety of distributed information sources by providing tools and interoperable web services, compliant with internationally recognized standards.

Possible bottlenecks in the generation of this EBV from existing data collected by EASIN data relate to:

1) Data collection and sampling. EASIN carries out the systematic collection of data on IAS in Europe, does not come exclusively from monitoring programs but from a variety of sources, including literature review, occasional observations and data portals (**StyMon** partial bottleneck); EASIN has demonstrated capacity building through different activities including: i) ad hoc assessments, such as the Baseline Distribution of IAS of Union concern; ii) support to the establishment of surveillance systems in compliance with EU AS policy; iii) citizen science programs, (e.g. the development of the smartphone app 'IAS in Europe' freely available and already adopted in the context of several projects and for official monitoring; iv) training to teachers and students (e.g., the MOOC course "Have you seen an alien?"), among others. EASIN collaborates with the Member States competent authorities and national experts to update and validate the datasets relevant in the EU policy context, to fulfil the EU mandate of setting up national monitoring networks for IAS; however, the extent to which this has been implemented is not fully known. For example, a recent European evaluation on the reporting of non-indigenous species under the Marine Strategy Framework Directive (Tsiamis et al. 2021) showed that only 8 Member States reported information regarding the abundance and spatial distribution of invasive alien species and only 3 MS reported information of the impacts of these species on natural habitats and species groups. Some bottlenecks regarding data collection by MS may relate to 1) lack of communication between national coordinators

& EU funded projects working on IAS in a country (e.g. LIFE projects; Magliozzi et al. 2024); 2) at the national level, people in charge of monitoring AS belong to different admin bodies among which there is little communication and coordination and 3) complex administrative structure in some countries (federal, regions, etc.) and how the environmental competences are distributed among them, making it difficult to harmonise and coordinate data collection tasks and to facilitate data flows and integration. The EASIN Geo Database (04.08.2025) contains occurrence records for more than 14,347 alien, across 64different countries (EU & beyond) (including data for the 114species in the consolidated list of IAS of Union concern- European Union (EU) Regulation 1143/2014). Because the data is georeferenced, it can be used to build species distribution models. The EASIN Geodatabase is updated on average 3 times/ year, but some information sources are updated less regularly, depending on effort for the update and the periodicity of their own updates. Recent EASIN database backend developments data updating will facilitate more regular updates.

2) Models, interoperability, and IT infrastructure. EASIN is not a modelling framework, therefore there is no direct access to modelling workflows (**Mod** partially bottleneck). A case study using habitat suitability models was carried out for the freshwater invasive species *Elodea nuttalli* using EASIN data across the EU (Steen et al. 2019). There is, however, regular in-house research work on IAS of Union concern to evaluate the range of expansion at both European and national levels, to understand whether niche of the species is at equilibrium in its invaded range or to predict the probability of occurrence of the species in Europe considering bioclimatic variables. EASIN makes data available under the CC-BY licence enabling reuse with attribution to the original sources through web tools for data analysis, open sharing and reporting. The openness of this data is key to redirect surveillance and trigger early warning systems. While standards have been defined to harmonize data across source databases, these do not necessarily apply to the underlying data (**MtSd** partial bottleneck).

3) Data integration. Data collected by EASIN are at different resolutions and aggregated at e.g., country, Marine Region, 10 x 10 km grid cells, and partially matches the EBV. The EASIN Geodatabase follows a protocol to regularly retrieve data from the data partners; data are subsequently transformed and converted through the following steps: validation, cleansing and standardization, geocoding, mapping, application of quality rules and finally loaded on the Geodatabase (Datawarehouse). The EASIN Geodatabase also integrates data from the "IAS Europe'' smartphone App, which promotes the report of sightings of IAS of Union Concern by citizens, and data from the NOTSYS platform. The latter is the official tool for EU Member States to notify the Commission and inform the other Member States as required by Reg. 1143/2014 on IAS. In particular, the tool has been designed to facilitate a timely comprehensive notification of new detections of IAS of Union concern, related eradication measures, and their effectiveness. Funding is a bottleneck to the generation of this EBV: while the European Commission has set up EASIN as the official information system supporting Member States in the implementation of the Regulation (EU) No 1143/2014 , it only co-participate in the expenses of monitoring or eradication actions by national competent authorities, funding from the European Commission for monitoring of IAS is available via existing financial instruments such as LIFE, H2020, Cohesion or Regional Development funds. Funding is warrant in the mid - long - term to maintain the EASIN data infrastructure and the development of new data collection tools (e.g., a web base platform for reporting citizen science data) (**Fnd** partial bottleneck).

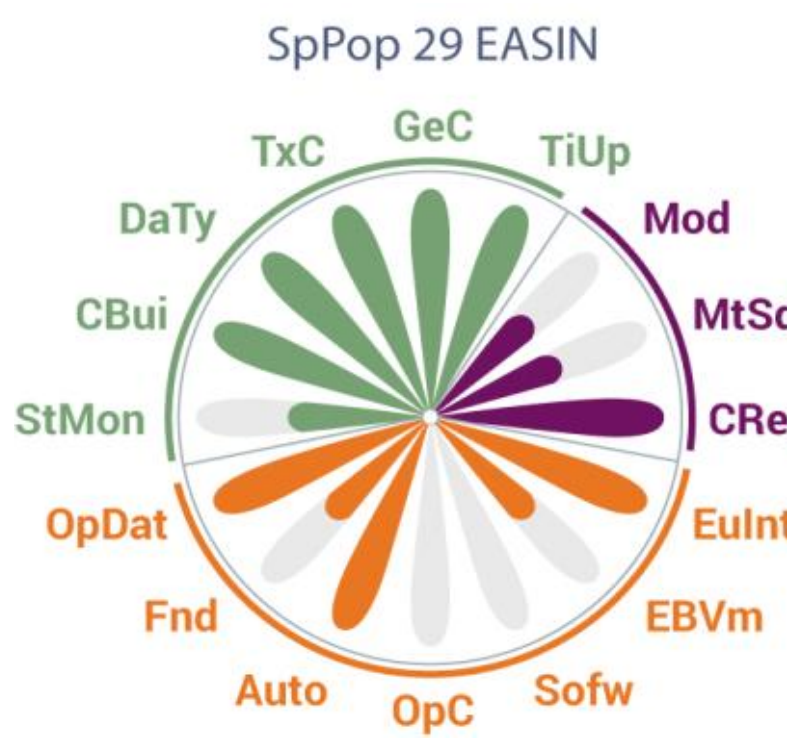

References:
Magliozzi C, Lucrezi S, Oficialdegui FJ, Gervasini E, Melone B, Cardoso AC (2024) Transnational

cooperation in EU: opportunities for addressing invasive alien species. Management of Biological Invasions 15(4): 683–700, https://doi.org/10.3391/mbi.2024.15.4.13

Steen, B, Cristina Cardoso, A.C., Tsiamis, K., Nieto, K.,Engel J. and Gervasini, E. (2019) Modelling hot spot areas for the invasive alien plant Elodea nuttallii in the EU. *Management of Biological Invasions* 10(1): 151–170. URL .

Tsiamis, K., Boschetti, S., Palialexis, A., Somma, F. and Cardoso, A.C. (2021) Marine Strategy Framework Directive - Review and analysis of EU Member States' 2018 reports - Descriptor 2: Non-indigenous species; Assessment (Art. 8), Good Environmental Status (Art. 9) and Targets (Art. 10), EUR 30520 EN, Publications Office of the European Union, Luxembourg, ISBN 978-92-79-27700-2, doi:10.2760/7897, JRC123179. URL.

Table S24. Marine EBV factsheets: Ecosystem distribution of hard coral habitats

**EBV: Ecosystem distribution of hard coral habitats**	
ID	32
Realm	Marine
EBV class	Ecosystem structure
EBV name	Ecosystem distribution of hard coral habitats
Step in identification process	Internal review process
Definition	Presence/absence or probability of occurrence of hard corals habitats in EU's marine waters within contiguous spatial units (grid cells) over time.
Metric	- Binary presence/absence<br>- Probability of occurrence
Spatial resolution unit	10 x 10 m - 300 x 300 m
Temporal resolution unit	3 or 6 years
Taxonomic/ ecosystem focus group	Live hard coral covers defined by EMODnet Seabed Habitats

Table S25. Marine EBV factsheets: Ecosystem distribution of macroalgae canopy cover

**EBV: Ecosystem distribution of macroalgae canopy cover**	
ID	33
Realm	Marine
EBV class	Ecosystem structure
EBV name	Ecosystem distribution of macroalgae canopy cover
Step in identification process	Internal review process
Definition	The estimated percentage of macroalgae canopy cover in EU's marine waters within contiguous spatial units (grid cells) over time.
Metric	Estimated canopy cover
Spatial resolution unit	10 x 10 m - 300 x 300 m
Temporal resolution unit	3 or 6 years
Taxonomic/ ecosystem focus group	Macroalgae canopy covers defined by EMODnet Seabed Habitats

Table S26. Marine EBV factsheets: Ecosystem distribution of seagrass habitats

**EBV: Ecosystem distribution of seagrass habitats**	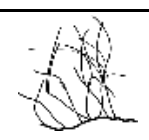
ID	34
Realm	Marine
EBV class	Ecosystem structure
EBV name	Ecosystem distribution of seagrass habitats
Step in identification process	Internal review process
Definition	Presence/absence or probability of occurrence of seagrass habitats in EU's marine waters within contiguous spatial units (grid cells) over time.
Metric	- Binary presence/absence<br>- Probability of occurrence
Spatial resolution unit	10 x 10 m - 300 x 300 m
Temporal resolution unit	3 or 6 years
Taxonomic/ ecosystem focus group	Seagrass covers defined by EMODnet Seabed Habitats

The EBVs *Ecosystem distribution of hard coral habitats (Table S24), Ecosystem distribution of marine macroalgae canopy cover (Table S25),* and *Ecosystem distribution of marine seagrass habitats (Table S26)*, are also defined as Essential Ocean Variables (EOV), and their distribution around Europe has been recently mapped by EMODnet (vector format) for the Atlas of Marine Life in Europe. The generation of distribution maps of these three EBVs is framed within the EMODnet broad-scale seabed habitat map for Europe (EUSeaMap). Maps were derived from a compilation of different data sources used including data from the Habitats Directive reporting, specific habitat locations collected in different monitoring programs (e.g. maps of threatened and/or declining habitats hard corals developed by the OSPAR convention) and others. The EuSeaMap project is under continuous development and improvement, and to date has been updated three times 2012, 2016, 2021. If the mapping of these habitats will continue the same frequency of update, these EMODnet products will match the temporal resolution sought by these EBVs. The resolution of the EuSeaMap and the habitat maps is of 100 x 100 m, so, within the range of the spatial resolution defined for this EBV. Given the diversity of data sources and methods involved in the mapping of these habitats, it is difficult to make a thorough assessment of the bottlenecks in data flows to the generation of these EBVs as it has been done for other EBVs.

Terrestrial species populations

Table S27. Terrestrial EBV factsheets: Species distributions terrestrial birds

**EBV: Species distributions terrestrial birds**	
ID	44
Realm	Terrestrial
EBV class	Species populations
EBV name	Species distributions of terrestrial birds
Step in identification process	User & Policy Needs Assessment
Definition	The presence/absence or probability of occurrence of each European terrestrial bird species within contiguous spatial units (grid cells) during the breeding season across the EU over time.
Metric	- Binary presence/absence during breeding season<br>- Probability of occurrence during breeding season
Spatial resolution unit	1 × 1 km – 10 × 10 km
Temporal resolution unit	3 or 6 years
Taxonomic/ ecosystem focus group	All terrestrial birds of the EU (taxonomy based on the HBW and BirdLife Taxonomic Checklist, with focus on those bird species that are officially recognized in the List of birds of the European Union.

Main European initiatives and description of current bottlenecks

The **Second European Breeding Bird Atlas (EBBA2)** (Keller et al. 2020) is the most recent and comprehensive European-wide integration initiative mapping the species distributions of all European terrestrial birds. The EBBA2 has mapped the distribution, abundance and breeding likelihood of 596 breeding birds (all native and non-native breeding species) across Europe at the 50 x 50 km grid resolution. For this purpose, an extensive collection of data from multiple sources was conducted at national level and then integrated at European level. Moreover, species distribution maps at 10 km resolution (probability of occurrence) were developed for 222 species using statistical modelling.

Possible bottlenecks in the generation of this EBV from existing data collected by the EBBA network relate to:

1) Data collection and sampling: EBBA2 targeted surveys (breeding period; 10 km^2) followed a standardized protocol (time surveys 60 – 120 min, carried out between 2013 – 2017) which derived from different ongoing monitoring programs depending on each country (data from national atlases, national breeding surveys, etc.). Thanks to external funding (by the MAVA foundation and others, as there was no EU funding in support of this initiative), the EBBA2 project contributed to capacity building in different countries; however, it will be difficult to replicate the sampling intensity and effort dedicated to EBBA2 with a frequency lower than 20-30 years for all species (**TiUp** bottleneck); however, and because of the joint work between EuropaBON and EBCC, species distributions for a set of 50 European bird species (in particular farmland species) have been generated to test the capacities and limitations to update maps and analyse change with a frequency of 5 years (EBBA Live Farmland initiative https://ebba2.info/live/farmland/).

2) Models, Interoperability and IT infrastructure: there are no bottlenecks to report in this regard: the EBBA2 used models to map the distribution of bird species (probability of occurrence), designed metadata standards for data reporting and aggregation, and developed tools to harmonize and centralize data at the European level.

3) Data integration: while EBBA2 has already generated continuous predictions of the distributions of all birds across Europe, EBBA2 products only partially match the definition of this EBV (with divergences in taxonomic completeness, temporal and spatial resolution

demanded – only 222 species were modelled at 10 km resolution and none at a finer resolution; **EBVm** partial bottleneck). The 10 x 10 km grid species distribution models generated by the project were fit using R (a priori, a non-user-friendly software), and the code used to fit the models is not openly available (**OpC** and **Soft** bottlenecks). The models used to generate 10 x 10 km species distribution maps in EBBA2 (probability of occurrence) were complex (especially the site occupancy models) and require specific data from the observation process not always available because of the type of surveys carried out; there is a bottleneck due to the need of advanced technical programming skills and model knowledge to model species distributions. The EBBA2 was supported by external funding (the biggest donor being the MAVA foundation); the funding was directed to training, capacity building and bird sampling in countries with lower monitoring capacities, but it also supported the overall coordination of the EBBA2 project. This funding support ended with the publication of the Atlas, but the EBCC started in 2023 a small grant fund to try to ensure the continuity of bird monitoring and atlas programs in some countries/regions and data integration tasks. Funding, however, is limited (based partially on EBBA2 book royalties) and unsecure (**Fnd** bottleneck). While EBBA2 has made a huge effort to automate data flows (e.g., data aggregation at both the national and European levels has been automated through the use of MapViewers), the automatization of data flows from sampling plots to European coordinators was lacking (**Auto** partial bottleneck). All the data generated in EBBA2 is available either open access (50 x 50 observed occurrence data) or upon request (10 x 10 km modelled probability of occurrence data, 50 x 50 km breeding evidence data, 50 x 50 km abundance data and 50 x 50 km EBBA1 – EBBA2 change data). In the latter case, requests should be approved by an EBCC committee and data handling fees can be charged. EBBA2 50 x 50 occurrence data are available under license CC BY 4.0, the rest of EBBA2 maps are © EBCC. Following EBCC data policy, raw 10 x 10 km sampling data is not owned by the EBCC but by its national partners, which could be asked from them (**OpDat** partial bottleneck).

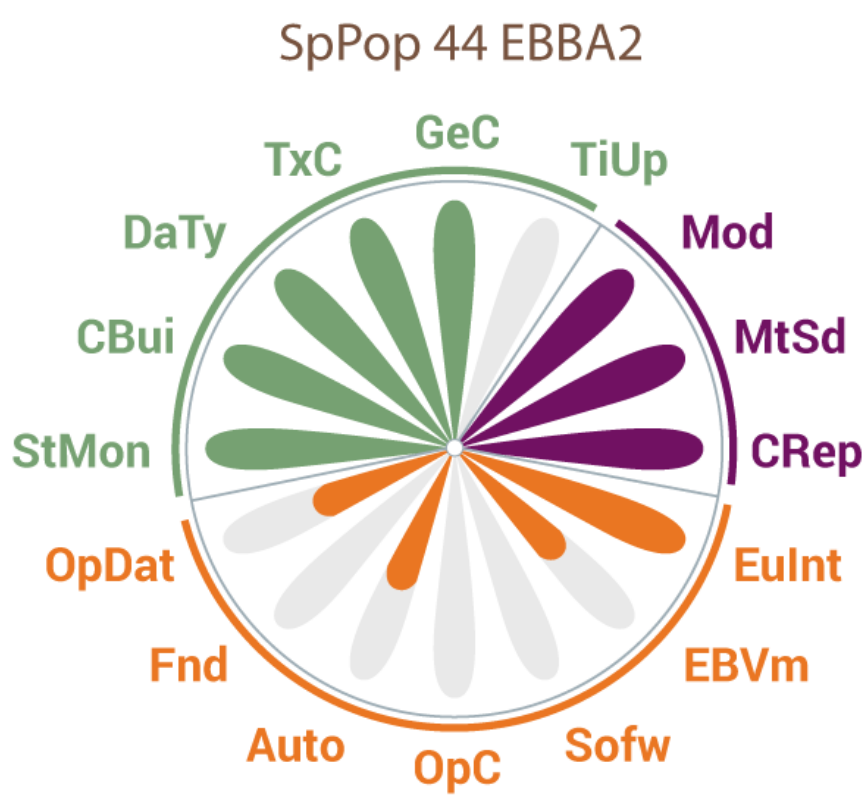

There are other European-coordinated initiatives collecting bird data in a systematic manner and on a yearly basis like the Pan-European Common Bird Monitoring Scheme (PECBMS), but the taxonomic coverage of this initiative (common birds, 170 bird species) so far falls short of achieving the mapping of all terrestrial birds across Europe (see a detailed description of this integration initiative in the EBV fact sheet below “Species abundances of terrestrial common birds”, in Table S28).

The EuroBirdPortal (EBP) also compiles data on species distributions and breeding evidence at 10 km resolution across Europe on a daily basis, though data is mostly unstructured and semi-structured (see a detailed description of this integration initiative in the EBV fact sheet below “Species abundances of terrestrial migratory bird species” in Table S30). The main bottleneck in this case is that, currently, the number of species covered by this integration initiative is low (137 bird species in total). The EU-funded project “LIFE EBP reinforcement" (LIFE22-PLP-ES-EBP-reinforcement) attempts to enlarge the number of species and fully cover all bird species found in Europe by 2026.

On the other hand, the EBBA Live Farmland initiative, aims at combining the PECBMS and EBP datasets to produce maps of breeding distribution at a high temporal resolution (< 5 years), using the modelling experience gained by EBBA2.

References:

Keller, V., Herrando, S., Voríšek, P., Franch, M., Kipson, M., Milanesi, P., ... and Foppen, R. P. B. (2020). European Breeding Bird Atlas 2: Distribution, abundance and change. European Bird Census Council and Lynx Editions, 967 pp.

Table S28. Terrestrial EBV factsheets: Species Abundances of Common Birds

**EBV: Species Abundances Terrestrial Birds: COMMON BIRDS**	
ID	45 (a)
Realm	Terrestrial
EBV class	Species Populations
EBV name	Species abundances of terrestrial common birds
Step in identification process	User & Policy Needs Assessment
Definition	The estimated count of individuals of European common bird species within contiguous spatial units (grid cells) across the EU over time
Metric	- Estimated count of individuals<br>- Modelled relative abundance
Spatial resolution unit	1 x 1 km, 10 x 10 km
Temporal resolution unit	1 year
Taxonomic/ ecosystem focus group	- Taxonomy based on the Handbook Birds of the World (HBW) and BirdLife Taxonomic Checklist<br>- Common bird species as included in the Pan-European Common Bird Monitoring Scheme (PECBMS)

Main European initiatives and description of current bottlenecks

The **Pan-European Common Bird Monitoring Scheme (PECBMS)** integrates data on species occurrences and abundance of common birds across Europe following standardized methods. It generates species trends and multi-species trends indicators at the European level and on an annual basis. It also produces indicators for the four main European bioregions. All EU countries are included in the project. The data type collected in these standardized monitoring schemes could potentially be used to generate this EBV (spatially explicit abundances across Europe) but have not been used yet within the framework of this initiative for that purpose. PECBMS only covers the breeding season, but some of the national common bird monitoring schemes participating in the project also collect data during winter.

Possible bottlenecks in the generation of this EBV from existing data collected by the PECBMS network relate to:

1) Data collection and sampling: PECBMS collects count data for all common bird species across Europe following standardized sampling protocols and on a seasonal basis (breeding counts) at a very fine spatial resolution (line-transects, point counts and territory mapping); while PECBMS sampling sites are distributed across all European countries the degree of coverage in some countries is very low (especially in South and East European countries). Regular training for the network of national coordinators is provided by PECBMS (capacity building and training). Therefore, there are no bottlenecks to report regarding data collection for this monitoring initiative in relation to the generation of this EBV.

2) Models, Interoperability and IT infrastructure: The PECBMS integration initiative has demonstrated the capacity to use models to generate single- and multi-species indicator trends at the European level (TRIM model: TRends and Indices for Monitoring data). However, the program has not explored yet the use of species abundance models to obtain continuous spatially explicit estimates of species abundances across Europe. The potential of using this type of surveys for mapping has been explored in scientific publications, but not within the framework of the initiative (**Mod** partial bottleneck). PECBMS follows metadata standards to facilitate data harmonization and flows and counts on a central repository, curated by the Czech Society for Ornithology (CSO**).**

3) Data integration: There is a mismatch between the EBV description and its spatial resolution (1 x 1 – 10 x 10 km) and the products generated by this integration initiative (species trends

reported at national and European level on an annual basis; **EBVm** bottleneck). The current software used for estimating population trends is R software, but it has been made user-friendly open to everyone through an R package (RTRIM-shell). This same package cannot be used to map species abundances(**OpC** and **Sofw** bottlenecks). Data flows from national coordinators to the European integration node (the Czech Society for Ornithology) are standardized national coordinators submit national species trends to an online platform using standardized formats and the system performs several automatic checks. Additional manual checks are carried out by the European coordination team; data integration at the national level is automatized in some cases, but data collection still relies on field observations not always collected using IT tools (**Auto** partial bottleneck). The PECBMS integration initiative has been supported by the European Commission since 2002 (3-year tender) but funding stability in the mid-long- term is not warranted; the same applies to both the national integration nodes and the survey programs (**Fnd** partial bottleneck). Raw data is only available upon request and subject to agreement by national coordinators who hold the ownership of data (**OpDat** partial bottleneck), but the PECBMS central coordination team has a database with all the annual counts per site and year (PECBMS site-level data) and only one centralized data request should be done (authorizations by national owners are coordinated by PECBMS).

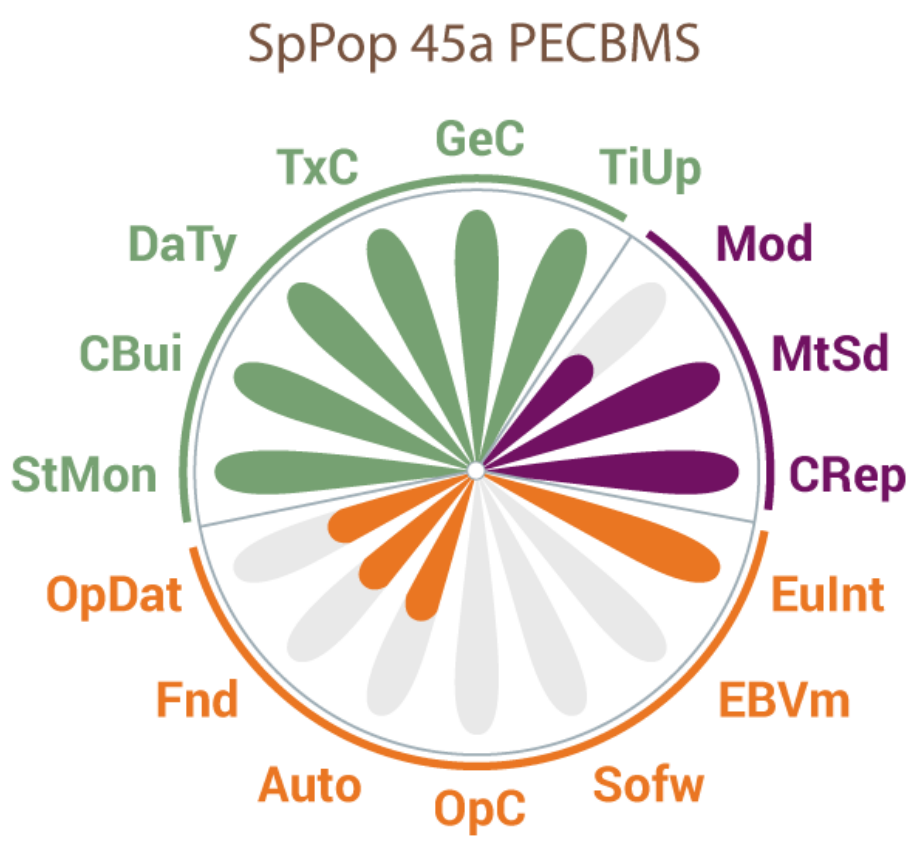

The EuroBirdPortal (EBP) also compiles data on species occurrence at 10 km resolution across Europe on a daily basis (see a detailed description of this integration initiative in the EBV fact sheet below "Species abundances of terrestrial migratory bird species"). The EBP data, though mostly consisting of unstructured and semi-structured data, could also be used to produce models of relative abundance (e.g., in combination with PECBMS data). The number of common bird species covered by this integration initiative is, currently, relatively low but it is expected to be completed in 2026 (see EBBA2). For example, EBBA Live Farmland initiative (https://ebba2.info/live/farmland/), combines the PECBMS and EBP datasets to produce maps of breeding distribution of farmland birds at a high temporal resolution (< 5 years).

Table S29. Terrestrial EBV factsheets: Species abundances of priority and rare birds

**EBV: Species abundances terrestrial birds: PRIORITY AND RARE BIRDS**	
ID	45 (a)
Realm	Terrestrial
EBV class	Species Populations
EBV name	Species abundances of terrestrial priority and rare birds
Step in identification process	User & Policy Needs Assessment
Definition	The estimated count of individuals of European rare and priority bird species within contiguous spatial units (grid cells) across the EU over time.
Metric	- Estimated count of individuals<br>- Modelled relative abundance
Spatial resolution unit	1 x 1 km, 10 x 10 km
Temporal resolution unit	1 year
Taxonomic/ ecosystem focus group	Taxonomy based on the HBW and BirdLife Taxonomic Checklist<br>- Rare bird species as included in the Annex I of the Birds Directive (i.e., (Art.4) species with small populations or restricted local distribution)<br>- Priority bird species as included in Annex I of the Birds Directive (i.e., in danger of extinction, vulnerable to specific changes in their habitat or requiring particular attention for reasons of the specific nature of the habitat)

Main European initiatives and description of current bottlenecks

The **Second European Breeding Bird Atlas (EBBA2)** (Keller et al. 2020) is the most recent and comprehensive European-wide integration initiative mapping the species distributions of all European terrestrial birds. The EBBA2 has mapped the distribution, abundance and breeding likelihood of 596 breeding birds (both native and non-native) across Europe at the 50 x 50 km grid resolution. For this purpose, an extensive collection of data from multiple sources was conducted at national level and then integrated at European level. Moreover, species distribution maps at 10 km resolution (probability of occurrence) were developed for 222 species using statistical modelling.

Possible bottlenecks in the generation of this EBV from existing data collected by the EBBA network relate to the same as in EBV 44 (see fact sheet “Species distributions terrestrial birds”, Table S27).

Birdlife international compiles and regularly updates data on bird populations at the European scale for the *Red List of European Birds* and as such, it could represent an interesting data source for the generation of this EBV (two publications to date in 2015 and 2021). This Red List estimates the regional extinction risk of all species of birds occurring regularly and naturally in Europe. This assessment is especially relevant for rare and priority species and has a special focus on bird conservation and environmental legislation. Data sources used for this assessment include data reported by the 28 Member States of the European Union (EU) under Article 12 of the EU Birds Directive, national reports from 22 countries and territories in Europe which are not part of the EU, and additional data sources (e.g. data from the European Atlas of Breeding Birds and other bibliographic sources). The main bottleneck here is that data compiled (mostly number of breeding pairs of a given species at the national country) are not spatially explicit and therefore could not be used to generate this EBV.

The EuroBird Portal (EBP) also compiles data on bird species at 10 km resolution across Europe on a daily basis (see a detailed description of this integration initiative in the EBV fact sheet below “Species abundances of terrestrial migratory bird species”, Table S30). The main bottleneck in this case is that, currently, the number of species covered by this integration initiatives is low (137 bird species in total) and only some priority and rare species are included.

The Pan-European Common Bird Monitoring Scheme (PECBMS) collects bird data in a systematic manner and on a yearly basis but as it is focussed on common birds species it could only provide data for a few species (see a detailed description of this integration initiative in the EBV 45(a) fact sheet "Species abundances of terrestrial common birds", in Table S28).

References:

Keller, V., Herrando, S., Voříšek, P., Franch, M., Kipson, M., Milanesi, P., ... and Foppen, R. P. B. (2020). European Breeding Bird Atlas 2: Distribution, abundance and change. European Bird Census Council and Lynx Editions, 967 pp.

Table S30. Terrestrial EBV factsheets: Migratory birds

**EBV: Species Abundances Terrestrial Birds: MIGRATORY SPECIES**	
ID	45 (b)
Realm	Terrestrial
EBV class	Species Populations
EBV name	Species abundances of terrestrial migratory bird species
Step in identification process	User & Policy Needs Assessment
Definition	The estimated count of individuals of European migratory bird species within contiguous spatial units (grid cells) across the EU over time.
Metric	- Estimated count of individuals<br>- Modeled relative abundance
Spatial resolution unit	1 x 1 km, 10 x 10 km
Temporal resolution unit	Real-time
Taxonomic/ ecosystem focus group	- Taxonomy based on the HBW and BirdLife Taxonomic Checklist<br>- Migratory bird species defined as full migrants in the European Red List

Main European initiatives and description of current bottlenecks

The **EuroBirdPortal (EBP)** is an integration initiative that can currently produce distribution maps for migratory species during the migratory periods, but also for other seasons (e.g. winter), as the data is collected year-round. The data collected by the EBP has already been used to generate the kind of metrics required for this EBV (spatially explicit abundances across Europe; cf. Gargallo et al. 2022) for a few species but this process has not been automated yet nor protocolized for that purpose within the framework of the EBP.

Possible bottlenecks in the generation of this EBV from existing data collected by the EBP initiative relate to:

1) Data collection & sampling. The EBP has the potential to collect data on all bird species occurring in Europe (including migratory species). Currently, only the data for 137 species is stored in the EBP central data repository (**TxC** partial bottleneck), but the current EU- funded project (EU project 101104367 — LIFE22-PLP-ES-EBP reinforcement) attempts to enlarge the number of species and fully cover all bird species found in Europe by 2026. Data is collected across all European countries, but the degree of coverage is poorer in the South and, particularly, in the East and South-East. Data (species observations & counts) are gathered from online bird recording portals (e.g., ornitho) and include, essentially, both casual observations and data collected following simple systematic protocols (species lists). Only a small part of the data is collected following standardized monitoring protocols (**StMon** partial bottleneck). Training and capacity building for the network is provided during regular annual meetings or direct support to specific EBP partners. Data is updated daily with information up to the previous day, which for the purpose of the current EBV could be considered nearly as in real time.

2) Models, data interoperability and IT infrastructure. Although EBP data has already been used to produce weekly models of relative abundance at the necessary spatial resolution for a few species, the process has not been automated yet as an EBP data product nor extended to all migratory species (**Mod** partial bottleneck). The EBP follows metadata standards to ensure data harmonization among its different data sources. The EBP data is harmonized, managed, and stored in a central repository curated by the European Bird Census Council (EBCC).

3) Data Integration. The maps featured in the EBP viewer (www.eurobirdportal.org) are aggregated by week and 30 x 30 km but in the EBP central data repository the casual data is aggregated at 10 x 10 km and date and the lists are in raw format (i.e. not aggregated) with

date/timing and location given as precise location or at 10 x 10 km; therefore, the EBP maps partially match the specifications of this EBV (**EBVm** partial bottleneck: data available would not allow the mapping of species abundances at 1 x 1 km resolution). Though this integration initiative has already produced models of species relative abundances over space, these are not routinely done to generate EBP products and therefore there is no open code nor user-friendly software yet to report in this regard (**Sofw** and **OpC** bottlenecks). Data streams are automated: the local online portals collect most of their data through mobile apps in near-real time or shortly after it has been recorded in the field; data collected in data portals is then automatically transferred to the EBP daily. The EBP main developments have been possible thanks to the support of the LIFE programme, but funding stability in the mid-long-term is not warranted (**Fnd** bottleneck). EBP data is available upon request and subject to agreement by national coordinators who hold the ownership of data (**OpDat** partial bottleneck), but just one centralized data request should be done as data is already centralized in the EBP databank (authorizations by national owners are coordinated by EBP).

SpPop 45b EPB

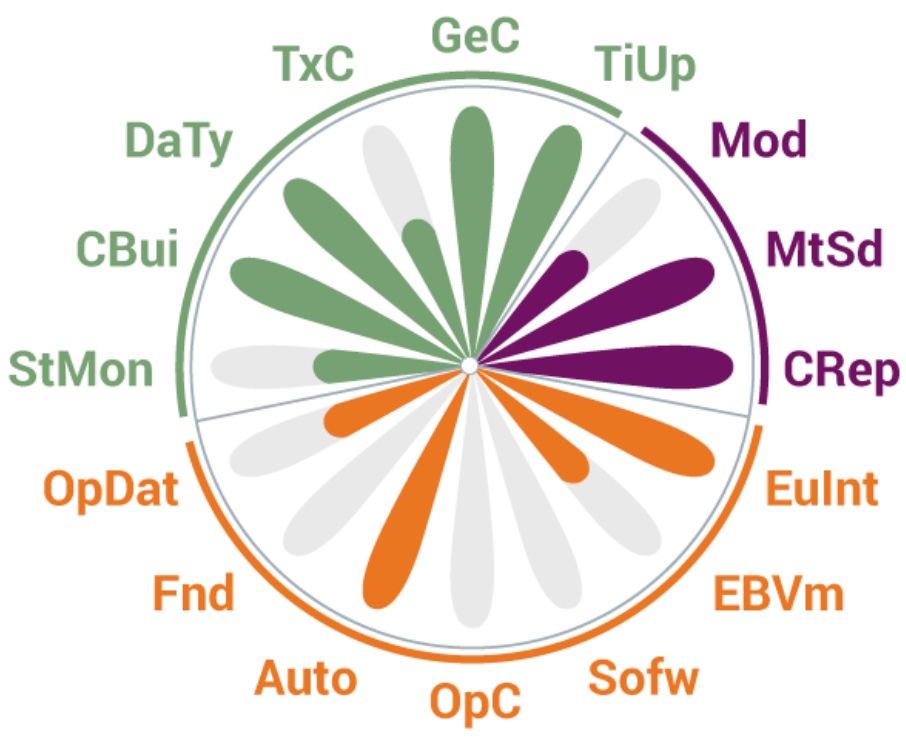

References:

Gargallo, G., Davies, J.G., Faverjon, C., Kampichler C., Baillie, S.R., Cameron, A., Robinson, R.A. and Sierdsema, H. (2022) Development of a prototype early warning system for avian influenza in the EU based on risk-mapping. EFSA supporting publication 2022: EN-7762, 46pp. URL.

Table S31. Terrestrial EBV factsheets: selected terrestrial mammals

**EBV: Species abundance of selected terrestrial mammals**	
ID	46
Realm	Terrestrial
EBV class	Species Populations
EBV name	Species abundance of selected terrestrial mammals
Step in identification process	User & Policy Needs Assessment
Definition	The estimated count of individuals of European terrestrial Carnivora, Artiodactyla and Chiroptera species within contiguous spatial units (grid cells) across the EU over time.
Metric	- Estimated count of individuals<br>- Modelled relative abundance<br>- Estimated counts of individuals in key underground sites (hibernation, breeding and transitional roost-sites) as defined by EUROBATS<br>- Estimated counts of individuals in key overground sites (hibernation, breeding and transitional roost-sites) as defined by EUROBATS
Spatial resolution unit	1 x 1 km - 10 x 10 km
Temporal resolution unit	1 year
Taxonomic/ ecosystem focus group	Terrestrial Carnivora, Artiodactyla and Chiroptera species included in the European Red List.

Main European initiatives and description of current bottlenecks

The Atlas of European Mammals is the first coordinated initiative that has attempted to map the distribution of all mammals at 50 x 50 km resolution across Europe. The First Atlas of European Mammals was published in 1999 (Mitchell-Jones et al. 1999, and the Second Atlas of European Mammals (EMMA2) is expected to be released in 2026. These initiatives, however, do not provide information on mammal abundance (or at least not as square-based data), only distribution, and therefore, it cannot be used to generate this EBV.

The information from mammals is very scattered across Europe, mostly collected at national, subnational or Natura 2000 levels and with few truly European initiatives (at least compared to other taxa such as birds and butterflies).

For the generation of this EBV it is noteworthy to mention emerging integration initiatives such as the European Observatory of Wildlife (EOW) or the Bat Monitoring Programme, specifically for chiroptera, amongst others.

The EOW (a project by ENETWILD) aims to enhance collaboration among the stakeholders that monitor, conserve, and manage wildlife in Europe (with a special focus on mammals). It seeks to develop a framework where data will be comparable, interoperable, and openly accessed at the European level, providing guidelines for monitoring mammals in Europe (e.g., protocols to estimate wildlife density), training (e.g., on new tools for data processing and analysis) and compiling and harmonize existing databases on mammals. Data gathered through this portal following standardized monitoring protocols could eventually be used to obtain spatially-explicit estimates of mammal abundances/densities across Europe.

The Bat Monitoring Programme's main goal is to compile bat-related scientific data collected by either researchers or naturalists (citizen science), to be used as an indicator of the general state of nature (https://www.bats.org.uk/our-work/national-bat-monitoring-programme). This new platform aims to use large-scale and long-term standardized monitoring data on changes in bat populations, and has been designed to be user-friendly and open, and it is already totally functional for any region across

Europe. This programme has developed four standard protocols specifically designed for monitoring a certain group of bat species, either forest-dwelling, cave-dwelling or urban-dwelling species (ChiroRivers, ChiroHabitats, ChiroBoxes and ChiroRoosts) using different techniques (bioacoustics, underground and overground roosts surveys, visual censuses or bat boxes), although it also accepts data from capture sessions as well as opportunistic sights. The combination of all monitoring protocols provides a complete image of the distribution and trends of many bat species populations (qualitative and quantitatively). Given data collection follows standardized protocols, it will be easy to harmonize it across countries and be potentially used for modelling the distribution of bats across Europe and generating this EBV. This monitoring programme has kicked off in Catalonia and is now being implemented at the Spanish scale, with plans to be expanded in all of Europe in the near future. The Bat Monitoring Programme's efforts are well-aligned with the Bat Monitoring and Surveillance Intersessional Working Group in the EUROBATS Commission. This group has worked on gathering data collected via systematic protocols by national surveillance groups, along with other data sources (e.g. literature review) to develop a prototype indicator of trends in European bat populations, as well as updated bat monitoring guidelines. These data get reported to EUROBATS (key underground and overground sites) by countries on a voluntary basis alongside the reporting to the Habitats Directive.

References:

Mitchell-Jones et al. (Eds) 1999 The Atlas of European Mammals. *Academic press*, p 484

Table S32. Terrestrial EBV factsheets: all terrestrial mammals

**EBV: Species distributions of all terrestrial mammals**	
ID	47
Realm	Terrestrial
EBV class	Species Populations
EBV name	Species distributions of all terrestrial mammals
Step in identification process	User & Policy Needs Assessment
Definition	The presence/absence or probability of occurrence of all European terrestrial mammal species within contiguous spatial units (grid cells) across the EU over time.
Metric	- Binary presence/absence<br>- Probability of occurrence
Spatial resolution unit	10 x 10 km - 50 x 50 km
Temporal resolution unit	3 or 6 years
Taxonomic/ ecosystem focus group	European terrestrial mammal species included in the European Red List.

Main European initiatives and description of current bottlenecks

The Atlas of European Mammals is the first coordinated initiative that has attempted to map the distribution of all mammals at 50 x 50 km resolution across Europe. The First Atlas of European Mammals was published in 1999 (Mitchell-Jones et al. 1999) but the Second Atlas of European Mammals (EMMA2) is expected to be released in 2026.

Possible bottlenecks in the generation of this EBV from existing data collected by EMMA2 data relate to:

1) Data collection and sampling. Most of the data used for the EMMA2 compilation will not come exclusively from systematic monitoring programs (**StMon** partial bottleneck). Data sources include national and regional databases, literature records and data portals. While the products generated by this initiative are binary (presence/absence of species within each 50 x 50 km grid cell), data contributors should report the number of individuals observed (counts); that raw data could potentially be used to make spatially-explicit predictions of species to unsampled areas, although probably not at 10 x 10 km resolution (**DaTy** partial bottleneck). The integration initiative does not have capacity building (if it exists, it depends on national initiatives) (**Cbui** bottleneck). The EMMA2 gathers data from 21 countries and the coverage and the quality of data is uneven across them, so gaps of information are expected particularly in some areas (**GeC** partial bottleneck). The spatial resolution of EMMA2 maps does not match the finer spatial resolution sought in this EBV (10 x 10 km) ; moreover, the temporal resolution (frequency) of atlas updates is too low (> 10 years) (**TiUp** bottleneck), therefore the products generated by this integration initiative do not match the EBV definition (**EBVm** bottleneck in *Data Integration*).

2) Models, interoperability, and IT infrastructure. EMMA2 maps will reflect the confirmed presence of the species aggregated at 50 x 50 km and there is no modelling involved in predicting species distributions across areas not covered by data (**Mod** bottlenecks). Metadata standards are defined (with the intention of following Darwin Core) and data get integrated into a central repository by the European Mammal Foundation.
3) Data integration. Products generated by EMMA2 are maps of species distributions at 50 x 50 km resolution and as such they partially match the definition of this EBV; however, the frequency of atlas updates is too low (> 10 years) and it does not match the temporal resolution sought for this EBV (**EVBm** partial bottleneck). Currently, there are no models in place to map the distribution of terrestrial mammals across Europe within the framework of the EMMA2, therefore, there is no open code or user-friendly software to report here (**OpC** and **SoFw** bottlenecks). While metadata standards are defined, data flows are not automated

(**Auto** bottleneck): records are to be collected into CSV files or Access database (or similar) and submitted via email to coordinators who harmonize and integrate the data from the different sources. Funding has been secured for the publication of the Atlas but there is no funding to support the long-term continuation of this initiative (**Fnd** bottleneck). The EMMA2 original records will be held in a system that is accessible to researchers, with appropriate safeguards for sensitive records (rare species, vulnerable habitats or other reasons), though the great majority of records will be freely accessible at a higher resolution than in the Atlas, for example in national or regional atlases (**OpDat** partial bottleneck).

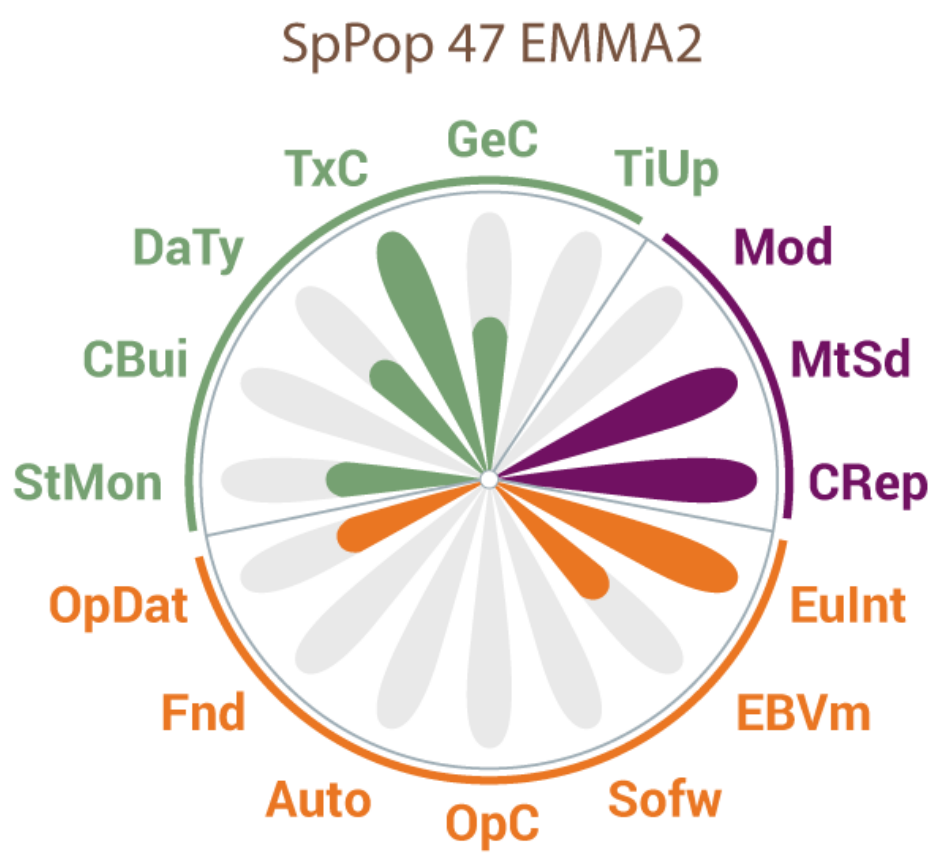

Also noteworthy to the generation of this EBV are emerging integration initiatives such as the European Observatory of Wildlife (EOW) or the Bat Monitoring Programme.

The EOW (a project by ENETWILD) aims to enhance collaboration among the stakeholders that monitor, conserve, and manage wildlife in Europe (with a special focus on mammals). It seeks to develop a framework where data will be comparable, interoperable, and openly accessed at the European level, providing guidelines for monitoring mammals in Europe (e.g., protocols to estimate wildlife density), training (e.g., on new tools for data processing and analysis) and compiling and harmonize existing databases on mammals. Data gathered through this portal following standardized monitoring protocols could eventually be used to obtain spatially-explicit estimates of mammal distributions across Europe.

The Bat Monitoring Programme's main goal is to use bats as indicators of the general state of nature using large-scale and long-term monitoring data on changes in bat populations across Europe. This programme has developed four standard protocols specifically designed for monitoring a certain group of bat species, either forest-dwelling, cave-dwelling or urban-dwelling species (ChiroRivers, ChiroHAbitats, ChiroBoxes and ChiroRoosts). The combination of all four monitoring programmes provides a complete image of the health status of all bat species populations. Data gets collected by professionals and volunteers (citizen science). Given data collection follows standardized protocols, it will be easy to harmonize it across countries and be potentially used for modelling the distribution of bats across Europe and generating this EBV. This monitoring programme has kicked off in Catalonia and is now being implemented at the Spanish scale, with plans to be expanded in all of Europe in the near future. The Bat Monitoring Programme's efforts are well-aligned with the Bat Monitoring and Surveillance Intersessional Working Group in the EUROBATS Commission. This group has worked on gathering data collected via systematic protocols by national surveillance groups, along with other data sources (e.g. literature review) to develop a prototype indicator of trends in European bat populations. These data get reported to EUROBATS (key underground and overground sites) by countries on a voluntary basis alongside the reporting to the Habitats Directive.

References:

Mitchell-Jones et al. (Eds) 1999 The Atlas of European Mammals. *Academic press*, p 484.

Table S33. Terrestrial EBV factsheets: terrestrial reptiles

**EBV: Species distributions of terrestrial reptiles**	
ID	48
Realm	Terrestrial
EBV class	Species Populations
EBV name	Species distributions of terrestrial reptiles
Step in identification process	User & Policy Needs Assessment
Definition	The presence/absence or probability of occurrence of all European terrestrial reptile species within contiguous spatial units (grid cells) across the EU over time.
Metric	- Binary presence/absence<br>- Probability of occurrence
Spatial resolution unit	1 x 1 km - 10 x 10 km
Temporal resolution unit	3 or 6 years
Taxonomic/ ecosystem focus group	European terrestrial reptile species included in the European Red List.

Main European initiatives and description of current bottlenecks

The New Atlas of Amphibians and Reptiles of Europe (NA2RE, 2014) is the most updated Europe-wide coordinated initiative that has attempted to map the distribution of reptiles across the continent at 50 x 50 km grid. It generated maps for 218 taxa (it covers 145 species of reptiles) updated as of 2014 (no information about temporal dynamics).

Possible bottlenecks in the generation of this EBV from data flows in NA2RE relate to:

1) Data collection and sampling. Most of the data used for the Atlas compilation did not come from systematic monitoring programs (**StMon** partial bottleneck). Data sources included: (1) data published (in books or websites) or ongoing national atlases, (2) personal data kindly provided to the *Societas Europaea Herpetologica*, (3) the 1997 European Atlas, and (4) the Global Information Facility (GBIF). While data is available across most European countries (23; i.e., it does fulfil the criteria set to qualify for full Geographic Coverage in this assessment), the data coverage within each country was uneven and very scarce in many cases, with lack of funding and personnel being identified as limiting factors to set up national databases in many countries. The initiative did not have capacity building (if it existed, it was that of the national nodes) (**CBui** bottleneck). The Atlas is a snapshot of the distribution of amphibians and reptiles and therefore, does not match the temporal resolution criteria of this EBV (**TiUp** bottleneck).

2) Models, interoperability, and IT infrastructure. Maps in NA2RE reflect an aggregation of the raw data at 50 x 50 km and there is no modelling involved in predicting species distributions across areas not covered by data (**Mod** bottleneck) or at finer resolutions (e.g., 10 x 10 km). Data from different sources (collected using different standards) were centralized and harmonized for the atlas (**MtSd** bottleneck); the authors of the atlas recognize the challenge of finding better ways to gather species occurrence data across Europe and centralized it (**CRep** bottleneck) (Sillero et al. 2014).

Data integration. The maps generated by this integration initiative do not fully match the EBV definition (they do not fulfil the criteria of spatial and temporal resolution; **EBVm** partial bottleneck). The NA2RE initiative did not use models to map the distribution of reptiles, therefore, there is no open code or user-friendly software to report here (**OpC** and **SoFw** bottlenecks). Data streams were not automated (**Auto** bottleneck). Funding supported the publication of the Atlas, but it is not available in the long-term to generate this EBV with the temporal frequency needed (**Fnd** bottleneck). The NA2RE maps are freely available for download but the raw data are not (**OpDat** partial bottleneck).

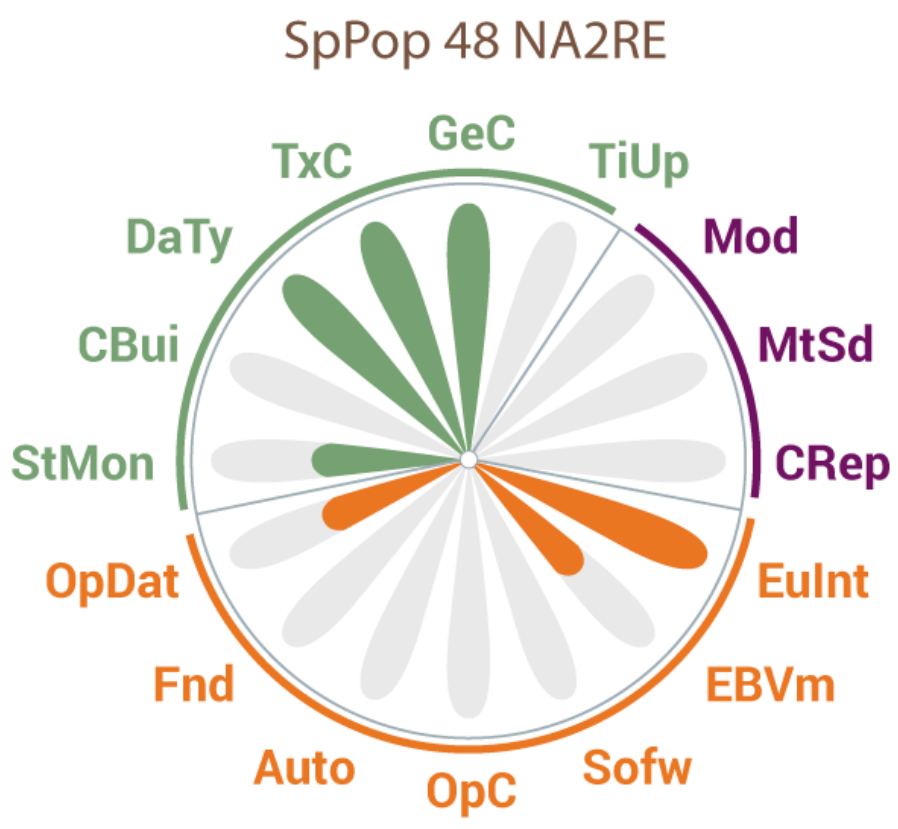

Another integration initiatives at the European level retrieving information on reptile distributions are the IUCN European Red Lists of Reptiles. However, most of the time IUCN maps are not linked to monitoring programs (but expert based) and represent a snapshot of the species distributions, so this initiative could nott contribute to the quantification of this EBV at the desired spatio-temporal scale.

References:

Sillero, N., Campos, J., Bonardi, A., Corti, C., Creemers, R., Crochet, P. A., ... and Vences, M. (2014). Updated distribution and biogeography of amphibians and reptiles of Europe. *Amphibia-reptilia*, 35(1), 1-31.

Table S34. Terrestrial EBV factsheets: Species abundance of butterflies

EBV: Species abundance of butterflies

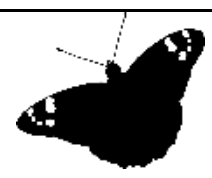

ID	49
Realm	Terrestrial
EBV class	Species Populations
EBV name	Species abundance of butterflies
Step in identification process	User & Policy Needs Assessment
Definition	The estimated count of individuals of butterfly species within contiguous spatial units (grid cells) across the EU over time.
Metric	- Estimated count of individuals of grassland butterfly species<br>- Modelled relative abundance of grassland butterfly species
Spatial resolution unit	10 x 10 km - 50 x 50 km
Temporal resolution unit	1 year
Taxonomic/ ecosystem focus group	Current list of butterfly species underlying the European grassland butterfly indicator, with extension to butterfly species from other habitats.

Main European initiatives and description of current bottlenecks

The **European Butterfly Monitoring Scheme (eBMS)** collects abundance data for > 312 butterfly species, and in addition collects data on moths, a few bumblebees and dragonflies. The abundance data collected (counts per transect) are mostly integrated to estimate population trends at the national and European levels (six indicators: grassland, forests, N2000, climate change, widespread species, and urban butterflies).

Possible bottlenecks in the generation of this EBV from existing data collected by the eBMS data relate to:

1) Data collection and sampling. There are no bottlenecks to report in this regard to the generation of this EBV. eBMS focuses its efforts mostly on the collection of butterflies and moths' abundance data using systematic transect counts that are visited several times per year (the spatio-temporal resolution of raw data is high and could potentially be used to generate this EBV). The geographic coverage of this monitoring program has recently been enlarged thanks to the support of a service contract from the European Union Directorate General for the Environment (ABLE project), so it currently covers 30 countries. The program promotes capacity building through the free publication of informative material for the identification of butterflies in different languages and countries, the offering of open training courses on the use of modelling tools for species trend estimations, the development of Apps to ease data collection and identification, etc.

2) Models, interoperability, and IT infrastructure. This initiative uses the rbms package to estimate population trends and calculation of trend indices/products at the national level (training courses and materials are available on the eBMS website). There is no model proposal from the network to calculate species abundance across the EU over time (**Mod** bottleneck). The eBMS follows metadata standards that allow harmonization of the data collected across the different European countries. The eBMS database is stored and managed in a centralized repository under the custody of the Butterfly Conservation Europe and the UK Center for Ecology and Hydrology.

3) Data Integration. Currently, there is not match between the products generated by this initiative (grassland indicator; trends at the national and European level) and the EBV

definition (**EBVm** bottleneck). The current software used for calculating butterfly trends, indexes, and indicators is R (*rbms* package; non-user-friendly software; **SoFw** bottleneck). However, the code is openly shared and training is provided to users of eBMS data (workshop contents are available on the eBMS website) and GitHub (https://github.com/RetoSchmucki/rbms). While this same package cannot be used to map species distributions, it could be expected that if this initiative were to derive spatially explicit estimates of species occurrences, it would do so by relying on the same software and open code. However, currently, there is no open code for spatially explicit abundance models within the framework of the eBMS program (**OpC** bottleneck). eBMS has recently developed a mobile application (eBMS App) that allows volunteers to record species observations and abundances and directly upload them to the eBMS database (also to review the uploaded data). However, data collected (species lists, tables with data observations) also get reported via email to eBMS coordinators, so data streams are not fully automated (**Auto** partial bottleneck). Funding for coordination and integration tasks is not secured at both the European and national levels; for example, the last geographic expansion of the eBMS network was possible thanks to the ABLE project, funded by the European Union Directorate General for the Environment, for a period of two years from 2018-2020 (**Fnd** bottlenecks). Data is only available upon request (**OpDat** partial bottleneck).

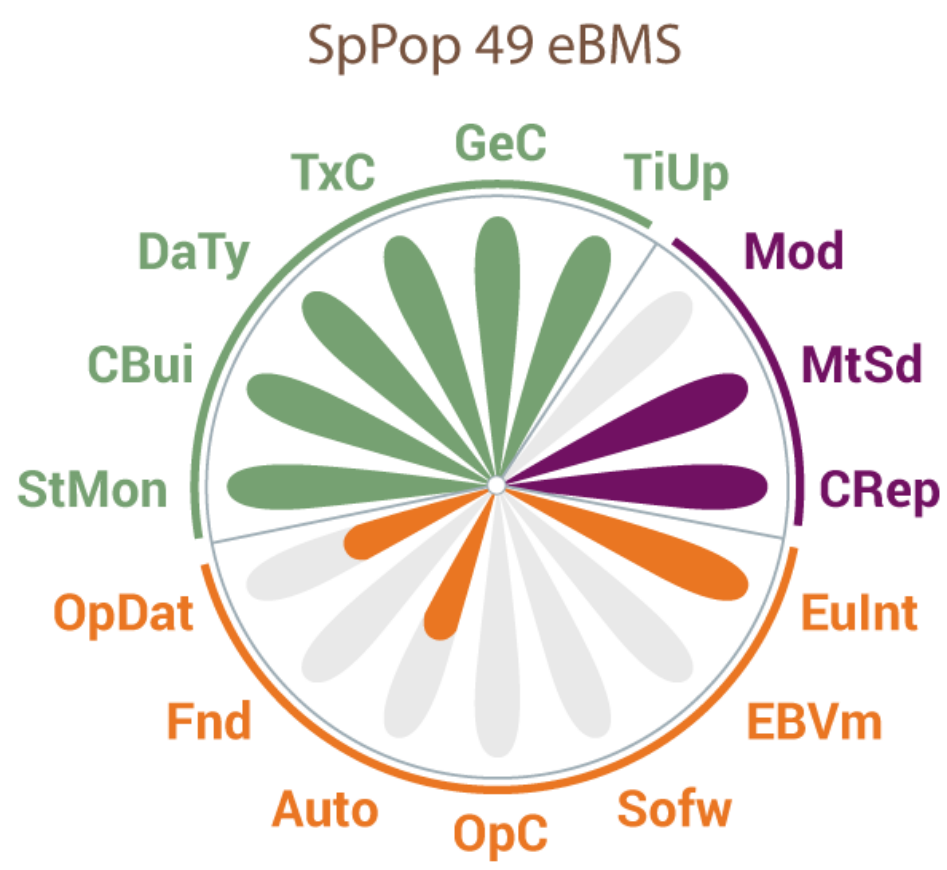

Table S35. Terrestrial EBV factsheets: Species distributions of priority invertebrates and key pollinators

**EBV: Species distributions of terrestrial priority invertebrates and key pollinators**	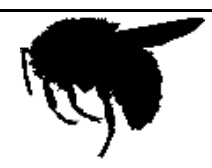
ID	50
Realm	Terrestrial
EBV class	Species Populations
EBV name	Species distributions of terrestrial priority invertebrates and key pollinators
Step in identification process	Internal review process
Definition	The presence/absence or probability of occurrence of priority invertebrates and key pollinator species within contiguous spatial units (grid cells) across the EU over time.
Metric	- Binary presence/absence<br>- Probability of occurrence
Spatial resolution unit	10 x 10 km - 50 x 50 km
Temporal resolution unit	3 or 6 years
Taxonomic/ ecosystem focus group	- Priority invertebrates as listed in the Annex II and Annex IV of the Habitats Directive<br>- Key pollinator species as specified by the EU Pollinator Monitoring Scheme (EU PoMS)

Main European initiatives and description of current bottlenecks

Here we describe two European-wide integration initiatives that could provide data for the generation of species distribution maps of key pollinators: The **European Butterfly Monitoring Scheme (eBMS)** and the **European Pollinator Monitoring scheme (EU PoMS).**

The **European Butterfly Monitoring Scheme (eBMS)** collects abundance data for > 312 butterfly species and numerous moth species. It also collects data of other pollinator species (e.g. bumblebees) but only in a small proportion of the transects and the quality of the data is highly variable. The abundance data collected (counts per transect) gets mostly integrated to estimate population trends at the national and European levels (six indicators: grassland, forests, N2000, climate change, widespread species and urban butterflies).

Possible bottlenecks in the generation of this EBV from existing data collected by the eBMS data relate to:

1) Data collection and sampling. The eBMS focuses its efforts mostly in the collection of butterflies and moths' abundance data using systematic transect counts that are visited several times per year (the resolution of raw data is high and could potentially be used to generate this EBV: species distributions from transect counts/occurrences). However, these two taxonomic groups represent only a small fraction of all European pollinators (lack of taxonomic completeness) (**TxC** bottleneck). The geographic coverage of this monitoring program has recently been enlarged thanks to the support of a service contract from the European Union Directorate General for the Environment (ABLE project), so it currently covers 30 countries. The program promotes capacity building through the free publication of divulgation informative material for the identification of butterflies in different languages and countries, the offering of open training courses on the use of modelling tools for species trend estimations, the development of Apps to ease data collection and identification, etc.

2) Models, interoperability, and IT infrastructure. This initiative uses the rbms package to estimate population trends and calculation of trend indices/products at the national level (training courses and materials are available on the eBMS website); however, there is not a

model proposal from the network for the calculation of trends in distribution. Occupancy models are being used for this within this project but these are complex and not user friendly (**Mod** bottleneck). The eBMS follows metadata standards that allow harmonization of the data collected across the different European countries. The eBMS database is stored and managed in a centralized repository under the custody of the Butterfly Conservation Europe and the UK Center for Ecology and Hydrology.

3) Data Integration. There is not a perfect match between the products generated by this initiative (indicator trends at the national and European level) and the EBV definition (in terms of product and spatial resolution 10 x 10 km, 50 x 50 km) (**EBVm** bottleneck). The current software used for calculating butterfly trends, indexes and indicators is R (*rbms* package; non-user-friendly software; **SoFw** bottleneck). However, the code is openly shared and training is provided to users of eBMS data (workshop contents are available on the eBMS website). While this same package cannot be used to map species distributions, it could be expected that if this initiative were to derive spatially explicit estimates of species occurrences, it would do so by relying on the same software and open code policy. However, currently there is no open code for spatially-explicit distribution models in place within the framework of the eBMS program (**OpC** bottleneck). The eBMS has recently developed a mobile application (eBMS App) that allows volunteers to record species observations and abundances and directly upload them to the eBMS database (also to review the uploaded data). These methods are optimised for input for occupancy models, including having complete lists and therefore can be used to generate null-observations. However, data collected (species lists, tables with data observations) also get reported via email to eBMS coordinators, so data streams are not fully automated (**Auto** partial bottleneck). Funding for coordination and integration tasks is not secured both at the European and national levels; for example, the last geographic expansion of the eBMS network was possible thanks to the ABLE project, funded by the European Union Directorate General for the Environment, for a period of two years from 2018-2020 (**Fnd** bottlenecks). Data is only available upon request (**OpDat** partial bottleneck).

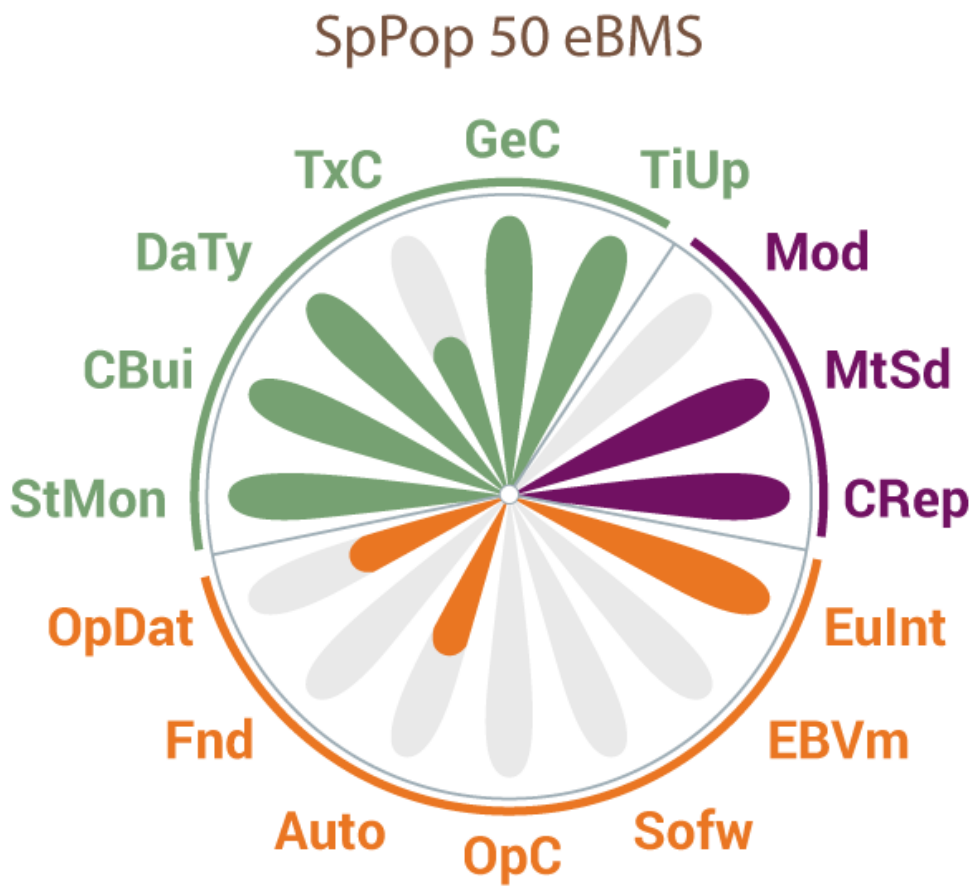

The **European Pollinator Monitoring scheme (EU PoMS)** is part of the legally mandated monitoring by Member States under the Nature Restoration Regulation (NRR) (Regulation (EU) 2024/1991), and will collect data on the status and trends of pollinator species. EU PoMS became legally binding in December 2025.

The first proposal of EU PoMS was presented in 2021, by the STING technical expert group (Potts et al. 2021). This report was based on pilot studies (2021-2023) and the emerging requirements specified under the Nature Restoration Regulation. When implemented,it will collect the data to generate this EBV since the abundance estimates, along with the location/spatial coordinates of systematic surveys will be recorded and made available for this purpose. The EU PoMS initial design has already identified current bottlenecks that could hinder the estimation of pollinator abundances across Europe which

also apply to species distribution models. In October 2024, the STING2 expert group published the Refined proposal for an EU Pollinator Monitoring Scheme (Potts et al. 2024).

The proposal of PoMS identified bottlenecks associated with the monitoring of European pollinators:

1) Data collection & sampling. The EU PoMs proposal identified more than 76 pollinator monitoring schemes already collecting pollinators' data across Europe; however, the variety of sampling methods used makes it difficult to combine the already existing data in these programs to generate pollinator indicators or to estimate abundance trends at the European level. Moreover, for some important pollinator taxonomic groups the data is limited (**TxC** partial bottleneck); this relates to the limited capacity building of some countries where there is lack of taxonomic resources and experts (**Cbui** and **GeC** partial bottlenecks). The EU PoMs proposal describes in detail how systematic surveys will be carried out for each taxonomic group (surveys are initially planned on an annual basis and assessments every 3or 6 years).

2) Models, interoperability, and IT infrastructure. Some tests have already been run on using models to develop species distributions from data collected across this monitoring network, including integrated distribution models accounting for occupancy and process-based models for predicting abundance and visitation (**Mod** partial bottleneck). The EU PoMs pilot proposal envisions data to be ultimately centralized in a repository at the EEA, European Commission (DG ENV), JRC or Eurostat, having first been collected, collated and validated by Member States. Data will be submitted or shared by Member States in a standardized form (following metadata standards) via an online platform to the European coordination facility, where pan-European analyses will be made. The EU PoMs will also put in place metadata standards to facilitate data integration, addressing the current bottlenecks of **MtSd** and **CRep**.

3) Data integration. The EU PoMs proposal presents options to set up a Pan-European pollinator monitoring network, which does not exist so far (**EuInt** bottleneck). The proposal cites the European Butterfly Monitoring (eBMS) as the closest initiative to what the EU PoMs wants to set up at the European level. Initially, EU PoMs expects to be integrating data at the national level to estimate trends in pollinators abundances and taxonomic diversity (based on the requirements of the Nature Restoration Law indicator specifications) and therefore the integration product that will be generated by EU PoMS does not match well the definition of this EBV (**EBVm** bottleneck): however, the abundance estimates collected along with the location/spatial coordinates of systematic surveys could be made available for making spatially explicit predictions of pollinators distributions. There are not yet clear guidelines about whether the code used for data integration and potentially modelling will be openly shared (although tests have been run with R, which is not a user-friendly software; **OpC** and **Sofw** bottlenecks). In the EU PoMS there is a plan to make the data openly available upon request, following the eBMS model (**OpDat** partial bottleneck). One of the actions proposed to automate data flows is the development of a pan-European internet identification platform for pollinators, which is constantly maintained and updated. Regarding funding, the NRR will imposes an obligation to monitor pollinator communities and therefore Member States will have to ensure that sufficient funding is allocated for this purpose (**Fnd** should not be a bottleneck).

   The visualisation of the EU PoMS below is based on the input from experts that worked developing the Eu PoMS. Note that this information was prepared before the EU PoMS became legally binding, and before it is implemented by the Member States. This means that there is still some uncertainty around how far the various bottlenecks will be addressed.

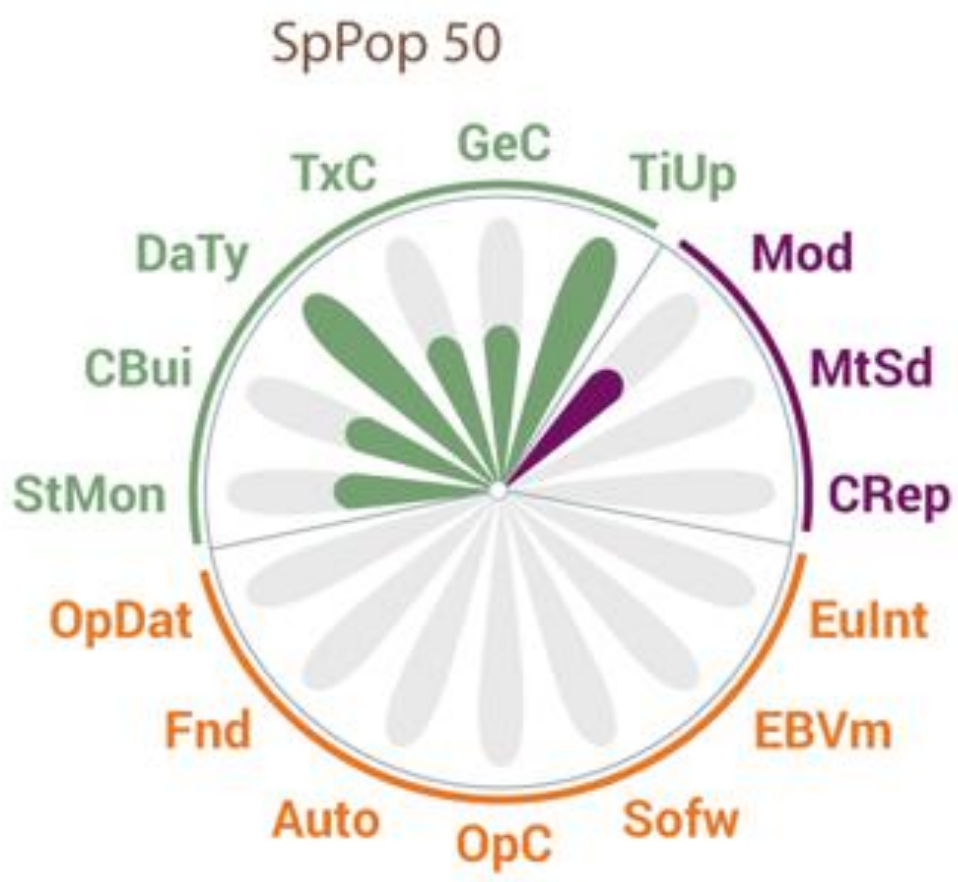

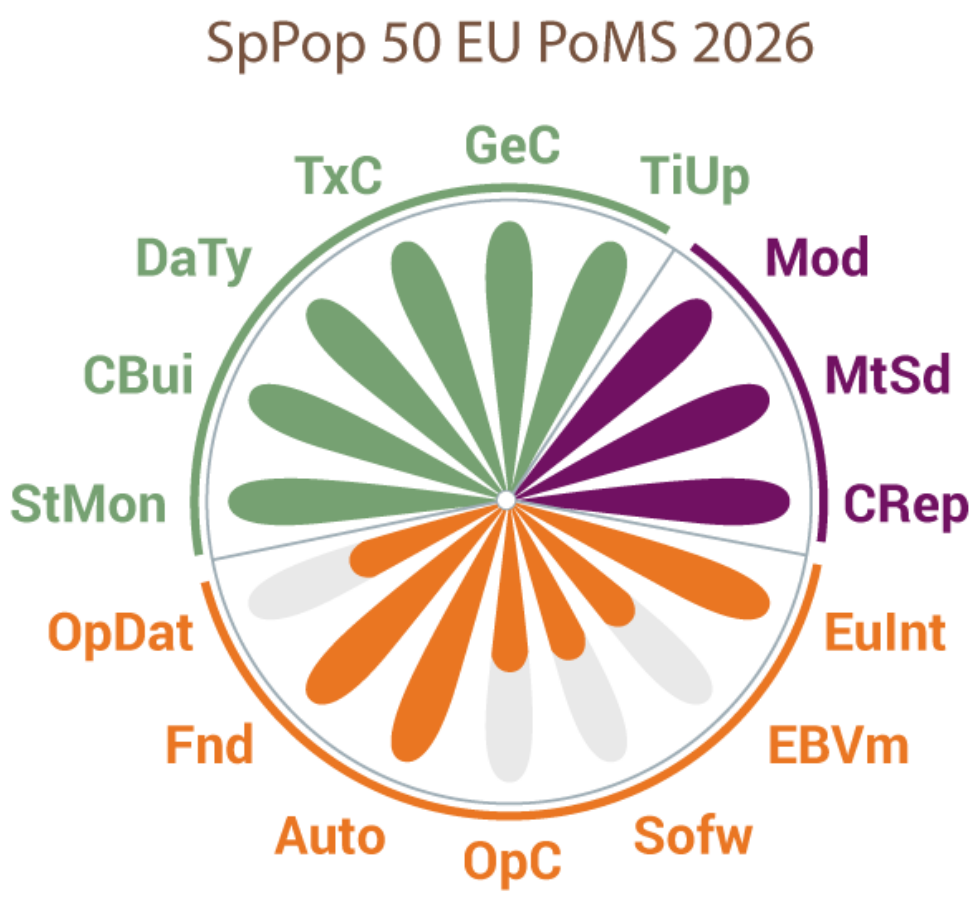

Besides pollinators and butterflies, there are no other European-level integrated standardized monitoring initiatives targeting priority invertebrates. There are examples of monitoring carried out by research networks, such as, for example, the European Swag Beetle monitoring network . This network was set up in 2008 by researchers from 8 countries and currently collects citizen science observations of the European stag beetle (*Lucanus cervus*) across 14 European countries.

References:

Potts, S.G., Dauber, J., Hochkirch, A., Oteman, B., Roy, D.B., Ahrné, K., Biesmeijer, K., Breeze, T.D., Carvell, C., Ferreira, C., FitzPatrick, Ú., Isaac, N.J.B., Kuussaari, M., Ljubomirov, T., Maes, J., Ngo, H., Pardo, A., Polce, C., Quaranta, M., Settele, J., Sorg, M., Stefanescu, C. and Vujić, A. (2021) Proposal for an EU Pollinator Monitoring Scheme, EUR 30416 EN, Publications Office of the European Union, Ispra, 2021, ISBN 978-92-76-23859-1, doi:10.2760/881843, JRC122225.

Potts, S.G., Bartomeus, I., Biesmeijer, K., Breeze, T., Casino, A., Dauber, J., Dieker, P., Hochkirch, A., Høye, T., Isaac, N., Kleijn, D., Laikre, L., Mandelik, Y., Montagna, M., Montero Castaño, A., Öckinger, E., Oteman, B., Pardo Valle, A., Polce, C., Povellato, A., Quaranta, M., Roy, D., Schweiger, O., Settele, J., Ståhls-Mäkelä, G., Tamborra, M., Troost, G., Van Der Wal, R., Vujić, A. And Zhang, J.,(2024). Refined proposal for an EU pollinator monitoring scheme, Publications Office of the European Union, Luxembourg, 2024, doi:10.2760/2005545, JRC138660.

Table S36. Terrestrial EBV factsheets: Species distributions of terrestrial plants

**EBV: Species distributions of terrestrial plants**	
ID	51
Realm	Terrestrial
EBV class	Species Populations
EBV name	Species distributions of terrestrial plants
Step in identification process	User & Policy Needs Assessment
Definition	The presence/absence or probability of occurrence of terrestrial vascular plant species within contiguous spatial units (grid cells) across the EU over time.
Metric	- Binary presence/absence<br>- Probability of occurrence
Spatial resolution unit	- Priority species: 1 × 1 km – 10 × 10 km<br>- All vascular plant species: 10 × 10 km – 50 × 50 km
Temporal resolution unit	- Priority species: 1 year<br>- All vascular plant species: 3 or 6 years
Taxonomic/ ecosystem focus group	All European terrestrial vascular plants species included in the European Red List<br>Priority terrestrial vascular plants as listed in Annex II and Annex IV of the Habitats Directive

Main European initiatives and description of current bottlenecks

The European Vegetation Archive (EVA) is an integrative database of vegetation plots across Europe. The purpose of EVA is to establish and maintain a single data repository of vegetation-plot observations (i.e. records of plant taxon co-occurrence at particular sites, also called phytosociological relevés) from Europe and adjacent areas and to facilitate the use of these data for non-commercial purposes, mainly academic research and applications in nature conservation and ecological restoration. The EVA is an initiative of the Working Group European Vegetation Survey (EVS) of the International Association for Vegetation Science (IAVS), and it is coordinated by a board of members distributed across different European institutions that gets renewed every 4 years. By April 2021, EVA comprised 99 national and supranational vegetation plots databases and contains 1,804,985 vegetation plots from 53 countries. At the end of 2021 the EVA launched ReSurveyEurope, an initiative that seeks to mobilize vegetation-plot resurvey data with repeated measurements over time and establish a collaborative initiative as a basis for nuanced and robust assessment of biodiversity trends on small spatial grains over longer periods in Europe.

Possible bottlenecks in the generation of this EBV from existing data collected by the EVA data relate to:

1) Data collection and sampling: The data gathered in ResurveyEurope comes from monitoring that follows systematic sampling protocols (data from the general EVA database also follows systematic sampling protocols but plots are sampled just once and therefore, data could somehow be considered opportunistic observations). Despite the large geographic coverage of the EVA data (53 countries), and given the enormous diversity of this taxa, it is probable not all terrestrial vascular species targeted in this EBV are covered by the vegetation plots included in the database (e.g., tree species are better covered by other initiatives such as the National Forest Inventories - see EBV "Species distributions of trees", Table S37) (**TxC** bottleneck). Moreover, the database is mostly based on single surveys conducted over the last decades in Europe and therefore represents a snapshot of abundance of vascular plants in vegetation plots so it cannot be used to generate this EBV at the desired temporal resolution (**TiUp** bottleneck). The ReSurveryEurope initiative seeks to overcome this bottleneck, by compiling temporal series data that will allow to estimate changes in distribution and abundance of vascular plants over time (it includes plots, transects or relevès

that have at least two repeated measures using the same of comparable sampling methods); however, the spatial and taxonomic coverage of ReSurveyEurope is smaller than that of the generalist EVA database. The ReSurveyEurope initiative will seek to overcome the current bottleneck in capacity building that EVA has because of its opportunistic character (**Cbui** bottleneck): it will promote the generation of time series data from EVA sampling plots in areas where they have been sampled only once.

2) Models, interoperability, and IT infrastructure. There are not bottlenecks to report in this regard: The EVA initiative has already published a series of reports commissioned by the European Environmental Agency where vegetation plots data is used to generate spatially-explicit predictions of phytosociological relevés across Europe using the Maxent model, as a measure of ecosystems distributions (Schaminée et al. 2014, 2016a,b): this method could perfectly be also applied to model individual single species (see more details of the modelling approach used below under "data integration"). To set up the EVA a software platform was developed (TurboVeg3) to facilitate data harmonization across the multiple databases on vegetation plot data collected across Europe (the software defines metadata standards for harmonization). The EVA database is centralized and curated by the EVA coordination board.

3) Data integration. There is a mismatch between the products generated by EVA and the description of this EBV, especially in terms of temporal resolution (**EBVm** partial bottleneck). The model used by EVA to model ecosystems distributions was a machine-learning presence-background model Maxent (Phillips et al. 2006, 2008). Maxent has a user-friendly interface, facilitating its use by non-modellers. Code of the models has not been made publicly available (**OpC** bottleneck). Data flows are only partially automated: contributing national and subnational databases have to upload their data using the TurboVeg3 that requires filling different tables and forms; there is no reference to the use of Apps or other software to automatically transfer data from the field to the EVA (**Auto** partial bottleneck). EVA data management has been partly funded by the Czech Science Foundation, and also partially by European-funded projects involving the staff of Masaryk University and the program developer S. Hennekens (ALTERRA, Wageningen, UR). No other information seems to be available related to current funding, but the project is ongoing and new initiatives are developed within the framework of EVA (e.g. the ReSurveyEurope was launched in 2022); however, funding for contributing parties is not warranted (**Fnd** bottleneck). The data in the EVA database is not fully open: at the time of data submission or update, the custodians assign one of the following data availability regimes to the data contributed by them, either for the whole database or its individual subsets: 1) Restricted-access data are available for data contributors only; 2) Semi-restricted-access data are available for data contributors only and 3) Free-access data are available to a wider community of users. These data can be released based on the proposal to the EVA Coordinating Board, with no need for special approval. The EVA initiative encourages a gradual transfer of data contributed to EVA from regime 1 to regime 3, but the decision on the regime is entirely upon the custodian (**OpDat** partial bottleneck).

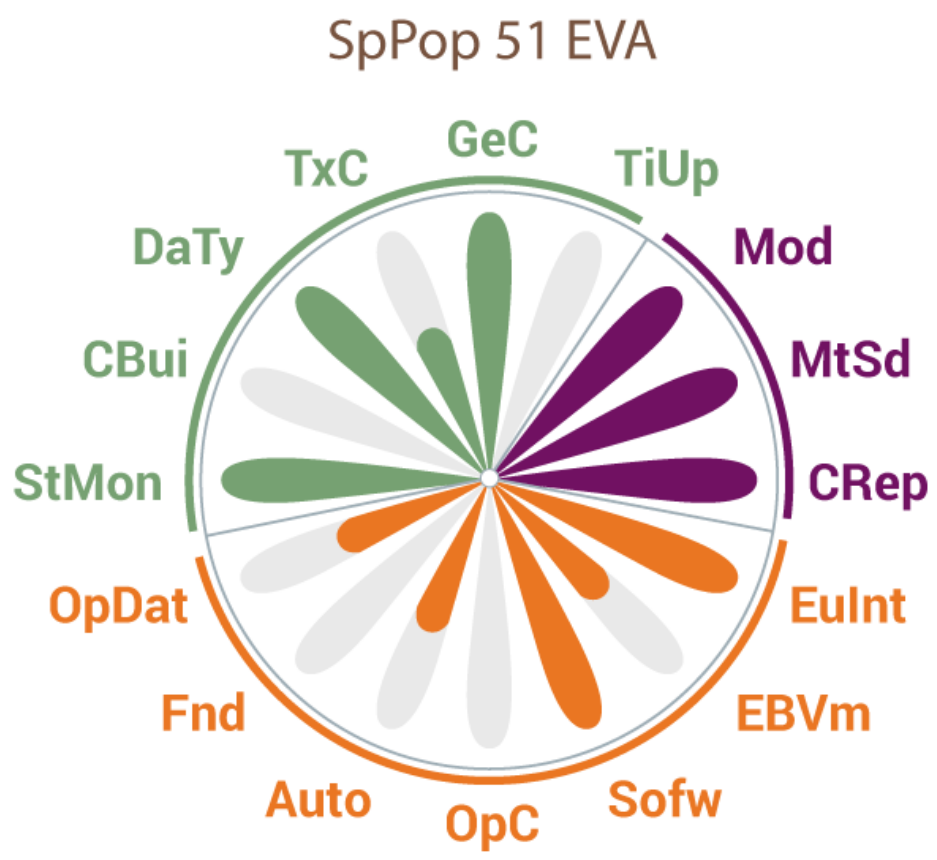

The Atlas of Flora Europea (AFE) is a long-running long-term programme for mapping the current and past distribution of vascular plants in Europe. To date it has mapped the distribution of nearly 25% of the vascular plants of Europe at a 50 x 50 km resolution in a series of book volumes starting in 1972 (**TxC** bottleneck). All the distribution maps published in the AFE vols. 1–12 were made manually. Later these maps were scanned into a digital database. For the last volumes (vol. 13–14), the distribution data have been entered directly to the database. A simple software called AFE Editor (its updated version 2010 is available from the AFE tools page) has been made and distributed among the AFE collaborators and assistants. This program facilitates electronic data recording and subsequent entering to the database. Data is centralized by LUMOUS, the Finnish Museum of Natural History, although the database containing observations per 50 km grill cell is under publishers' copyright and is not freely available to the public and only to researchers on demand (**OpDat** partial bottleneck). The low resolution of raw data - 50 km (**DaTy** bottleneck), the lack of taxonomic completeness (**TxC**), the lack of systematic data collection protocols linked to it (**StMon**), but especially the lack of temporal replication (**TiUp** bottleneck**)**, make the data of this initiative lowly suitable to the generation of this EBV.

References:

Phillips, S. J., Anderson, R. P., and Schapire, R. E. (2006) Maximum entropy modeling of species geographic distributions. *Ecological modelling*, *190*(3-4), 231-259.

Phillips, S. J. and Dudík, M. (2008) Modeling of species distributions with Maxent: new extensions and a comprehensive evaluation. *Ecography*, 31(2), 161-175.

Schaminée J.H.J., et al. (2014) Vegetation analysis and distribution maps for EUNIS habitats. Report EEA/NSV/14/006. EEA, Copenhagen. URL.

Schaminée J.H.J., et al. (2016a) Review of grassland habitats and development of distribution maps of heathland, scrub and tundra habitats of EUNIS habitats classification. Report EEA/NSV/15/005. European Environment Agency, Copenhagen. URL.

Schaminée J.H.J, et al. (2016b) Development of distribution maps of grassland habitats of EUNIS habitat classification. Report EEA/NSV/16/005. EEA, Copenhagen.URL.

Table S37. Terrestrial EBV factsheets: Species distributions of main trees

**EBV: Species distributions of main trees**	
ID	52
Realm	Terrestrial
EBV class	Species populations
EBV name	Species distributions of main trees
Step in identification process	Expert workshop
Definition	The presence/absence or probability of occurrence of European tree species within contiguous spatial units (grid cells) across the EU over time.
Metric	- Binary presence/absence<br>- Probability of occurrence
Spatial resolution unit	10 × 10 km – 50 × 50 km
Temporal resolution unit	3 or 6 years
Taxonomic/ ecosystem focus group	Tree species included in the EU-Trees4F dataset (67 species)

Main European initiatives and description of current bottlenecks

The European National Forest Inventory Network (ENFIN) is a pan-European initiative set up in 2003 with the goal of harmonizing data from National Forest Inventories (NFI) across Europe (a network of NFI organizations). ENFIN envisions a modern and responsive European system for forest monitoring that makes the best use of both field-based sample plots and remote sensing products through their appropriate combination. The ENFIN is custodian of the most comprehensive data set on in-situ tree species occurrences in Europe.
It provides support and data to other European integration initiatives such as the European Forest Data Centre and more recently to the Forest Information System For Europe (FISE). The data harmonized by ENFIN has served as a basis to map the distribution of > 250 tress species across Europe, some of them collected in the European Atlas of Forest Tree species.

Possible bottlenecks in the generation of this EBV from the ENFIN data relate to:

1) Data collection and sampling. The collection of data in NFI follows standardized monitoring protocols. The data in EU-NFI are registered in a 1 km geographical grid that does not retain the precise location of the NFI plots but can potentially be used to generate species distribution maps across the continent. ENFIN does not have capacity building on its own; this depends on the capacity building of members of the network (**CBui** bottleneck). Currently, the ENFIN group is composed of 33 different organizations from 30 countries but the UK for example is not included (**GeC** partial bottleneck). While most common tree species are monitored by all the European NFIs, information on secondary species or taxa growing only in part of the continent may be missing from some NFIs; this is often also the case for smaller trees, alien and rare species, and species for agroforestry or short-rotation forestry. However, this EBV focuses on a small set of tree species, whose data has already been harmonized and made public by members of ENFIN (Mauri et al. 2017). Moreover, the species sampled differ among countries making absences highly uncertain for some species in some countries. The frequency of data collection under NFI (time series) varies across countries but it is generally between 6 - 10 years, so the temporal resolution is smaller than the one desired for the generation of this EBV (**TiUp** bottleneck).

2) Models, interoperability, and IT infrastructure. Data from ENFIN has been integrated with other datasets to model and map the distribution of trees across Europe but this is not done routinely within the framework of the ENFIN initiative (**Mod** partial bottleneck). The ENFIN has defined MetaData Standards to harmonize data across the different National Forest Inventories (not all countries collected the same parameters in their NFI). The basic

prerequisite for the harmonization process is the common agreement on Reference Definitions that ENFIN has set.

3) Data integration. There is a mismatch between the integration achieved by ENFIN (integration of NFI databases across Europe) and the description of this EBV, especially in terms of temporal resolution (**EBVm** bottleneck). Since this integration initiative does not explicitly model species distributions over space, there is no open code or user-friendly software to report in this regard (**Sofw** and **OpC** bottlenecks). The funding of the ENFINprogram in the last few years has been secured through different European projects and framework contracts with the Joint Research Centre of the European Commission (**Fnd** partial bottleneck).; however, the funding for ENFINis not warranted in the long-term a, neither the funding of the underlying NFI depends on Member states and data partners. Data gathered at ENFIN is not available (**OpDat** bottleneck) but National Forest Inventory data is open or available upon request in many European countries contributing to ENFIN.

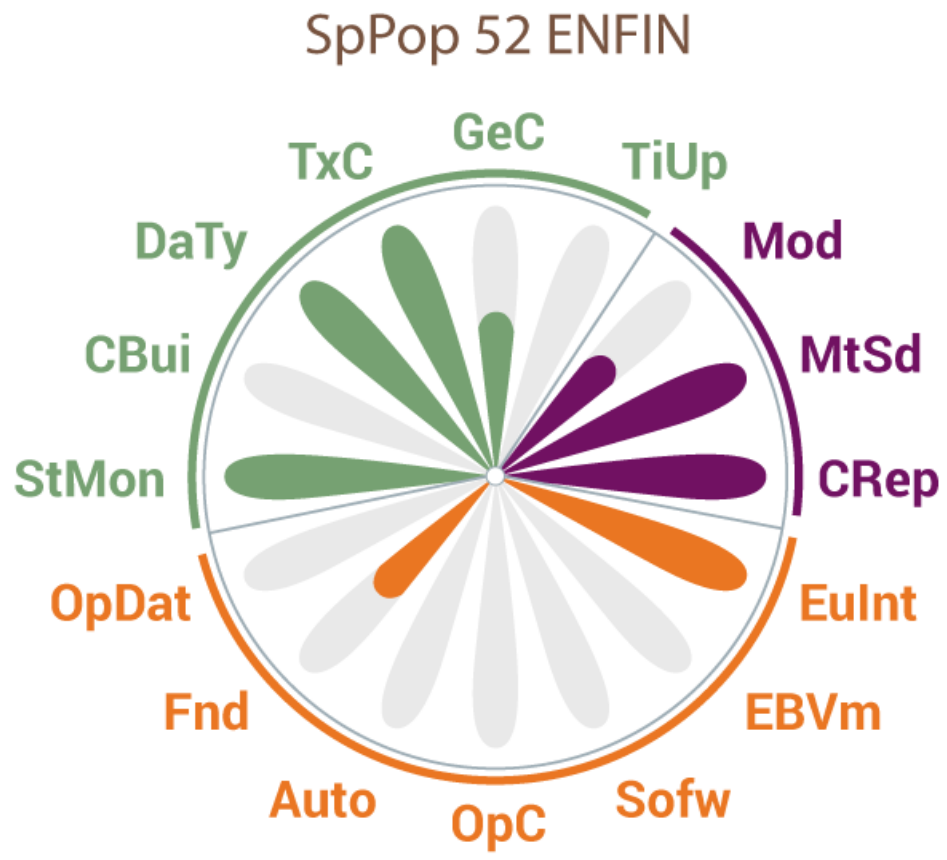

The International Co-operative Program on Assessment and Monitoring of Air Pollution Effects on Forests (ICP - Forest) has an established network of sample points that follow harmonized and standardized survey methods and an online platform for forest data storage and exchange.
Collected data in ICP-Forest database are designed in two monitoring levels:

- Level I for large scale monitoring of tree crown condition on a 16 x 16 km grid throughout Europe (approximately 600 plots) ;
- Level II for intensive monitoring of around 800 plots in selected forest ecosystems with the aim of understanding the cause-effect relationships between natural stress factors (in particular, air pollution) and forest conditions.

The resulting geo-database covers 42 countries and includes165 tree species, through more than 18,000 geo-located sample plots (active and historical).
While ICP covers major forest ecosystems in Europe, its focus is more on processes than patterns and therefore it measures parameters such as tree crown condition, foliar chemistry, tree growth, soil chemistry, etc. In this regard is more incomplete than the data available at ENFIN for the generation of this EBV.

References:

Mauri, A., Strona, G., and San-Miguel-Ayanz, J. (2017). EU-Forest, a high-resolution tree occurrence dataset for Europe. *Scientific data*, 4(1), 1-8.

Table S38. Terrestrial EBV factsheets: Species distributions of invasive alien terrestrial taxa of European concern

**EBV: Species distributions of invasive alien terrestrial taxa of European concern**	
ID	54
Realm	Terrestrial
EBV class	Species populations
EBV name	Species distributions of invasive alien terrestrial taxa of European concern
Step in identification process	User & Policy Needs Assessment
Definition	The presence/absence or probability of occurrence of invasive terrestrial species within contiguous spatial units (grid cells) across the EU over time.
Metric	- Binary presence/absence<br>- Probability of occurrence
Spatial resolution unit	1 x 1 km - 10 × 10 km
Temporal resolution unit	3 or 6 years
Taxonomic/ ecosystem focus group	Species specified in the Commission Implementing Regulation 2016/ 1141, 2017/1263, 2019/1262, 2022/1203, 2025/1422 Consolidated list at:https://eur-lex.europa.eu/legal-content/EN/TXT/PDF/?uri=CELEX:02016R1141-20220802&from=EN

Main European initiatives and description of current bottlenecks

The European Alien Species Information Network (EASIN) is an integration initiative coordinated at the European level (European Commission Joint Research Centre) that aggregates, integrates, and harmonizes spatio-temporal data for alien species (AS) and Invasive Alien Species (IAS) across Europe. The species catalogue and geodatabase are regularly updated through literature reviews, data partners inputs, and the official reports by Member States competent authorities. The EASIN Catalogue (27.07.2025) includes 10,501 terrestrial alien species, of which 61 are IAS UC (EU Regulation 1143/2014). The Catalogue's protocol provides information on the data collected for each species. EASIN facilitates the exploration of data and information from existing monitoring networks and programs available from a variety of distributed information sources by providing tools and interoperable web services, compliant with internationally recognized standards.

Possible bottlenecks in the generation of this EBV from existing data collected by EASIN data relate to:

1) Data collection and sampling. EASIN carries out the systematic collection of data on IAS in Europe, does not come exclusively from monitoring programs but from a variety of sources, including literature review, occasional observations and data portals (**StyMon** partial bottleneck); EASIN has demonstrated capacity building through different activities including: i) *ad ho*c assessments, such as the Baseline Distribution of IAS of Union concern; ii) support to the establishment of surveillance systems in compliance with EU AS policy; iii) citizen science programs, (e.g. the development of the smartphone app 'IAS in Europe' freely available and already adopted in the context of several projects and for official monitoring; iv) training to teachers and students (e.g., the MOOC course "Have you seen an alien?"), among others. EASIN collaborates with the Member States competent authorities and national experts to update and validate the datasets relevant to the EU policy context, to fulfil the EU mandate of setting up national monitoring networks for IAS; however, the extent to which this has been implemented is not fully known. Some bottlenecks regarding data collection may relate to 1) lack of communication between national coordinators & EU funded projects working on IAS in a country (e.g. LIFE projects); 2) at national level, people in charge of monitoring AS belong to different admin bodies among which there is little communication and coordination and 3) complex administrative structure in some countries (federal, regions, etc.) and how the environmental competences are distributed among them, making it difficult to harmonise and coordinate data collection tasks and to facilitate data flows and integration. Currently, the EASIN Geodatabase (04.08.2025) contains occurrence records for 14,347 species, across 64

different countries (EU & beyond) (including data for the 114 species in the consolidated list of IAS of Union concern- European Union (EU) Regulation 1143/2014). Because the data is georeferenced, it can be used to build species distribution models. The EASIN Geodatabase is updated on average 3 times/ year, but some information sources are updated less regularly, depending on effort for the update and the periodicity of their own updates. Recent EASIN database backend developments data updating will facilitate more regular updates.

2) Models, interoperability, and IT infrastructure. EASIN is not a modelling framework, therefore there is no access to modelling workflows (Mod partially bottleneck). A case study using habitat suitability models was carried out for the freshwater invasive species *Elodea nuttalli* using EASIN data across the EU (Steen et al. 2019). There is, however, regular in-house research work on IAS of Union concern to evaluate the range of expansion at both European and national levels, to understand whether niche of the species is at equilibrium in its invaded range or to predict the probability of occurrence of the species in Europe considering bioclimatic variables. EASIN makes data available under the CC-BY licence enabling reuse with attribution to the original sources through web tools for data analysis, open sharing and reporting. The openness of this data is key to redirect surveillance and trigger early warning systems. However, while standards have been defined to harmonize data across source databases, these do not necessarily apply to the underlying data (**MtSd** partial bottleneck).

3) Data integration. Data collected by EASIN are at different resolutions and aggregated at e.g., country, 10 x 10 km grid cells, partially matches the EBV (**EBVm** partial bottleneck). The EASIN Geodatabase follows a protocol to regularly retrieve data from the data partners; data are subsequently transformed and converted through the following steps: validation, cleansing and standardization, geocoding, mapping, application of quality rules and finally loaded on the Geodatabase (Datawarehouse). The EASIN Geodatabase also integrates data from the "IAS Europe'' smartphone App, which promotes the report of sightings of IAS of Union Concern by citizens, and data from the NOTSYS platform. The latter is the official tool for EU Member States to notify the Commission and inform the other Member States, as required by Reg. 1143/2014 on IIAS. In particular, the tool has been designed to facilitate a timely comprehensive notification of new detections of IAS of Union concern, related eradication measures, and their effectiveness. Funding is a bottleneck to the generation of this EBV: while the European Commission has set up EASIN as the official information system supporting Member States in the implementation of the Regulation (EU) No 1143/2014 , it only co-participate in the expenses of monitoring or eradication actions by national competent authorities, funding from the European Commission for monitoring of IAS is available via existing financial instruments such as LIFE, H2020, Cohesion or Regional Development funds. Funding is warrant in the mid - long - term to maintain the EASIN data infrastructure and the development of new data collection tools (e.g., a web base platform for reporting citizen science data) (**Fnd** partial bottleneck).

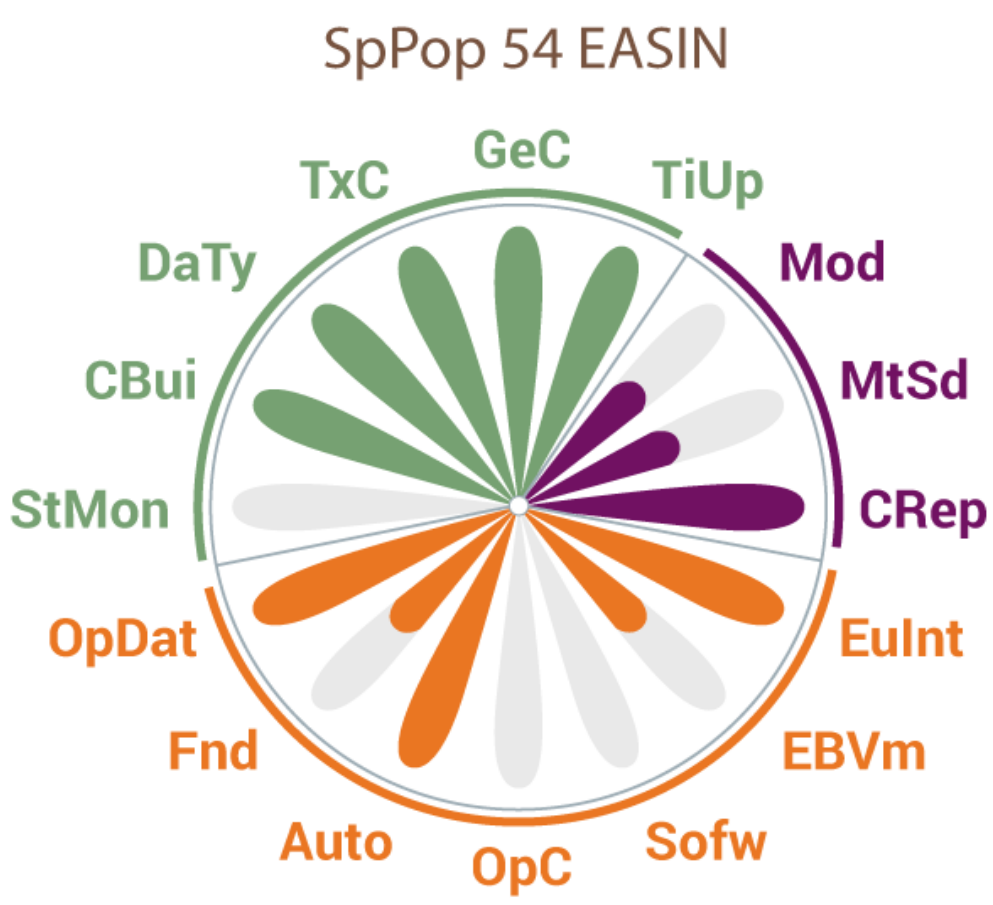

References:

Steen, B, Cardoso, A.C., Tsiamis, K., Nieto, K.,Engel J., Gervasini, E. (2019) Modelling hot spot areas for the invasive alien plant Elodea nuttallii in the EU. *Management of Biological Invasions* 10(1): 151–170. URL .

Table S39. Terrestrial EBV factsheets: Phenology of migration of birds

EBV: Phenology of migration of terrestrial birds

ID	58a
Realm	Terrestrial
EBV class	Species traits
EBV name	Phenology of migration of terrestrial birds
Step in identification process	Expert workshop
Definition	The annual timing of arrival and departure of European terrestrial migratory bird species at breeding, staging and wintering sites over time.
Metric	Migration phenology metrics such as:<br>- Day of arrival<br>- Day of departure<br>- Length of stay
Spatial resolution unit	10 × 10 km
Temporal resolution unit	1 week
Taxonomic/ ecosystem focus group	Migratory bird species defined as full migrants in the European Red List

Main European initiatives and description of current bottlenecks

There are two European-wide coordinated initiatives that collect data that could potentially be used to generate this EBV: The EuroBirdPortal (EBP) and the EURING.

The **EuroBirdPortal (EBP)** combines the data collected by the online bird recording portals operating in Europe to describe the large-scale spatiotemporal patterns of bird distributions and their changes over time. Online bird portals obtain year-round data from the intensive and widespread activities of birdwatchers. However, data is, essentially, collected using simple standardized protocols (complete lists) or no protocol at all (casual observations). As the EBP collects data from the entire year, the project has the potential to obtain at least some of the metrics required for this EBV (e.g. arrival and departure timing) for several migratory bird species.

Possible bottlenecks in the generation of this EBV from existing data collected by the EBP initiative relate to:

1) Data collection & sampling. The EBP has the potential to collect data on all bird species occurring in Europe but, currently, only the data for 137 species are stored in the EBP central data repository (**TxC** partial bottleneck). Data is collected across all European countries, but the degree of coverage is poorer in the South and, particularly, in the East and South-East. Data (species observations & counts) are gathered from online bird recording portals (e.g., ornitho) and include, essentially, both casual observations and data collected following simple systematic protocols (species lists). Only a small part of the data is collected following standardized monitoring protocols (**StMon** partial bottleneck). Training and capacity building for the network is provided during regular annual meetings or direct support to specific partners. Data is updated on a daily basis with information up to the previous day n (species lists).

2) Models, data interoperability and IT infrastructure. Despite the EBP/online bird portals data have been used to obtain some of the kind of metrics required to generate this EBV in some specific contexts (e.g. to predict the start of spring migration in the context of delimiting hunting seasons or study changes in migratory phenology; see, for example, Newton et al. 2016), the modelling of phenological traits has not been generalized nor automated within the framework of this initiative (**Mod** partial bottleneck). The EBP follows metadata standards to ensure data harmonization among its different data sources. The EBP data is harmonized, managed and stored in a central repository curated by the European Bird Census Council.

3) Data Integration. The maps featured in the EBP viewer (www.eurobordportal.org) represent aggregated observations at the weekly basis and at 30 x 30 km resolution (**EBVm** partial bottleneck); however, in the EBP central data repository the casual data are aggregated at 10 x 10 km and date and the lists are in raw format (i.e. not aggregated) with date/timing and location given as precise location or at 10 x 10 km; therefore, the data available at the EBP repository would allow the generation of this EBV at the desired spatio-temporal resolution. Although this integration initiative can produce metrics of the kind required for this EBV, there is no open code nor user-friendly software yet to report in this regard as models are not routinely used to generate phenology products from EPB data yet (**Sofw** and **OpC** bottlenecks). Data streams are automated: the local online portals collect most of their data through mobile apps in near-real time or shortly after it has been recorded in the field; data collected in the data portals are then automatically transferred to the EBP daily. The EBP main developments have been possible thanks to the support of the LIFE programme, but funding stability in the mid- long- term is not warranted (**Fnd** bottleneck). EBP data is available upon request and subject to agreement by National coordinators who hold the ownership of data (**OpDat** partial bottleneck), but just one centralized data request should be done as data is already centralized in the EBP databank (authorizations by national owners are coordinated by EBP).

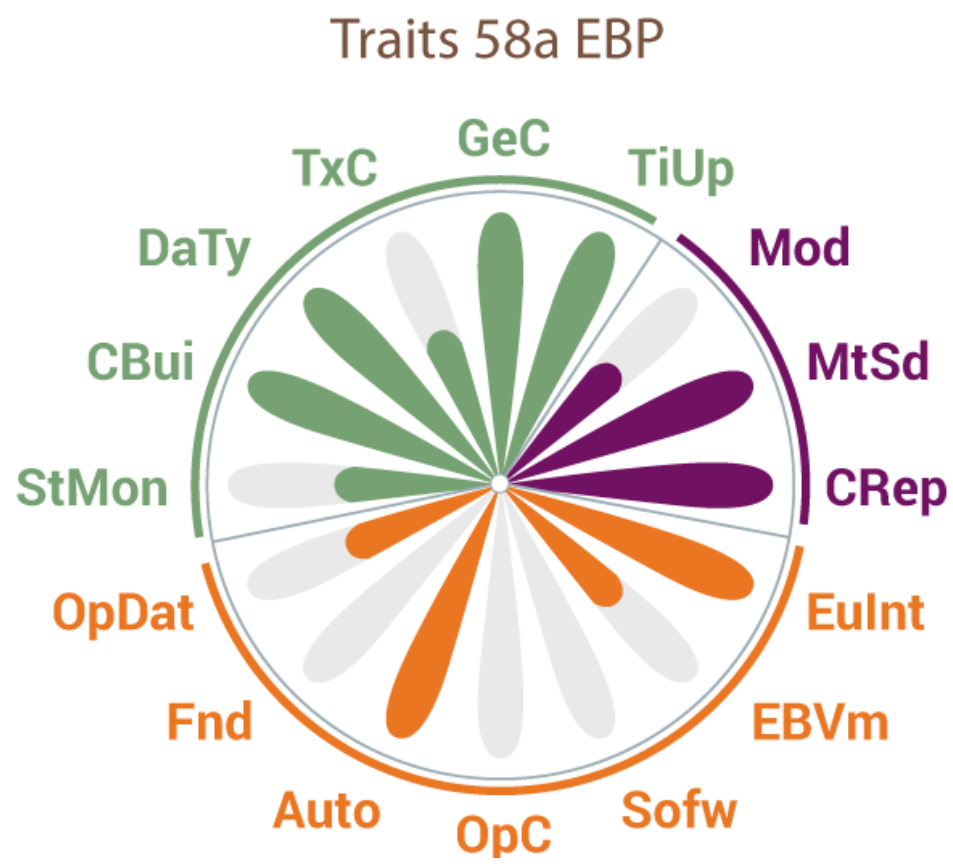

EURING coordinates bird ringing in Europe and centralizes the data collected by the different bird ringing schemes operating in the continent in order to promote and encourage its use for bird management and conservation. Ringing data are collected year-round following standardized protocols (e.g. EUROCES Constant Effort Sites) or no protocol at all. The data consist of bird ringings (when a ring was first added to a bird), recaptures (retraps of ringed birds by ringers) and recoveries/resightings (ringed bird reported by the public (e.g. dead birds, rings/marks read at a distance) which form the bulk of the data currently stored in the EURING databank (EDB). The recently launched Eurasian African Migration Atlas (Spina et al. 2022) highlights the value of the EURING data to understand bird movements in time and space while the new Migration Mapping Tool is a good example of the applied value of combining the connectivity information of EURING with the observational data from the EuroBirdPortal in single tool (Gargallo et al. 2022).

The data collected by EURING and the bird ringing schemes in general have the potential to obtain the metrics required for this EBV and, more relevantly, are probably the best suited to estimate length of stay (e.g. though mark-recapture analysis).

Possible bottlenecks in the generation of this EBV from existing data collected by EURING relate to:

1) Data collection & sampling. EURING collects data of most bird species occurring in Europe and, though the amount of data (particularly recaptures/recoveries) varies greatly among species, figures are quite high for several of them. Data is collected across all European countries, but the degree of coverage is poorer in the South and, particularly, in the East and South-East. Only part of the data is collected following standardized monitoring protocols (**StMon** partial bottleneck). Training and capacity building for the network is provided during regular annual meetings or direct support to specific partners. Data in the EURINGdataback (EDB) are mostly updated once every year (**TiUp** partial bottleneck), although each record retains its temporal resolution (the date of data collection).

2) Models, data interoperability and IT infrastructure. Ringing data has been widely used to study phenology and length of stay and to obtain some of the metrics homologous to those required by this EBV for some specific uses, for example, to estimate the start of spring migration to

delimit hunting seasons (e.g. the Migration seasons of hunted species research module of the Eurasian African Migration Atlas)(see also Ambrosini et al. 2014). However, so far, this has not been generalized nor automated within the framework of the initiative (**Mod** partial bottleneck). The EURING data flow takes place using the EURING Exchange Code standard, a pioneer of this kind among biodiversity data hubs in Europe, to ensure data harmonization and optimize its value. Data is centralized in the EURING databank.

3) Data Integration. EURING has already generated products that partially match the definition of this EBV (the Migration seasons of hunted species research module of the Eurasian African Migration Atlas, with predictions of prenuptial migration of different species at 10-day intervals - **EBVm** partial bottleneck). The data in the EDB have the required spatial and temporal resolution needed for the generation of this EBV. However, as the data is mostly updated once every year and therefore, this EBV could only be generated, at most, on a yearly basis. Though this integration initiative can produce metrics of the kind required for this EBV, there is no open code nor user-friendly software yet to report in this regard, as this modelling is not routinely integrated in this initiative (**Sofw** and **OpC** bottlenecks). Data streams are not automated yet: ringing centres across Europe send their ringing observations (data exports) once a year to the central repository manually (**Auto** bottleneck). Funding stability in the mid-long-term is not warranted (**Fnd** bottleneck). EDB data is available upon request and subject to agreement by National bird ringing schemes who hold the ownership of data (**OpDat** partial bottleneck), but just one centralized data request should be done as data is already centralized in the EDB (authorizations by national owners are coordinated by EBP).

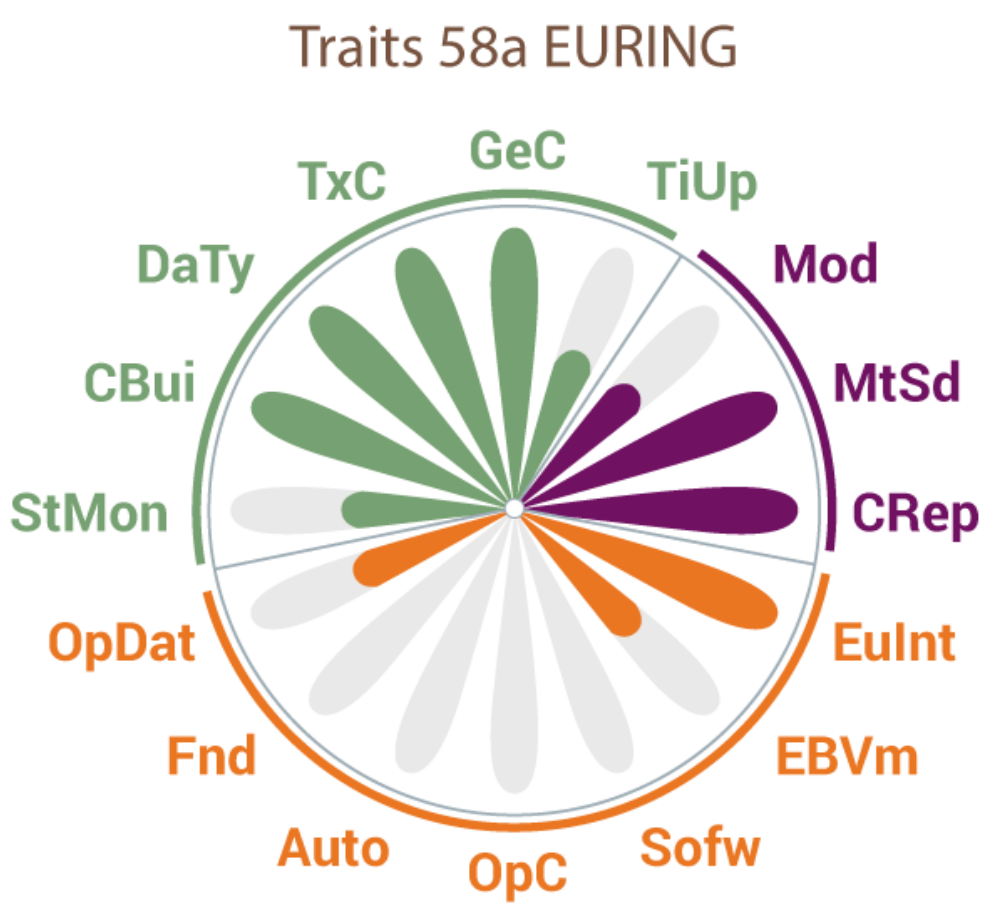

References:

Ambrosini, R., Borgoni, R., Rubolini, ... andSaino, N. (2014). Modelling the progression of bird migration with conditional autoregressive models applied to ringing data. *PloS one*, 9(7), e102440.

Gargallo, G., Davies, J.G., Faverjon, C., Kampichler C., Baillie, S.R., Cameron, A., Robinson, R.A. and Sierdsema, H. (2022) Development of a prototype early warning system for avian influenza in the EU based on risk-mapping. EFSA supporting publication 2022: EN-7762, 46 pp. URL.

Newson, S. E., Moran, N. J., Musgrove, A. J., Pearce-Higgins, J. W., Gillings, S., Atkinson, P. W., ... and Baillie, S. R. (2016). Long-term change in spring and autumn migration phenology of common migrant breeding birds in Britain: results from large-scale citizen science bird recording schemes. *Ibis (Lond. 1859), 158*, 481-495.

Spina, F., Baillie, S.R., Bairlein, F, Fiedler, W. and Thorup, K. (Eds) (2022) *The Eurasian African Bird Migration Atlas*. https://migrationatlas.org. EURING/CMS.

Table S40. Terrestrial EBV factsheets: Phenology of the emergence of butterflies

**EBV: Phenology of the emergence of butterflies**	
ID	58b
Realm	Terrestrial
EBV class	Species traits
EBV name	Phenology of the emergence of butterflies
Step in identification process	Expert workshop
Definition	The annual timing of seasonal emergence of butterflies within contiguous spatial units (grid cells) across the EU over time.
Metric	The day after which 5% of individuals have emerged
Spatial resolution unit	10 × 10 km
Temporal resolution unit	1 week (traits derived from weekly distribution data)
Taxonomic/ ecosystem focus group	Priority butterfly species listed in the Annex II and Annex IV of the Habitats Directive

Main European initiatives and description of current bottlenecks

The **European Butterfly Monitoring Scheme (eBMS)** collects abundance data for > 312 butterfly and moth species (and a few bumblebees and dragonflies) using systematic transect counts that are visited several times per year. The abundance data collected (counts per transect) gets mostly integrated to estimate population trends at the national and European levels (six indicators: grassland, forests, N2000, climate change, widespread species and urban butterflies).

Possible bottlenecks in the generation of this EBV from existing data collected by the eBMS data relate to:

1) Data collection and sampling. There are no bottlenecks to report in this regard to the generation of this EBV. eBMS focuses its efforts mostly on the collection of butterflies and moths' abundance data. Data gets collected following standardized protocols with most sampling transects being visited at least 10 times per year. Each transect (approx. 1 km length) is ideally walked every week during the butterfly flight season. If it is not possible, volunteers are encouraged to count as often as possible, every two weeks or 10 days. Besides fixed transects, currently, the eBMS also collects one-off data (opportunistic 15 min-counts that seek to encourage data collection in areas currently uncovered by fixed transects, e.g. areas of difficult accessibility). The type of data collected (species counts), and the high frequency of data sampling (weekly temporal resolution in most transects) would allow the estimation of this EBV. The geographic coverage of this monitoring program has recently been enlarged thanks to the support of a service contract from the European Union Directorate General for the Environment (ABLE project), so it currently covers 30 countries. The program promotes capacity building through the free publication of divulgation informative material for the identification of butterflies in different languages and countries, the offering of open training courses on the use of modelling tools for species trend estimations, the development of Apps to ease data collection and identification, etc.

2) Models, interoperability, and IT infrastructure. This initiative uses the ebms package to estimate population trends and calculation of trend indices/products at the national level on an annual basis (training courses and materials are available on the eBMS website); one of the intermediate steps is the calculation of flight curves using Generalized Additive Model. Currently these are used to correct abundance estimates, but information on phenology can easily be extracted (**Mod** bottleneck). The eBMS follows metadata standards that allow harmonization of the data collected across the different European countries. The eBMS database is stored and managed in a centralized repository under the custody of the Butterfly Conservation Europe and the UK Center for Ecology and Hydrology.

3) Data Integration. There is not a match between the products generated by this initiative (multi-species trend indicators; trends at the national and European level) and the EBV definition (date by which 5% of individuals have emerged) (**EBVm** bottleneck). Although this

integration initiative can produce metrics of the kind required for this EBV, there is no open code nor user-friendly software yet to report in this regard as models are not routinely used to generate phenology products from eBMS data yet but the development should be rather easy (**Sofw**, **OpC** bottlenecks). eBMS has recently developed a mobile application (eBMS App) that allows volunteers to record species observations and abundances and directly upload them to the eBMS database (also to review the uploaded data). However, data collected (species lists, tables with data observations) also get reported via email to eBMS coordinators, so data streams are not fully automated (**Auto** partial bottleneck). Data is only available upon request (**OpDat** partial bottleneck). Funding for coordination and integration tasks is not secured both at the European and national levels; for example, the last geographic expansion of the eBMS network was possible thanks to the ABLE project, funded by the European Union Directorate General for the Environment, for a period of two years from 2018-2020 (**Fnd** bottleneck). However, it is important to mention that the grassland butterfly indicator is explicitly mentioned in the Nature Restoration Regulation, and Member States will have to ensure that sufficient funding is allocated to collect data for this purpose, so Fnd should not be a bottleneck for this EBV from 2026.

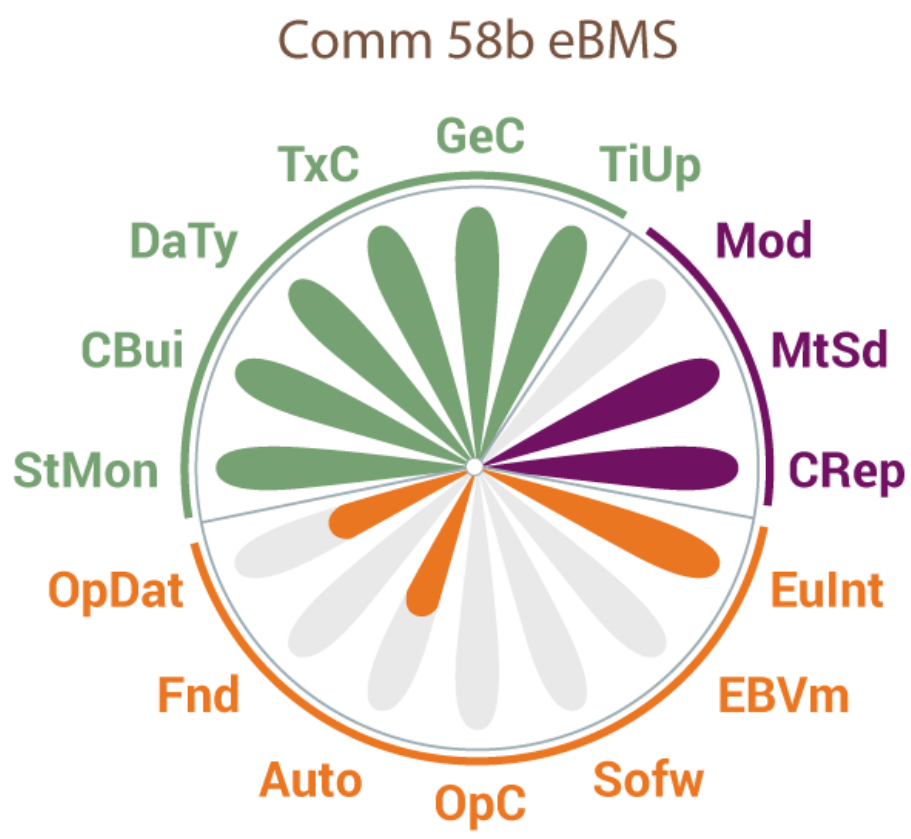

Table S41. Terrestrial EBV factsheets: Community biomass of soil microbes

**EBV: Community biomass of soil microbes**	
ID	61
Realm	Terrestrial
EBV class	Community composition
EBV name	Community biomass of soil microbes
Step in identification process	Internal review process
Definition	Estimated biomass of the living component of soil organic matter (bacteria, fungi and protozoa) within contiguous spatial units (grid cells) across the EU over time.
Metric	- Mass of microbial carbon / mass of dry soil<br>- Mass of microbial carbon / area
Spatial resolution unit	1 x 1 km
Temporal resolution unit	3 years
Taxonomic/ ecosystem focus group	Soil microbial species

Main European initiatives and description of current bottlenecks

The Land Use/Land Cover Area Frame Survey (LUCAS) collects information on land cover and land use change at the European scale. It is a project developed by EUROSTAT and it estimates the area occupied by different land use or land cover types based on observations taken at more than 250,000 sample points throughout the EU, which are visited on the ground and/or photo interpreted and classified over aerial photos and satellite images. This survey is repeated every 3 years, and it also collects a topsoil sample in a subset of all sampling points (18,000 – 26,000 points, depending on the year), which is analyzed for different chemical and biological properties. One of these properties is organic content and could potentially be used to generate this EBV.

Possible bottlenecks in the generation of this EBV from existing data collected by the LUCAS data relate to:

1) Data collection & sampling. Soil data is collected following standardized protocols for a sample of approximately 0.5 kg of topsoil (0-20 cm) in each site. Data quality checks run in parallel to the collection of the data. The LUCAS initiative has capacity building and training, with surveyors receiving training before going into the field (a set of supporting documents, instructions on how to carry out the survey, and a set of quality control procedures). Since the first LUCAS survey (2009), organic content has been one of the measured properties of these samples. However, it does not differentiate between microbial carbon and general organic carbon, so it does not really match the data type required for generating this EBV (**DaTy** bottleneck). Additionally, although it is a very extensive survey, 26.000 points across Europe might not be enough to have every microbial species represented (**TxC** partial bottleneck). The LUCAS survey is meant to be carried out at three-year intervals, in synchronization with CORINE Land Cover and the update of the High-Resolution Layers, and as such, LUCAS data matches the temporal resolution sought by this EBV.

2) Models, interoperability, and IT infrastructure. The data from the land cover and land use is corrected and run through various statistical models, but the topsoil data is not, only producing tables of organic content per location (**Mod** bottleneck). LUCAS follows metadata standards: surveyors use the same forms and instructions to integrate the data. Additionally, an ad-hoc IT tool, named Data Management Tool (DMT) is used to reinforce the standardization and integration of the LUCAS data into a central repository. The DMT records the data and analyses the quality of the recorded values through an automatic quality control.

3) Data integration. The LUCAS topsoil dataset and the derived products do not match well the definition of the EBV (overall organic content vs microbial organic content reported at the

survey site level instead of in a 1 x 1 km continuous grid; **EBVm** bottleneck). Because there are no models in place for predicting microbial organic content in a spatially-explicit way within the framework of the LUCAS project, there is no user-friendly software or open code to report in this regard (**SoFw** and **OpC** bottlenecks). Besides LUCAS data flows and gets integrated using the Data Management Tool, data flows are not fully automated (e.g., lack of data collection via APIs, visual quality checks; **Auto** partial bottleneck). The funding for LUCAS survey is warranted in the long term (Copernicus services). The data are freely available and can be downloaded after prior registration through the Request Form.

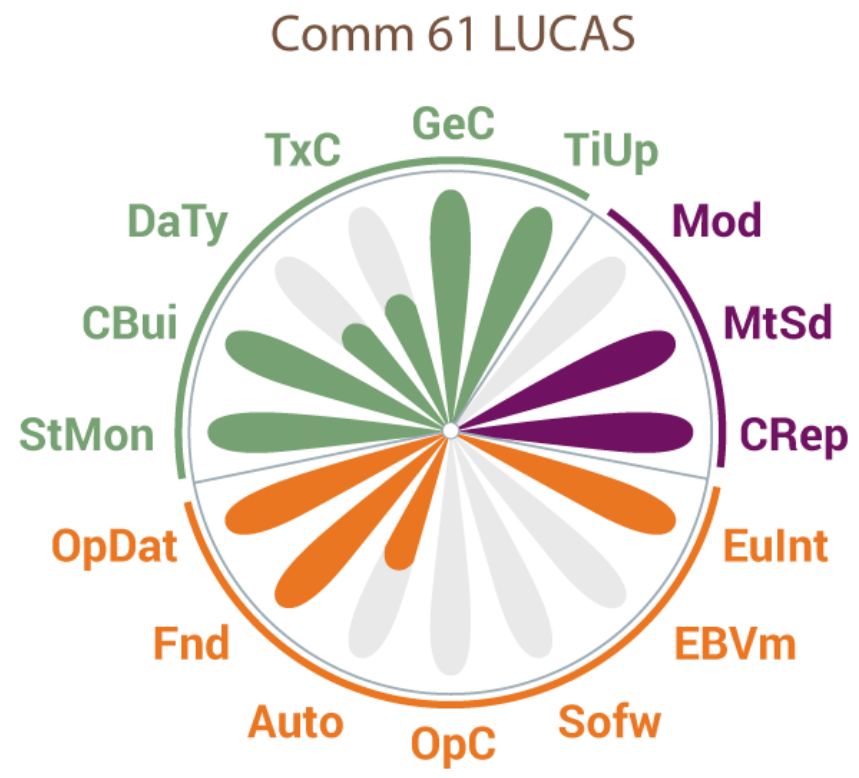

Reference:

Eurostat. (2018). LUCAS - 2018 Quality Report. URL.

Table S42. Terrestrial EBV factsheets: Community abundance and taxonomic diversity of pollinator insects

EBV: Community abundance and taxonomic diversity of pollinator insects

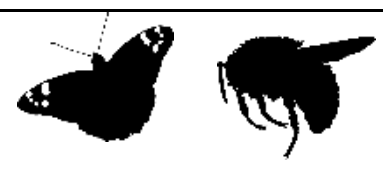

ID	62
Realm	Terrestrial
EBV class	Community composition
EBV name	Community abundance and taxonomic diversity of pollinator insects
Step in identification process	User & Policy Needs Assessment
Definition	Total amount (abundance) of pollinator insects within spatial units over time.
Metric	Predicted number of individuals of pollinator insects
Spatial resolution unit	Small regions within countries based on nomenclature of territorial units for statistics (NUTS) from Eurostat (1166 regions at NUTS 3 level)
Temporal resolution unit	1 - 5 years (rotation across years)
Taxonomic/ ecosystem focus group	All pollinator insects as proposed in the species lists of butterflies, wild bees and hoverflies of the EU Pollinator Monitoring Scheme (EU PoMS)

Main European initiatives and description of current bottlenecks

The **European Pollinator Monitoring scheme (EU PoMS)** is an ambition under development, and once implemented, will collect the data to generate this EBV since the abundance estimates, along with the location/spatial coordinates of systematic surveys, will be recorded and made available for this purpose. The monitoring of pollinator populations by EU PoMS will be key for tracking the goals set in the Nature Restoration Regulation (NRR) of halting the decline of pollinator populations by 2030 and thereby achieving an increasing trend of pollinator populations. It is expected to have its methodology approved in 2025.

The first proposal of EU PoMS was presented in 2021, by the STING technical expert group (Potts et al. 2021). In October 2024, the STING2 expert group published the Refined proposal for an EU Pollinator Monitoring Scheme (Potts et al. 2024).

The EU PoMS initial design has already identified current bottlenecks that could hinder the estimation of pollinator abundances across Europe (see also EBV n.50, Table S35). These relate to:

1) Data collection and sampling. The 2021 EU PoMs proposal identified more than 76 pollinator monitoring schemes already collecting pollinators' data across Europe; however, the variety of sampling methods used makes it difficult to combine the already existing data in these programs to generate pollinator indicators or to estimate abundance trends at the European level. Moreover, for some important pollinator taxonomic groups the data is limited (**TxC** partial bottleneck); this relates to the limited capacity building of some countries where there is lack of taxonomic resources and experts (**Cbui** and **GeC** partial bottlenecks). The EU PoMs proposal describes in detail how systematic surveys will be carried out for each taxonomic group (surveys are initially planned on an annual basis and assessments every three years).

2) Models, interoperability, and IT infrastructure. The EU PoMS initiative will aggregate community abundance and taxonomic diversity values at the national level. This will allow tracking progress on the target of reversing pollinators decline by 2030 in each Member State. The use of models to predict spatially-explicit community abundance of pollinator insects across Europe (EBV definition) is not directly contemplated in the EU PoMS proposal but this could be undertaken by organizations working with the EU PoMs data (**Mod** partial

bottleneck). The EU PoMs pilot proposal envisions data to be centralized in a repository at the EEA or European Commission, having first been collected, collated and validated by Member States. Data will be submitted or shared by Member States in a standardized form via an online platform (following metadata standards) to the European coordination facility, where pan-European analyses would be made. The EU PoMs will also put in place metadata standards to facilitate data integration, addressing the current bottlenecks of currently, none of the latter two elements exist (**MtSd** and **CRep** bottlenecks).

3) Data integration. The EU PoMs proposal presents options to set up a Pan-European pollinator monitoring network, which does not exist so far (**EuInt** bottleneck). The proposal cites the European Butterfly Monitoring (eBMS) as the closest initiative to what the EU PoMs wants to set up at the European level. The EU PoMS seeks to estimate overall abundance trends and trends of pollinator communities in each Member State and at the EU level. As such, there is a good match between the main EU PoMS products and the definition of this EBV but a mismatch in the temporal resolution (Member State and European level vs subnational level; **EBVm** partial bottleneck). There are not yet clear guidelines about whether the code used for data integration and potentially modelling will be openly shared (although tests have been run with R, which is not a user-friendly software; **OpC** and **Sofw** bottlenecks). It will put in place metadata standards to facilitate data integration and there is a plan to make the data openly available upon request, following the eBMS model (**OpData** partial bottleneck). One of the actions proposed to automate data flows is the development of a pan-European internet identification platform for pollinators, which is constantly maintained and updated. The monitoring of pollinator populations by EU PoMS will prove key to tracking the goals set in the proposed Nature Restoration Regulation of halting the decline of pollinator populations by 2030 and achieve thereafter an increasing trend of pollinator populations. The NRR is set to impose an obligation to monitor pollinator communities and therefore EU Member States would have to ensure that sufficient funding is allocated for this purpose (therefore the change in **Fnd** bottleneck).

The visualisation of the EU PoMS below is based on the input from experts that worked developing the Eu PoMS. Note that this information was prepared before the EU PoMS became legally binding, and before it is implemented by the Member States. This means that there is still some uncertainty around how far the various bottlenecks will be addressed.

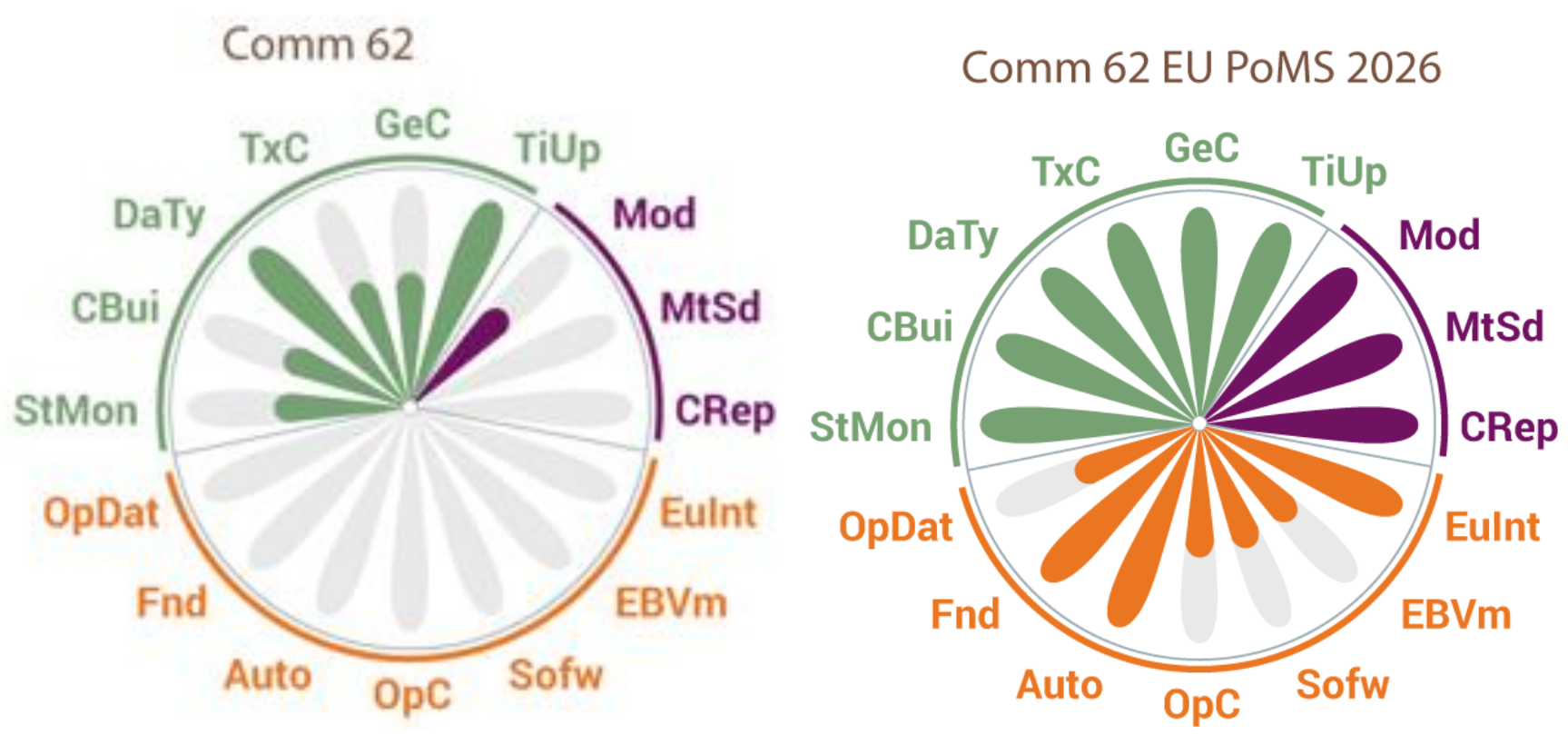

References:

Potts, S.G., Dauber, J., Hochkirch, A., Oteman, B., Roy, D.B., Ahrné, K., Biesmeijer, K., Breeze, T.D., Carvell, C., Ferreira, C., FitzPatrick, Ú., Isaac, N.J.B., Kuussaari, M., Ljubomirov, T., Maes, J., Ngo, H., Pardo, A., Polce, C., Quaranta, M., Settele, J., Sorg, M., Stefanescu, C. and Vujić, A. (2021) Proposal for an EU Pollinator Monitoring Scheme, EUR 30416 EN, Publications Office of the European Union, Ispra, 2021, ISBN 978-92-76-23859-1, doi:10.2760/881843, JRC122225.

Potts, S.G., Bartomeus, I., Biesmeijer, K., Breeze, T., Casino, A., Dauber, J., Dieker, P., Hochkirch, A., Høye, T., Isaac, N., Kleijn, D., Laikre, L., Mandelik, Y., Montagna, M., Montero Castaño, A., Öckinger,

E., Oteman, B., Pardo Valle, A., Polce, C., Povellato, A., Quaranta, M., Roy, D., Schweiger, O., Settele, J., Ståhls-Mäkelä, G., Tamborra, M., Troost, G., Van Der Wal, R., Vujić, A. And Zhang, J.,(2024). Refined proposal for an EU pollinator monitoring scheme, Publications Office of the European Union, Luxembourg, 2024, doi:10.2760/2005545, JRC138660.

Table S43. Terrestrial EBV factsheets: Aerial biomass of migrating birds, bats, and insects

EBV: Aerial biomass of migrating birds, bats, and insects

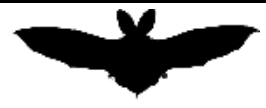

ID	63
Realm	Terrestrial
EBV class	Community composition
EBV name	Aerial biomass of migrating birds, bats and insects
Step in identification process	User & Policy Needs Assessment
Definition	Biomass flows of aerial migrants (birds, insects and bats) across Europe within contiguous spatial units (grid cells) over time.
Metric	Summary statistics of migration densities of birds derived from vertical profile time series of weather radar data (e.g., hourly averages of bird density and speed)
Spatial resolution unit	1 x 1 km - 10 x 10 km
Temporal resolution unit	1 day
Taxonomic/ ecosystem focus group	All migratory birds, bat and insect species (by size class)
Main policy targets	Habitats Directive; Birds Directive

Main European initiatives and description of current bottlenecks

The Aerial biomass of migrating birds, bats and insects can be extracted from European weather radars. The Aloft data portal is a community-driven initiative that aims to provide access to biological data that comes from European weather radars. It is maintained by members of the radar aeroecology community, coming from several research networks and projects, including the European Network for the Radar surveillance of Animal Movement (ENRAM) , GloBAM, CROW and HiRAD. These networks have long-term interdisciplinary collaborations with the Operational Programme for the Exchange of Weather Radar Information (OPERA) and national meteorological services, and their work resulted in a data license agreement to use the weather data for ecological research, producing the two most comprehensive datasets of aerial biological data in Europe (Desmet et al. 2025). The data can be used to map mass movements of birds and insects in the air across Europe. The datasets are deposited on Zenodo and can also be explored in the Aloft data portal.

The main bottlenecks to the generation of this EBV from meteorological data relate to:

1) Data collection & sampling. The management of weather radar data across Europe is operated by OPERA. OPERA collects *clean* and *unclean* polar volume data with a very high temporal frequency (every 5–15 minutes): the former serves to generate meteorological and hydrological products (e.g., precipitation forecasts) and the latter can be used to extract biological information (so in this regard, the data collected serves the generation of this EBV). The ENRAM and GLoBAM networks have used this unclean polar volume data to produce summary statistics and maps depicting distributions of migration intensity, flight direction, altitude, and ground speed of avian migrants at various spatial and temporal scales (the use of this data to extract information about insects or bats is still under development (Nussbaumer et al. 2021); **TxC** partial bottleneck). However, OPERA changed its data exchange policies to prioritize data with meteorological applications so the data flows of unclean polar volume data are no longer available for many countries via the central repository, jeopardizing the long-term maintenance of the biological applications of the radar network (Shamoun-Baranes, J. et al. 2022) (with only a few steps data is converted into aerial bird abundance, **DaTy** partial bottleneck). These changes in data sharing policy by OPERA may limit the capacity building of ENRAM and GloBAM partners, who had developed multiple initiatives since the setting of the ENRAM network (**CBui** partial bottleneck).

2) Models, interoperability, and IT infrastructure. Data from OPERA has been broadly used within the ENRAM and GLoBAM networks to model animal movement, estimate aerial biomass of birds and insects, forecast bird migration peaks, etc. OPERA centralizes data collected by

national radar networks and standardizes it to develop, generate and distribute high-quality pan-European weather radar composite products on an operational basis. Not all data sent to OPERA is in the same format or the files are not always structured in the same way and thus there is need to do some extra work in order to process the weather radar data for biological products with current standard tools used by GloBAM (e.g. the R package *bioRAD;* Dokter et al. 2019); Similarly data is not always harmonized across radars, especially the uncleaned data so there is likely need to do more work to harmonize data across radars and countries to extract biological relevant data from radar images (**MtSd** partial bottleneck).

3) Data integration. The weather radar data is well integrated at the European level, but it is still not quite complete for extracting biodiversity data (**EuInt** partial bottleneck). The products generated by the ENRAM and GloBAM networks match well the definition of this EBV in terms of product type and temporal resolution, but a rasterized representation of biomass flows using weather radar data is currently available only for birds (**EBVm** partial bottleneck); moreover, currently the ENRAM/GloBAM summarize data around a buffer between 5 –25 km from the radar or 5–35 km from the radar. Yet with interpolation methods developed by Nussbaumer et al. (2019), it is possible to create products at a resolution of 10 km (value on the lower range of the spatial resolution sought for this EBV). To translate the radar data to bird metrics, one of the common tools used by ENRAM and GloBAM in Europe is to use the R package *bioRad* (an R package; Dockter et al. 2019). In the following step, this data is used to develop models (for example predictive models of migration (Kranstauber et al. 2022, Lippert et al. 2022)), which also often developed in R, which despite being open software, requires advanced technical knowledge for its use. However, the Aloft data portal has a user friendly data visualisation interface (**Sofw** partial bottleneck). One of the GloBAM project goals is to set up an automated data processing pipeline running on cloud infrastructure to retrieve biological information from European and US weather radar data as these become available, with the capacity to run over the long-term (i.e., to automatize the full data flow from retrieving radar data to providing estimates of the abundance of migrating species). While the funding for the OPERA infrastructure is secured in the long term given it is a meteorological facility, the funding for the GloBAM network is not secured in the long term and strongly dependent on the availability of research funding at the European scale (**Fnd** partial bottleneck). One of the goals of the GloBAM network is to make information on aerial migrations available as open data, supporting researchers in accessing, processing and analyzing weather radar data and vertical profiles of aerial migrants. The data on vertical profiles of birds from weather radar volume scans for over 100 radars (and from where aerial biomass can be inferred) are available via GitHub repository and Zenodo. Some countries are now adopting open data policies and have their polar volume radar data now freely available (i.e., the raw weather radar data but not yet converted into biological data).

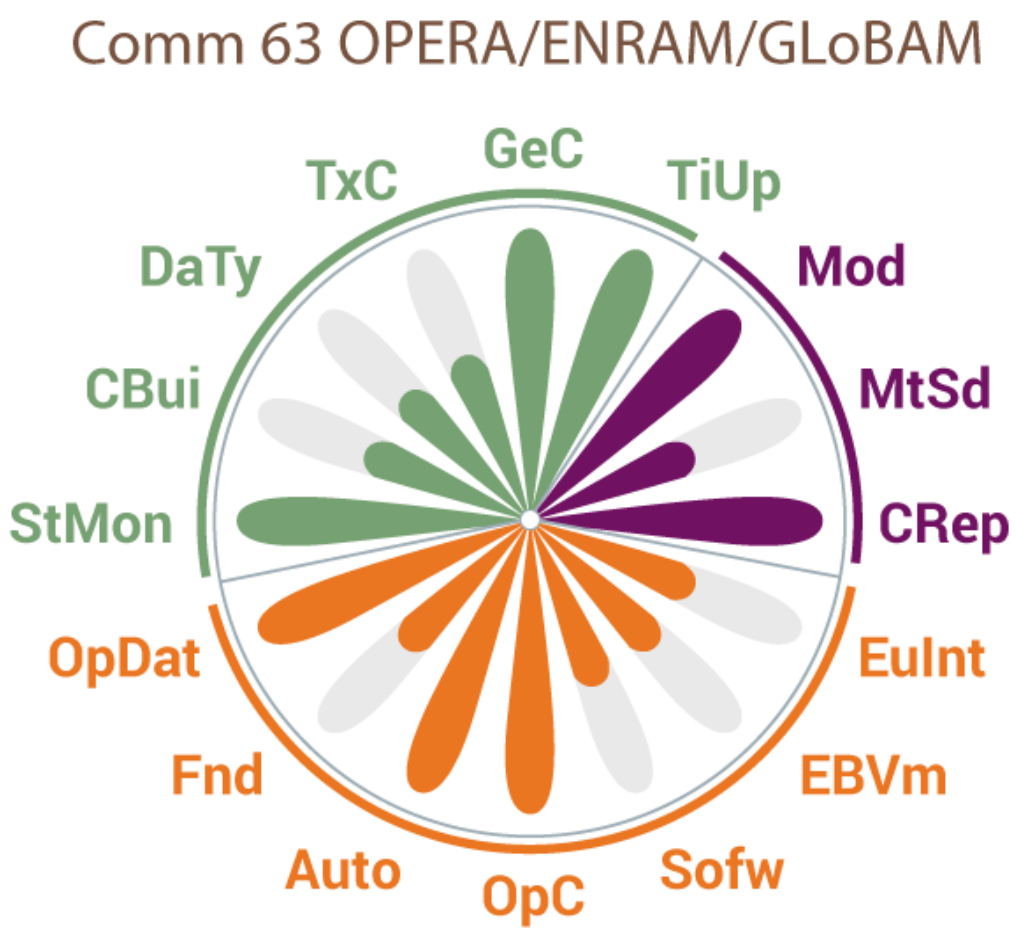

References:

Desmet, P., Shamoun-Baranes, J., Kranstauber, B. *et al.* Biological data derived from European weather radars. *Sci Data* **12**, 361 (2025). https://doi.org/10.1038/s41597-025-04641-5

Dokter, A. M., P. Desmet, J. H. Spaaks, S. van Hoey, L. Veen, L. Verlinden, C. Nilsson, G. Haase, H. Leijnse, A. Farnsworth, W. Bouten and J. Shamoun-Baranes (2019). "bioRad: biological analysis and visualization of weather radar data." *Ecography,* 42(5): 852-860.

Kranstauber, B., Bouten, W., van Gasteren, H., and Shamoun-Baranes, J. (2022). Ensemble predictions are essential for accurate bird migration forecasts for conservation and flight safety. Ecological Solutions and Evidence 3, e12158.

Lippert, F., Kranstauber, B., Forré, P.D., and van Loon, E.E. (2022). Learning to predict spatiotemporal movement dynamics from weather radar networks. Methods in Ecology and Evolution 13, 2811-2826.

Nussbaumer, R., Benoit, L., Mariethoz, G., Liechti, F., Bauer, S., & Schmid, B. (2019). A geostatistical approach to estimate high resolution nocturnal bird migration densities from a weather radar network. *Remote Sensing*, *11*(19), 2233.

Nussbaumer, R., Schmid, B., Bauer, S., and Liechti, F. (2021). A Gaussian Mixture Model to Separate Birds and Insects in Single-Polarization Weather Radar Data. Remote Sensing 13, 1989.

Shamoun-Baranes, J., Bauer, S., Chapman, J. W., Desmet, P., Dokter, A. M., Farnsworth, A., ... & Leijnse, H. (2022). Meteorological data policies needed to support biodiversity monitoring with weather radar. *Bulletin of the American Meteorological Society*, 103(4), E1234-E1242.

Table S44. Terrestrial EBV factsheets: Functional composition of soil biota

**EBV: Functional composition of soil biota**	
ID	64
Realm	Terrestrial
EBV class	Community composition
EBV name	Functional composition of soil biota
Step in identification process	User & Policy Needs Assessment
Definition	The functional composition and diversity of soil biota based on morphological, physiological, phenological and behavioral traits or functional/taxonomic groups.
Metric	- Functional group richness<br>- Functional diversity indices
Spatial resolution unit	1 x 1 m
Temporal resolution unit	1 year
Taxonomic/ ecosystem focus group	Bacteria, fungi, protozoa, collembola, mites, earthworms, larval and adult insects (e.g. Hymenoptera, Coleoptera and Diptera larvae), myriapods, spiders, mollusks and crustaceans

Main European initiatives and description of current bottlenecks

The Land Use/Land Cover Area Frame Survey (LUCAS) collects information on land cover and land use change at the European scale. It is a project developed by EUROSTAT and it estimates the area occupied by different land use or land cover types on the basis of observations taken at more than 250,000 sample points throughout the EU, which are visited on the ground and/or photo interpreted and classified over aerial photos and satellite images. This survey is repeated every 3 years, and it also collects a topsoil sample in a subset of all sampling points across the EU (18,000 – 26,000 points, depending on the year), which is analyzed for different chemical and biological properties. One of these properties, related to the EBV, is soil biodiversity through DNA sequencing.

Possible bottlenecks in the generation of this EBV from existing data collected by the LUCAS topsoil data relate to:

1) Data collection & sampling. Soil data is collected following standardized protocols for a sample of approximately 0.5 kg of topsoil (0-20 cm) in each site. Data quality checks run in parallel to the collection of the data. The LUCAS initiative has capacity building and training, with surveyors receiving training before going into the field (a set of supporting documents, instructions on how to carry out the survey, and a set of quality control procedures). Since the 2018 survey, DNA metabarcoding is used to analyse the soil diversity of the microbial community in a sample of 1,000 surveyed sites. This analysis targets the following attributes: Bacteria and Archaea (16S rDNA), Fungi (ITS), Eukaryotes (18S rDNA), Microfauna (nematodes), Mesofauna (arthropods), Macrofauna (earthworms), Metagenomics. Given the small sample of sites and the large diversity of microbes, it is hard to assume that every microbial taxon is represented in the analyses (**TxC** partial bottleneck); genetic analyses do not inform about abundance of each taxon (**DaTy** bottleneck). The LUCAS survey is done every 3 years, a smaller frequency than the temporal resolution sought to generate this EBV (**TiUp** bottleneck).

2) Models, interoperability, and IT infrastructure. LUCAS follows metadata standards: surveyors use the same forms and instructions to integrate the data. Additionally, an ad-hoc IT tool, named Data Management Tool (DMT) is used to reinforce the standardization and integration of the LUCAS data into a central repository. The DMT records the data and analyses the quality of the recorded values through an automatic quality control. The data from the land cover and land use is corrected and run through various statistical models, but models are not used to generate functional diversity indices (**Mod** bottleneck).

3) Data integration. The LUCAS integration initiative does not produce the functional diversity indices aimed at in this EBV, and it does not even collect data on microbial diversity at the desired temporal resolution (**EBVm** bottleneck). Because there are no models in place for the generation of these indices, there is no user-friendly software or open code to report in this regard (**SoFw** and **OpC** bottlenecks). Besides LUCAS data flows and gets integrated using the Data Management Tool, data flows are not fully automated (e.g., lack of data collection via APIs, visual quality checks; **Auto** partial bottleneck). The data are freely available and can be downloaded after prior registration through the Request Form.

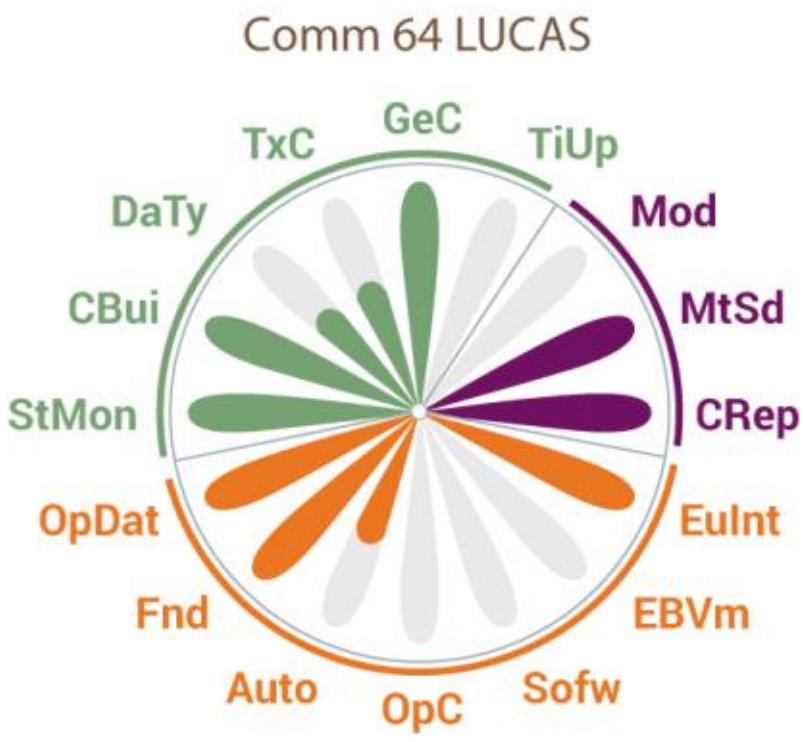

References:

Eurostat (2018). LUCAS - 2018 Quality Report. URL.
Joint Research Centre (JRC) (2018). LUCAS - Soil 2018. URL.

Table S45. Terrestrial EBV factsheets: Ecosystem distribution of terrestrial EUNIS Habitats

EBV: Ecosystem distribution of terrestrial EUNIS Habitats

ID	66
Realm	Terrestrial
EBV class	Ecosystem structure
EBV name	Ecosystem distribution of terrestrial EUNIS Habitats
Step in identification process	User & Policy Needs Assessment
Definition	The geographical/spatial distribution of terrestrial EUNIS habitats within contiguous spatial units (grid cells) across the EU over time.
Metric	- Binary presence/absence<br>- Probability of occurrence
Spatial resolution unit	10 x 10 km
Temporal resolution unit	1 year
Taxonomic/ ecosystem focus group	EUNIS terrestrial habitats (e.g. level 3 or 4)

Main European initiatives and description of current bottlenecks

The European Vegetation Archive (EVA) is an integrative database of vegetation plots across Europe. The purpose of EVA is to establish and maintain a single data repository of vegetation-plot observations (i.e., records of plant taxon co-occurrence at particular sites, also called phytosociological relevés) from Europe and adjacent areas and to facilitate the use of these data for non-commercial purposes, mainly academic research and applications in nature conservation and ecological restoration. The EVA is an initiative of the Working Group European Vegetation Survey (EVS) of the International Association for Vegetation Science (IAVS), and it is coordinated by a board of members distributed across different European institutions that gets renewed every 4 years. By April 2021, EVA comprised 99 national and supranational vegetation plots databases and contains 1,804,985 vegetation plots from 53 countries. At the end of 2021 the EVA launched ReSurveyEurope, an initiative that seeks to mobilize vegetation-plot resurvey data with repeated measurements over time and establish a collaborative initiative as a basis for nuanced and robust assessment of biodiversity trends on small spatial grains over longer periods in Europe. Data from EVA has already been used to generate spatially-explicit predictions of distributions of a few EUNIS grassland, shrub and forest habitats across Europe (Schaminée et al. 2014, 2016a,b).

Possible bottlenecks in the generation of this EBV from existing data collected by the EVA data relate to:

1) Data collection and sampling: The data gathered in ResurveyEurope comes from monitoring that follows systematic sampling protocols (data from the general EVA database also follows systematic sampling protocols, but plots are sampled just once and therefore, data could somehow be considered opportunistic observations). The geographic coverage of the EVA data is very broad (53 countries) and vegetation plots are sampled across a broad gradient of ecosystems in Europe, so potentially the EVA/ReSurveyEurope databases contain enough information as to map the distribution of most if not all EUNIS terrestrial habitats. However, the database is mostly based on single surveys conducted over the last decades in Europe and therefore represents a snapshot of abundance of vascular plants in vegetation plots so it cannot be used to generate this EBV at the desired temporal resolution (**TiUp** bottleneck). The ReSurveryEurope initiative seeks to overcome this bottleneck, by compiling temporal series data that will allow to estimate changes in distribution and abundance of vascular plants over time (it includes plots, transects or relevès that have at least two repeated measures using the same of comparable sampling methods); however, the spatial and taxonomic coverage of ReSurveyEurope is smaller than that of the generalist EVA database. The ReSurveyEurope initiative will seek to overcome the current bottleneck in capacity building that EVA has

because of its opportunistic character (**Cbui** bottleneck): it will promote the generation of time series data from EVA sampling plots in areas where they have been sampled only once.

2) Models, interoperability, and IT infrastructure. There are no bottlenecks to report in this regard: The EVA initiative has already published a series of reports commissioned by the European Environmental Agency where vegetation plots data is used to generate spatially-explicit predictions of EUNIS habitats distributions across Europe using the Maxent model (Schaminée et al. 2014, 2016 a,b). To set up the EVA database, a software platform was developed (TurboVeg3) to facilitate data harmonization across the multiple databases on vegetation plot data collected across Europe (the software defines metadata standards for harmonization). The EVA database is centralized and curated by the EVA coordination board.

3) Data integration. There is a partial mismatch between the products generated by EVA and the description of this EBV, especially in terms of temporal resolution (**EBVm** partial bottleneck). The model used by EVA to model EUNIS habitats' distributions was a machine-learning presence-background model Maxent (Phillips et al. 2006, 2008). Maxent has a user-friendly interface,facilitating its use by non-modellers. Code of the models has not been made publicly available (**OpC** bottleneck). Data flows are only partially automated: contributing national and subnational databases must upload their data using the TurboVeg3 that requires filling different tables and forms; there is no reference to the use of Apps or other software to automatically transfer data from the field to the EVA (**Auto** partial bottleneck). EVA data management has been partly funded by the Czech Science Foundation, and partially by European-funded projects involving the staff of Masaryk University and the program developer S. Hennekens (ALTERRA, Wageningen, UR). No other information seems to be available related to current funding, but the project is ongoing and new initiatives are developed within the framework of EVA (e.g., the ReSurveyEurope was launched in 2022); however, funding for contributing parties is not warranted (**Fnd** bottleneck). The data in the EVA database is not fully open: at the time of data submission or update, the custodians assign one of the following data availability regimes to the data contributed by them, either for the whole database or its individual subsets: 1) Restricted-access data are available for data contributors only; 2) Semi-restricted-access data are available for data contributors only and 3) Free-access data are available to a wider community of users. These data can be released based on the proposal to the EVA Coordinating Board, with no need for special approval. The EVA initiative encourages a gradual transfer of data contributed to EVA from regime 1 to regime 3, but the decision on the regime is entirely upon the custodian (**OpDat** partial bottleneck).

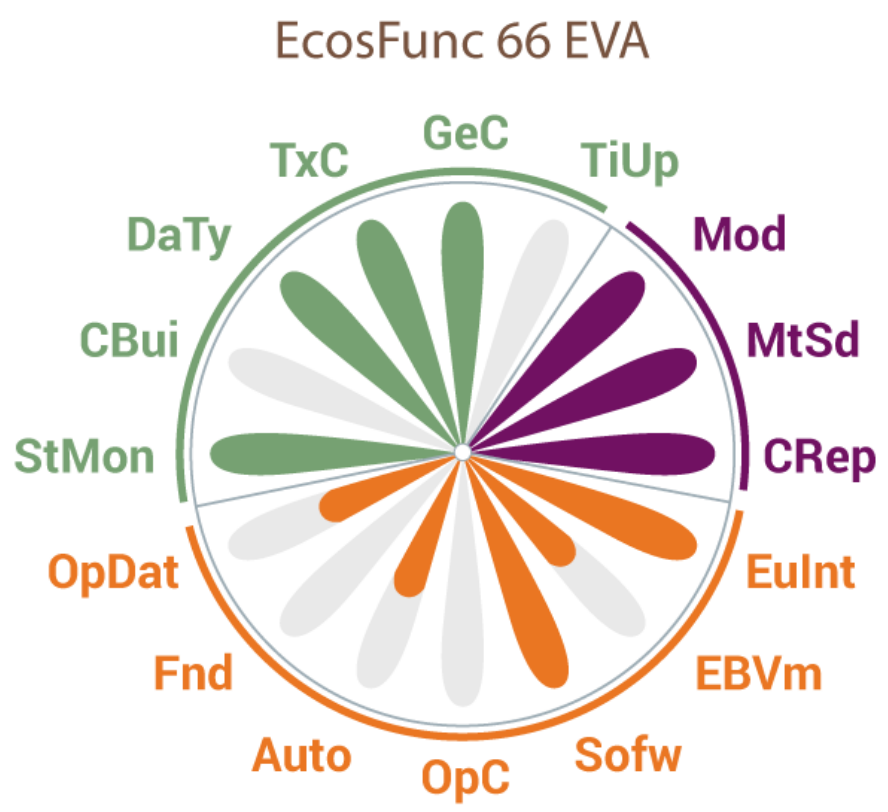

References:

Phillips, S. J., Anderson, R. P., and Schapire, R. E. (2006) Maximum entropy modeling of species geographic distributions. *Ecological modelling*, *190*(3-4), 231-259.

Phillips, S. J., Dudík, M. (2008) Modeling of species distributions with Maxent: new extensions and a comprehensive evaluation. *Ecography*, 31(2), 161-175.

Schaminée J.H.J., et al. (2014) Vegetation analysis and distribution maps for EUNIS habitats. Report EEA/NSV/14/006. EEA, Copenhagen. URL.

Schaminée J.H.J., et al. (2016 a) Review of grassland habitats and development of distribution maps of heathland, scrub and tundra habitats of EUNIS habitats classification. Report EEA/NSV/15/005. European Environment Agency, Copenhagen. URL.
